# Prediction and Analysis of Protein Hydroxyproline and Hydroxylysine

**DOI:** 10.1371/journal.pone.0015917

**Published:** 2010-12-31

**Authors:** Le-Le Hu, Shen Niu, Tao Huang, Kai Wang, Xiao-He Shi, Yu-Dong Cai

**Affiliations:** 1 Institute of Systems Biology, Shanghai University, Shanghai, China; 2 Department of Chemistry, College of Sciences, Shanghai University, Shanghai, China; 3 Key Laboratory of Systems Biology, Shanghai Institutes for Biological Sciences, Chinese Academy of Sciences, Shanghai, China; 4 Institute of Health Sciences, Shanghai Institutes for Biological Sciences, Chinese Academy of Sciences and Shanghai Jiao Tong University School of Medicine, Shanghai, China; 5 Centre for Computational Systems Biology, Fudan University, Shanghai, China; University of South Florida College of Medicine, United States of America

## Abstract

**Background:**

Hydroxylation is an important post-translational modification and closely related to various diseases. Besides the biotechnology experiments, in silico prediction methods are alternative ways to identify the potential hydroxylation sites.

**Methodology/Principal Findings:**

In this study, we developed a novel sequence-based method for identifying the two main types of hydroxylation sites – hydroxyproline and hydroxylysine. First, feature selection was made on three kinds of features consisting of amino acid indices (AAindex) which includes various physicochemical properties and biochemical properties of amino acids, Position-Specific Scoring Matrices (PSSM) which represent evolution information of amino acids and structural disorder of amino acids in the sliding window with length of 13 amino acids, then the prediction model were built using incremental feature selection method. As a result, the prediction accuracies are 76.0% and 82.1%, evaluated by jackknife cross-validation on the hydroxyproline dataset and hydroxylysine dataset, respectively. Feature analysis suggested that physicochemical properties and biochemical properties and evolution information of amino acids contribute much to the identification of the protein hydroxylation sites, while structural disorder had little relation to protein hydroxylation. It was also found that the amino acid adjacent to the hydroxylation site tends to exert more influence than other sites on hydroxylation determination.

**Conclusions/Significance:**

These findings may provide useful insights for exploiting the mechanisms of hydroxylation.

## Introduction

Many proteins undergo a wide variety of post-translational modifications. Reversible modifications are thought to be relevant in physiological processes, while non-reversible modifications may contribute to pathological situations and diseases [1]. Hydroxylation is one of the important protein reversible post-translational modifications. During the chemical process of hydroxylation, amino acid residue is modified by the attachment of at least one hydroxyl group. Hydroxylation of amino acid side chains in proteins is less common than other post-translational modifications [2]. Up until now, proline is the main amino acid residue to be hydroxylated in proteins, which is intensively modified in collagen [3]. The proline hydroxylation occurs at the γ-C atom, forming hydroxyproline, which is an essential element of collagen, and can stabilize the triple helix structure in turn a necessary element of collagen protofibrils. Proline hydroxylation is also an essential component of hypoxia response via hypoxia inducible factors [4], [5], [6]. Ascorbate deprivation causes deficiencies in proline hydroxylation, making collagen less stable, which can associated with metabolic disorder or disease [7]. The second type of protein hydroxylation residue is lysine, also intensively modified in collagen [8], [9], which could also be hydroxylated on its δ-C atom, forming hydroxylysine. It's relevant to both secretion and function in the extracellular matrix [10]. Some of lysine hydroxylation sites are then subsequently glycosylated by UDP-galactose through secretary pathway [11], [12] which is necessary for immuno-determinants in T cell recognition [13], [14].

Experimental identification of hydoxylated proteins with proline or lysine sites, commonly using mass spectrometric method [10], [15], [16], is quite difficult, time-consuming and expensive. By comparison, in silico prediction methods are time-saving and cost-saving. However, there is only one bioinformatics approach regarding the prediction of the hydroxylation modification, which used the bio-kernel SVM model to predict the 37 sequences collected from NCBI [17], [18] and achieved the specificity of 70% and the sensitivity of 90%, but it limited to the prediction of the collagen hydroxyproline [19]. Therefore more universal computational methods should be developed to annotate the hydroxylation sites of the abundant newly discovered proteins in the post-genome era. And the methods may be helpful to understand the complicated molecular mechanism of hydroxylation.

In this work, we presented a new general algorithm to predict proline and lysine hydroxylation sites based on 506 amino acid indices [20], [21] (AAindex), Position-Specific Scoring Matrices [22] (PSSM) and structural disorder [23], [24] features. AAindex depicts the physicochemical properties and biochemical properties of amino acids. PSSM represents the conservation information of the protein in evolution. Proteins that lack fixed secondary and/or tertiary structures under physiological conditions are defined as intrinsically disordered proteins. Intrinsic disorder regions (IDRs) are abundant in many eukaryote proteins [25], [26]. To our knowledge, most IDRs are related to the key biological activities [27], [28], [29] and various diseases [30], [31], [32], [33]. A number of PTMs are strongly associated with intrinsic disorder [34], [35], [36], [37], [38] and many PTMs (e.g. phosphorylation, lipidation, GPI-anchor) have been experimentally proved to be correlated with IDRs [35], [37]. For example, macromolecular interactions can be modulated with the acetylation and methylation of lysine residues in histones, which change the physico-chemical properties of intrinsically disordered core domains [28]. In view of this, the intrinsic disorder was used as a new feature to recode the amino acids. The prediction model were built using incremental feature selection (IFS) method [39], [40] and evaluated by jackknife cross-validation. Based on the optimal feature sets, the relationships between the features and protein hydroxylation sites were also discussed.

## Materials and Methods

### Benchmark Dataset

We retrieved hydroxylated proteins from UniProt/Swiss-Prot [41] (Release: 57.12, 15-Dec-2009) by searching “hydroxyproline” or “hydroxylysine” in the field “modified residue”. To build a high quality benchmark dataset, the entries with hydroxylation annotation confidence - “probable”, “potential”, or “by similarity” were excluded. As a result, the hydroxyproline dataset consisted of 100 protein sequences and the hydroxylysine dataset consisted of 28 protein sequences.

Within the hydroxyproline dataset, there were 678 experimentally validated hydroxylated proline residues and 3403 non-hydroxylated proline residues. Then we extracted peptides with 13 residues that consisted of a proline residue, 6 residues upstream and 6 residues downstream of the proline residue. The 678 peptides containing the hydroxylated proline residues were assigned as positive samples, while 1356 peptides that were randomly selected from the 3403 peptides containing non-hydroxylated proline residues were assigned as negative samples (see Table S1). Similarly, 108 positive samples and 216 negative samples were obtained from the hydroxylysine dataset (see Table S2).

### Peptides Coding

In this research, peptides were coded by three kinds of features: amino acid index, PSSM conservation, and structural disorder.

#### Amino Acid Index

Amino Acid Index (AAindex, http://www.genome.ad.jp/aaindex/) [20], [21] database is a collection of numerical indices that stand for diverse physicochemical properties and biochemical properties of amino acids. For each amino acid, there are 506 indices representing its different physicochemical and biological properties. Therefore, the physicochemical properties and biochemical properties of amino acid can be represented by a 506-D (dimensional) vector. Moreover, those indices belong to 5 clusters: alpha and turn propensities, beta propensity, composition, hydrophobicity, physicochemical properties.

#### PSSM Conservation

Protein conservation always indicates biology function, and post-translational modifications are prone to occur in the conservative protein segments. Here, we employed Position Specific Iterated BLAST [42] (PSI-BLAST), a powerful sequence searching method, to quantify the sequence conservation with Position Specific Scoring Matrix (PSSM) [22] which has been proved to be effective in the identification of other post-translational modification sites [43], [44]. It depicts the conservation of each amino acid residue in the sequence by a 20-D vector, the element of which measures the likelihood that the residue mutates to each of the 20 amino acids. Thus, a protein with *X* amino acid residues will take a 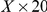 matrix as its PSSM. The parameters of PSI-BLAST (Release 2.2.12) used to generate PSSM were set as following: expectation value 0.0001, e-value threshold for inclusion in multipass model 0.0001, maximum number of passes in multipass version 3. And The alignment database was UniRef100 (Release: 15.9) which contains 9,385,165 reference clusters.

#### Structural Disorder

Disorder structures are often rich in binding sites which are important loci for diverse post-translational modifications such as acetylation, methylation and phosphorylation [35]. Therefore, we utilized the disorder feature of protein sequence to code the peptides. VSL2 [45], one of the best predictors for disorder, was used to weight the likelihood of each amino acid residue to be disordered in the sequence. The disorder score calculated by VSL2 for each residue ranges from 0 to 1. The larger the score is, the more likely the residue lacks fixed structure.

#### Feature Space

Because the middle residues of the peptides of the hydroxyproline dataset or hydroxylysine dataset shared the common 506 amino acid indices, these middle residues were thus coded by 20 PSSM conservation scores and 1 disorder score, totally 21 features. Other residues (6 amino acids upstream and 6 amino acids downstream) can be represented by 506 amino acid indices, 20 PSSM conservation scores, and 1 disorder score, totally 527 features. Overall, each peptide consisting of 13 amino acid residues could be coded by a 6,345-D (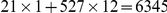) vector. That is to say, the feature space is 6,345-D.

### Model Constructing

First, we used Maximum Relevance, Minimum Redundancy [46] (mRMR) method to rank the 6,345 features according to their importance. Then based on the rank of features, we generated 500 feature sets from the top 500 features. For each feature set, a prediction model was constructed with nearest neighbor algorithm and evaluated by jackknife cross-validation. The incremental feature selection method was used to select the optimal feature set with the best prediction performance. The model based on the optimal feature set was chosen as the final prediction model.

#### Feature Prioritizing

Maximum Relevance, Minimum Redundancy [46] (mRMR) method was always employed to sort the features in descending order in bioinformatics [47], [48], [49], [50]. As its name tells, it contains two criteria: the Max-Relevance criterion and the Min-Redundancy criterion. Max-Relevance criterion requires that the preferentially selected features possess more correlation with target than other features, while Min-Redundancy criterion demands that the feature to be selected possesses minimal redundancy with the already selected features. By applying the Max-Relevance criterion, the features are ranked in the MaxRel feature list according to the descending order. By applying both the criteria, the features that are strongly correlated with target and lowly redundant to the already selected features are preferentially selected, and the features are prioritized in the mRMR feature list. The principle of the algorithm can be found in Peng's original study [46], and the program can be retrieved from the web site http://penglab.janelia.org/proj/mRMR/index.htm.

#### Nearest Neighbor Algorithm

Nearest neighbor algorithm (NNA) is one of the widely used machine learning algorithms. In NNA, an unclassified sample is predicted to share the common class as its nearest neighbor. The distance between two samples is calculated as follows
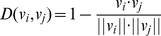
(1)where 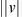 represents the module of sample vector 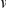, and 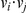 represents the dot product of two sample vectors.

Suppose a data set consisting of *n* classified peptides with a corresponding coding vector set 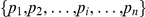. For a query peptide with coding vector 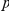, its class will be predicted to be same as the class of the peptide whose coding vector 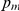 subjects to 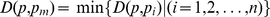
(2)


#### Evaluation

In this research, jackknife cross-validation [51], [52], [53] was employed to evaluate the performance of the constructed NNA predictors since it has been widely used to evaluate diverse classifiers [54], [55], [56], [57]. In the validation, each sample is removed in turn from the data set as a test sample, and then predicted by the model trained with the rest data. Four sophisticated measurements: sensitivity (Sn), specificity (Sp), accuracy (AC) and matthews correlation coefficient (MCC) were utilized to assess the capability of the NNA predictors. Sn, Sp and AC represent the success rates of prediction on positive, negative and overall datasets respectively. MCC is always introduced when the positive and negative datasets are out-of-balance from each other. It varies from -1 to 1, and the larger MCC is, the better the predictor performs. These four measurements can be formulated as follows
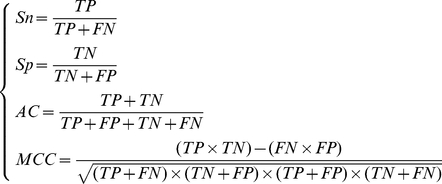
(3)where TP, FP, TN and FN denotes the numbers of true positive, false positive, true negative, false negative samples, respectively.

#### Incremental Feature Selection

After prioritizing the features in the feature space by the mRMR method, the next step is to determine that which features should be selected to construct the NNA predictor with best performance. In this research, Incremental Feature Selection [39], [40] (IFS) method was utilized to solve this problem.

Incremental Feature Selection (IFS), an effective feature selection method based on the mRMR method. According to the *N* ranked features in mRMR feature list, *N* feature sets could be built as follows
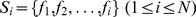
(4)where 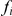 denotes the *i-th* ranked feature in the mRMR feature list.

According to each feature set, the peptides in the dataset were recoded into numerical vectors. Based on each new coding vector set, nearest neighbor algorithm was applied to construct the prediction model. By the jackknife cross-validation, the prediction accuracies for the two datasets were then calculated. IFS curve was plotted with the number of features in the feature set 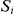 as x-axis and the prediction accuracy as y-axis. The optimal feature set was selected when the IFS curve rose to the peak. And the model on the optimal feature set was used as the ultimate tool to predict the hydroxylation sites of proteins.

## Results and Discussion

### The sorted features by mRMR

After the representation of the peptides, we obtain the sorted features in MaxRel feature list and mRMR feature list for the hydroxyproline dataset and hydroxylysine dataset (see Table S3 and Table S4) by applying the mRMR procedure. The MaxRel feature list consists of the 500 preferentially selected features, where a small index of a feature means that the feature is highly correlated with the class label. The mRMR feature list also consists of the 500 preferentially selected features, where a small index of a feature implies that the feature is very important for separating the hydroxylated sites and the non-hydroxylated sites.

### Performance of NNA predictors

**Figure 1:**
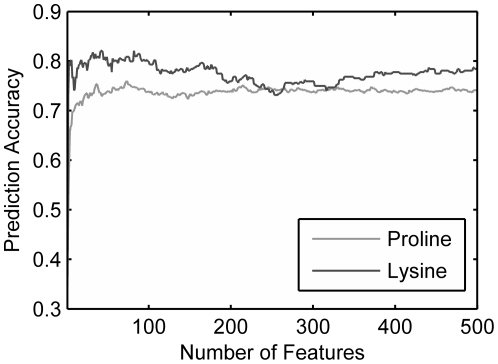
IFS curves for hydroxyproline dataset and hydroxylysine dataset. Each curve shows that prediction accuracies of the 500 predictors evaluated by the jackknife cross-validation.

Based on the 500 ranked features in the mRMR feature list, we built 500 feature sets according to Eq. (4). Then a predictor was constructed for each feature set using nearest neighbor algorithm and then evaluated by the jackknife cross-validation. The performances of the 500 predictors for the hydroxyproline dataset and hydroxylysine dataset are shown in the IFS curves (**Figure 1**). For hydroxyproline dataset, the curve arrives at the peak with the prediction accuracy of 76.0% and the corresponding optimal feature set consists of the first 73 features in the mRMR feature list. And the Sn, Sp and MCC are 64.8%, 81.6% and 0.461, respectively. For hydroxylysine dataset, the curve arrives at the peak with the prediction accuracy of 82.1% and the corresponding optimal feature set consists of the first 42 features in the mRMR feature list. And the Sn, Sp and MCC are 70.4%, 88.0% and 0.592, respectively. The performances of the NNA predictors for the two datasets are also listed in Table S5 and Table S6, respectively.

### Feature analysis

For the hydroxyproline dataset or hydroxylysine dataset, biological feature analysis was done on two feature sets: (i) Feature set A: the 500 sorted feature in the MaxRel feature list, which are highly related to protein hydroxylation in the feature space. (ii) Feature set B: the optimal feature set, with which the predictor has the best performance for identifying the hydroxylation sites.

#### Hydroxyproline Feature Sets

**Figure 2:**
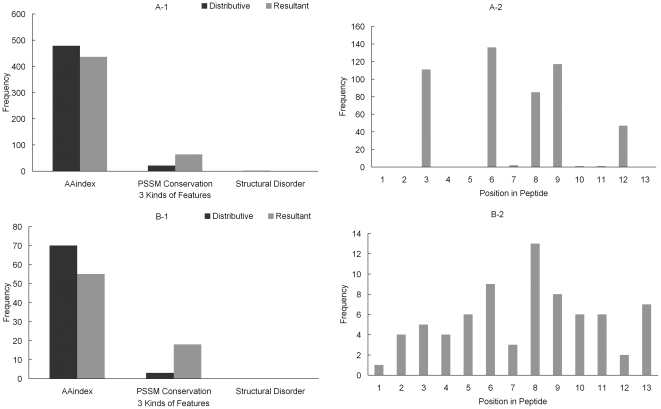
Distribution of the three kinds of features and distribution of 13 positions of the peptides in feature set A and B for hydroxyproline dataset. Legend “Distributive” means that the frequency of each kind of features are calculated according to the proportion of each kind of features in the 6,345 features (e.g., in dataset A, there should be 478 (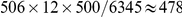) amino acid factors, 21 (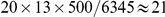) conservation, and 1 (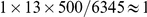) disorder); while legend “Resultant” represents the frequency of each kind of features in the dataset (A or B).

**Figure 2** depicts the distribution of the three kinds of features and the distribution of the 13 positions of sequence fragment in feature set A and B for hydroxyproline dataset. Legend “Distributive” describes the frequency of each kind of features which are calculated according to the composition of the three kinds of features in the 6,345 features (6,072 amino acid indices, 260 PSSM conservation, 13 disorder); while legend “Resultant” stands for the number of each kind of features in the feature set (A or B). In **Figure 2A**-1****, the frequency of resultant AAindex is a little lower than the frequency of distributive AAindex; while the number of the resultant PSSM conservation is 64, much higher than the number of the distributive PSSM conservation (21); and there is no difference between the frequency of resultant disorder feature and distributive disorder feature. For the feature set B, the distribution shown in **Figure 2B**-1**** is similar to the distribution of the feature set A. This may suggest that the evolution information play an irreplaceable role for proline hydroxylation. We also select surrounding sites of the hydroxylation sites to investigate the influence of these sites on the determination of the hydroxylation. The position specific distribution of the peptides in the feature sets are shown in **Figure 2A-2 and 2B-2**. In **Figure 2A-2**, the AA3 (the 3^rd^ amino acid of the peptide), AA6, AA8 and AA9 are highly correlated to the proline-hydroxylation. In the MaxRel feature list (see Table S3), the first 100 features contains 83 features of AA6, which strongly indicates the extremely important role of AA6 in proline hydroxylation. In **Figure 2B-2**, AA6, AA8, and AA9 are also distinct from other amino acids. Therefore, the characteristic of the amino acids adjacent to middle proline tends to exert more influence on the identification of hydroxylated proline residues than the relatively distal residue in the peptides. Crystal structures of prolyl hydroxylases show that the catalytic PHD_2_ domain of in complex with the C-terminal oxygen-dependent degradation domain of HIF-1a suggests that PHD catalysis needs a mobile region that located near the hydroxylation site and stabilizes the PHD2·Fe(II).2OG complex [58]. That somehow mirrors that the nearby sequence of targeting hydroxylated proline fit for the interaction could be important for hydroxylation mechanism.

**Figure 3:**
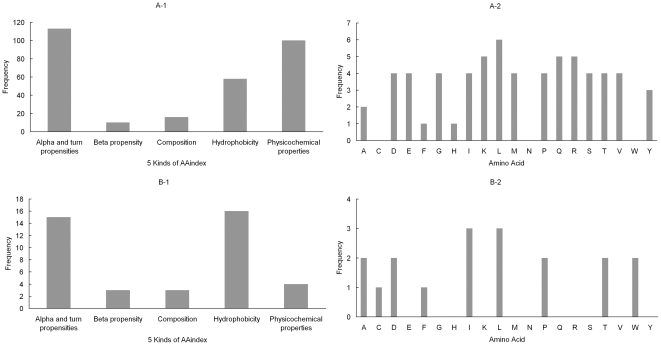
Distribution of the 5 feature clusters of the AAindex and distribution of conservation of 20 amino acids in the feature set A and B for hydroxyproline dataset.

**Figure 3** depicts the distribution of the 5 feature clusters of the AAindex and the distribution of conservation of 20 amino acids in the two feature sets. **Figure 3A**-1**** and **Figure 3B**-1**** show that all the 5 kinds of AAindex contribute to the hydroxylating of proline residue. Alpha and turn propensities and physicochemical properties are two important attributes related to the hydroxylation among the AAindex. Alpha and turn propensities and hydrophobicity are more important in determining hydroxylated proline residues than other properties. That is indeed in consistence with triple helical collagen structure, with half of prolines have been processed to 4-OH-proline to make up the structure [59]. The 4-OH-proline sides chains point away from the helix and hydrogen bond with the hydrophobic state to the solvent [60]. That is also essential in stabilizing the triple helical conformation of collagen providing hydrogen bonds and water bridges related with structural hydrophobicity [61]. These post-translational hydroxylations catalyzed by collagen prolyl hydroxylases are required for proper collagen biosynthesis, folding, and assembly. From **Figure 3A**-2****, we can see that all the PSSM conservation features are highly related to the hydroxylation except conservation of cysteine, asparagine, tryptophan. Among the 18 PSSM conservation features in the feature set B (**Figure 3B**-2****), the mutations of isoleucine and leucine contribute more than other features in the breakdown of hydroxylated sites and non-hydroxylated sites.

#### Hydroxylysine Feature Sets

**Figure 4:**
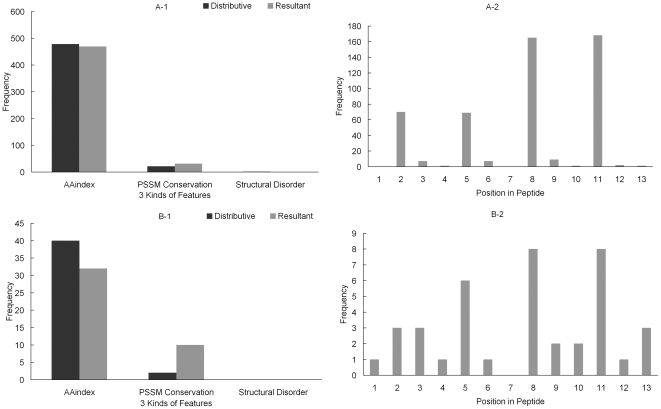
Distribution of the three kinds of features and distribution of 13 positions of the peptides in feature set A and B for hydroxylysine dataset. Legend “Distributive” means that the frequency of each kind of features are calculated according to the proportion of each kind of features in the 6,345 features (e.g., in dataset A, there should be 478 (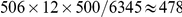) amino acid factors, 21 (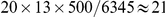) conservation, and 1 (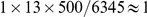) disorder); while legend “Resultant” represents the frequency of each kind of features in the dataset (A or B).

**Figure 4** shows the distribution of the three kinds of features and the distribution of 13 positions of sequence fragment in feature set A and B for hydroxylysine dataset. As is shown in the **Figure 4A**-1**** and **4B-1**, the differences between resultant and distributive features in hydroxylysine dataset are similar to the differences in hydroxyproline dataset. AA8 and AA11 are noticeable in both position specific distributions of feature set A and B shown in **Figure 4A**-2**** and **4B-2**. Specifically, there are 27 features of AA8 and 49 features of AA11 within the first 100 features in the MaxRel feature list (see Table S4). It shows that the AA8 and AA11 are most essential for predicting the hydroxylysine using AAindex, PSSM conservation and disorder features.

**Figure 5:**
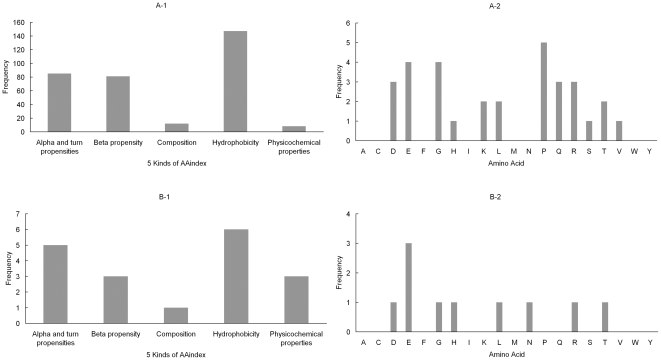
Distribution of the 5 feature clusters of the AAindex and distribution of conservation of 20 amino acids in the feature set A and B for hydroxylysine dataset.

**Figure 5** shows the distribution of the 5 feature clusters of the AAindex and the distribution of conservation of 20 amino acids in the two feature sets. **Figure 5A** **-1** and **Figure 5B**-1**** show that all the 5 kinds of AAindex exert influence on the hydroxylation of lysine residue. Alpha and turn propensities, beta propensity and physicochemical properties are closely related to the hydroxylation among the AAindex (see **Figure 5A**-1****). Like the proline hydroxylation, Alpha and turn propensities and hydrophobicity are useful in identifying hydroxylated proline residues. Structure of type I collagen central triple helical domains show that lysine hydroxylation is important to determine the pattern process and of cross-linking collagen [9], [62]. Forming such kind of structure appears close related to alpha and turn propensities and hydrophobicity in sequence. In **Figure 5A**-2****, the distinct features are the mutations of the glutamic acid, glycine and proline. However, the conservation of glycine and proline are not marked in **Figure 5B**-2****. This may be because that the high correlation exists between the two mutations and the other mutations, especially the mutation of glutamic acid. Among the 10 kinds of mutations in the feature set B (**Figure 5B**-2****), the mutation of glutamic acid is more important in the classification of hydroxylation sites and non-hydroxylation sites than others.

In summary, proline hydroxylation and lysine hydroxylation share many common analysis results according to the above discussion. Evolution information is of vital importance for the hydroxylation of proline and lysine residues. Structural disorder shows little relation to the hydroxylation. As the nearest neighbor of the middle site in the peptides, AA8 tends to have the great effect on the hydroxylation of proline and lysine residues. Alpha and turn propensities and hydrophobicity are extremely important in identifying hydroxyproline and hydroxylysine. Up until now, the mechanism of protein hydroxylation is not clearly known. Therefore, the results in this study may provide clues for the biologists to design the experiments and for bioinformatists to develop annotation tools.

### Conclusion

In this study, we proposed an annotation tool to identify the hydroxyproline and hydroxylysine. The relationship between three kinds of amino acid features and protein hydroxylation were investigated. Feature analysis indicates that physicochemical properties and biochemical properties and evolution information of amino acids play important roles in identifying the protein hydroxylation sites, while structural disorder had little relation to protein hydroxylation. Position specific distribution of the peptides suggested that AA8 exert a great effect on the hydroxylation of proline and lysine. The hydroxylation sites predicted by our method may serve as the potential hydroxylation sites for the biologists to do further experiments. The software is available upon request.

## Supporting Information

Table S1 2034 peptides extracted from hydroxyproline dataset.
(DOC)

Table S2 324 peptides extracted from hydroxylysine dataset.
(DOC)

Table S3 The MaxRel feature list and the mRMR feature list for hydroxyproline dataset.
(DOC)

Table S4 The MaxRel feature list and the mRMR feature list for hydroxylysine dataset.
(DOC)

Table S5 Performance of 500 NNA predictors for hydroxyproline dataset.
(DOC)

Table S6 Performance of 500 NNA predictors for hydroxylysine dataset.
(DOC)
