# Supplementary material for: Prediction and Analysis of Protein Hydroxyproline and Hydroxylysine
Source: PLoS One. 2010 Dec 31;5(12):e15917. doi: 10.1371/journal.pone.0015917 (PMC3013141; doi:10.1371/journal.pone.0015917)
Supplement: Table S1 — 2034 peptides extracted from hydroxyproline dataset. (DOC) [file pone.0015917.s001.doc]

**Table S1. 2034 peptides extracted from hydroxyproline dataset**

Peptides with 13 residues that consisted of a proline residue, 6 residues upstream and 6 residues downstream of the proline residue were extracted from the protein sequences in hydroxyproline dataset. If the peptides exceeded the boundary of protein sequence, we inserted the non-existent residues coded by ‘-’ to make up of the peptides with 13 residues. Totally, there were 4081 peptides consisting of 678 hydroxylated proline residues and 3403 non-hydroxylated proline residues with experimental verification in the dataset. Then the 678 peptides with hydroxylated proline residues were assigned as positive samples, while 1356 peptides that were randomly selected from the 3403 peptides containing non-hydroxylated proline residues were assigned as negative samples. All the 2034 samples are listed as follows.

| Uniprot-AC | Position | Peptide | Class |
| --- | --- | --- | --- |
| P0C8V6 | 57 | RDECYPPGTFCGI | Positive |
| P0C8V6 | 65 | TFCGIKPGLCCSE | Positive |
| O97939 | 547 | VYTEGIPSPAKEH | Positive |
| Q7XAD0 | 51 | GNYGRTPYKTPPP | Positive |
| Q7XAD0 | 55 | RTPYKTPPPPTSS | Positive |
| Q7XAD0 | 56 | TPYKTPPPPTSSS | Positive |
| Q7XAD0 | 57 | PYKTPPPPTSSSP | Positive |
| Q7XAD0 | 58 | YKTPPPPTSSSPT | Positive |
| Q7XAD0 | 63 | PPTSSSPTHQEIV | Positive |
| Q7XAD0 | 79 | HDSVLPPPSPKTD | Positive |
| Q7XAD0 | 80 | DSVLPPPSPKTDP | Positive |
| Q7XAD0 | 82 | VLPPPSPKTDPII | Positive |
| Q7XAD0 | 119 | HDYVASPPPPKPQ | Positive |
| Q7XAD0 | 120 | DYVASPPPPKPQD | Positive |
| Q7XAD0 | 121 | YVASPPPPKPQDE | Positive |
| Q7XAD0 | 122 | VASPPPPKPQDEQ | Positive |
| P23805 | 78 | AGRAGRPGWVGPI | Positive |
| P23805 | 96 | NGFVGEPGPKGDT | Positive |
| P23805 | 108 | TGPRGPPGMPGPA | Positive |
| P23805 | 111 | RGPPGMPGPAGRE | Positive |
| P23805 | 129 | QGSMGPPGTPGPK | Positive |
| P23805 | 132 | MGPPGTPGPKGET | Positive |
| P23805 | 147 | KGGVGAPGIQGFP | Positive |
| P23805 | 153 | PGIQGFPGPSGLK | Positive |
| P23805 | 171 | PGETGAPGRAGVT | Positive |
| P23805 | 195 | SGARGPPGLKGDR | Positive |
| P69747 | 55 | RGCCSDPRCNYDH | Positive |
| P01522 | 49 | STRCKSPGSSCSP | Positive |
| P01522 | 55 | PGSSCSPTSYNCC | Positive |
| P01522 | 66 | CCRSCNPYTKRCY | Positive |
| O75636 | 50 | PSCPGAPGSPGEK | Positive |
| O75636 | 53 | PGAPGSPGEKGAP | Positive |
| O75636 | 59 | PGEKGAPGPQGPP | Positive |
| O75636 | 65 | PGPQGPPGPPGKM | Positive |
| O75636 | 68 | QGPPGPPGKMGPK | Positive |
| O75636 | 77 | MGPKGEPGDPVNL | Positive |
| O82337 | 31 | GAQSLAPAPAPTS | Positive |
| O82337 | 33 | QSLAPAPAPTSDG | Positive |
| O82337 | 35 | LAPAPAPTSDGTS | Positive |
| P05997 | 290 | PGARGFPGAPGLP | Positive |
| P05997 | 293 | RGFPGAPGLPGLK | Positive |
| P05997 | 296 | PGAPGLPGLKGHR | Positive |
| P05997 | 611 | IGIRGQPGSMGLP | Positive |
| P05997 | 617 | PGSMGLPGPKGSS | Positive |
| P01525 | 10 | TWGAAYPACENNC | Positive |
| Q557E4 | 143 | IKNDFTPEEEEQI | Positive |
| P0C8W0 | 53 | EKRGCDPTDGCQT | Positive |
| P0C8W0 | 68 | CETDTGPCCCKPN | Positive |
| P0C8W0 | 93 | CSCSGQPSDCPV- | Positive |
| P0C8W0 | 97 | GQPSDCPV----- | Positive |
| Q9LJD9 | 32 | AAQTEAPAPSPTS | Positive |
| Q9LJD9 | 34 | QTEAPAPSPTSDA | Positive |
| Q9LJD9 | 36 | EAPAPSPTSDAAM | Positive |
| P56529 | 52 | LLERRHPPCCMYG | Positive |
| P56529 | 53 | LERRHPPCCMYGR | Positive |
| P56529 | 64 | GRCRRYPGCSSAS | Positive |
| Q9Y2N7 | 492 | DLEMLAPYISMDD | Positive |
| Q9SJY7 | 24 | SCLAQAPAPAPTT | Positive |
| Q9SJY7 | 26 | LAQAPAPAPTTVT | Positive |
| Q9SJY7 | 28 | QAPAPAPTTVTPP | Positive |
| Q9SJY7 | 34 | PTTVTPPPTALPP | Positive |
| Q9SJY7 | 35 | TTVTPPPTALPPV | Positive |
| P84900 | 54 | ESPDRPPGFSPFR | Positive |
| P08252 | 67 | SQCPGGPTPTPPT | Positive |
| P08252 | 69 | CPGGPTPTPPTPP | Positive |
| P08252 | 71 | GGPTPTPPTPPGG | Positive |
| P08252 | 72 | GPTPTPPTPPGGG | Positive |
| P08252 | 74 | TPTPPTPPGGGDL | Positive |
| P08252 | 75 | PTPPTPPGGGDLG | Positive |
| Q16665 | 402 | ALTLLAPAAGDTI | Positive |
| Q16665 | 564 | DLEMLAPYIPMDD | Positive |
| P19999 | 61 | RGLQGPPGKLGPP | Positive |
| P19999 | 67 | PGKLGPPGSVGAP | Positive |
| P19999 | 73 | PGSVGAPGSQGPK | Positive |
| P84349 | 60 | YQWLGAPVPYPDP | Positive |
| P08125 | 453 | IGPPGMPGAPGAK | Positive |
| P08125 | 456 | PGMPGAPGAKGEA | Positive |
| P81755 | 63 | WCCTAAPLTGR-- | Positive |
| P69929 | 39 | PNVAVPPCGDCYQ | Positive |
| P69929 | 73 | PNVAVPPCGDCYQ | Positive |
| P69929 | 107 | PNVAVPPCGDCYQ | Positive |
| P69929 | 141 | PNVAVPPCGDCYQ | Positive |
| P69929 | 175 | PNVAVPPCGDCYQ | Positive |
| P69929 | 209 | PNVAVPPCGDCYQ | Positive |
| Q9LVC0 | 32 | VAAVDAPAPSPTS | Positive |
| Q9LVC0 | 34 | AVDAPAPSPTSDA | Positive |
| Q9LVC0 | 36 | DAPAPSPTSDASS | Positive |
| Q9XZK5 | 65 | TFCGIHPGLCCSE | Positive |
| P58846 | 62 | TCFGCTPCCG--- | Positive |
| P52285 | 143 | IKNDFTPEEEEQI | Positive |
| Q60994 | 94 | MTGAEGPRGFPGT | Positive |
| P0C8V5 | 57 | RDECFSPGTFCGI | Positive |
| P0C8V5 | 65 | TFCGIKPGLCCSA | Positive |
| P01523 | 56 | RRDCCTPPKKCKD | Positive |
| P01523 | 57 | RDCCTPPKKCKDR | Positive |
| P01523 | 67 | KDRQCKPQRCCAG | Positive |
| Q9GQV7 | 26 | CEDQRPPSLKTRF | Positive |
| Q9GQV7 | 59 | RSAQRPPSLKTRF | Positive |
| Q9GQV7 | 83 | RSAQRPPSLKTRF | Positive |
| Q3Y5Z3 | 39 | GWMAGIPGHPGHN | Positive |
| Q3Y5Z3 | 42 | AGIPGHPGHNGTP | Positive |
| Q3Y5Z3 | 48 | PGHNGTPGRDGRD | Positive |
| Q3Y5Z3 | 86 | ITGIEGPRGFPGT | Positive |
| Q05707 | 1467 | LGPAGPPGGPGLR | Positive |
| Q05707 | 1470 | AGPPGGPGLRGPK | Positive |
| Q05707 | 1482 | KGQQGEPGPKGPD | Positive |
| Q05707 | 1497 | RGEIGLPGPQGPP | Positive |
| Q05707 | 1503 | PGPQGPPGPQGPS | Positive |
| Q05707 | 1517 | LSIQGMPGMPGEK | Positive |
| Q05707 | 1520 | QGMPGMPGEKGEK | Positive |
| Q05707 | 1532 | KGDTGLPGPQGIP | Positive |
| Q05707 | 1538 | PGPQGIPGGVGSP | Positive |
| Q05707 | 1544 | PGGVGSPGRDGSP | Positive |
| Q05707 | 1550 | PGRDGSPGQRGLP | Positive |
| Q05707 | 1556 | PGQRGLPGKDGSS | Positive |
| Q05707 | 1565 | DGSSGPPGPPGPI | Positive |
| Q05707 | 1568 | SGPPGPPGPIGIP | Positive |
| Q05707 | 1574 | PGPIGIPGTPGVP | Positive |
| Q05707 | 1577 | IGIPGTPGVPGIT | Positive |
| Q05707 | 1580 | PGTPGVPGITGSM | Positive |
| Q05707 | 1595 | QGALGPPGVPGAK | Positive |
| Q05707 | 1598 | LGPPGVPGAKGER | Positive |
| Q05707 | 1643 | AILNQIPSHSSSI | Positive |
| Q05707 | 1656 | RTVQGPPGEPGRP | Positive |
| Q05707 | 1659 | QGPPGEPGRPGSP | Positive |
| Q05707 | 1662 | PGEPGRPGSPGAP | Positive |
| Q05707 | 1665 | PGRPGSPGAPGEQ | Positive |
| Q05707 | 1668 | PGSPGAPGEQGPP | Positive |
| Q05707 | 1674 | PGEQGPPGTPGFP | Positive |
| Q05707 | 1677 | QGPPGTPGFPGNA | Positive |
| Q05707 | 1680 | PGTPGFPGNAGVP | Positive |
| Q05707 | 1686 | PGNAGVPGTPGER | Positive |
| Q05707 | 1689 | AGVPGTPGERGLT | Positive |
| Q05707 | 1704 | KGEKGNPGVGTQG | Positive |
| Q05707 | 1715 | QGPRGPPGPAGPS | Positive |
| Q05707 | 1726 | PSGESRPGSPGPP | Positive |
| Q05707 | 1729 | ESRPGSPGPPGSP | Positive |
| Q05707 | 1732 | PGSPGPPGSPGPR | Positive |
| Q05707 | 1735 | PGPPGSPGPRGPP | Positive |
| Q05707 | 1741 | PGPRGPPGHLGVP | Positive |
| Q05707 | 1747 | PGHLGVPGPQGPS | Positive |
| Q05707 | 1756 | QGPSGQPGYCDPS | Positive |
| P58787 | 57 | LRRFGCPWQPWCG | Positive |
| P56633 | 49 | STRCRIPNQKCFQ | Positive |
| P02820 | 60 | DHWLGAPAPYPDP | Positive |
| P86289 | 6 | #NAME? | Positive |
| P86289 | 12 | PGIAGAPGFPGAR | Positive |
| P86289 | 15 | AGAPGFPGARGPS | Positive |
| P86289 | 30 | QGPSGAPGPKGVQ | Positive |
| P86289 | 39 | KGVQGPPGPQGPR | Positive |
| P86289 | 54 | TGPIGPPGPAGAP | Positive |
| P86289 | 60 | PGPAGAPGDKGEA | Positive |
| P86289 | 84 | RGSAGPPGATGFP | Positive |
| P86289 | 90 | PGATGFPGAAGRG | Positive |
| P86289 | 107 | AGPAGPPGPAGAR | Positive |
| P0C1X1 | 40 | VVHERAPELVVTA | Positive |
| P0C1X1 | 55 | NCCGYNPMTICPP | Positive |
| P0C1X1 | 60 | NPMTICPPCMCTY | Positive |
| P0C1X1 | 61 | PMTICPPCMCTYS | Positive |
| P0C1X1 | 69 | MCTYSCPPKRKPG | Positive |
| P0C1X1 | 70 | CTYSCPPKRKPGR | Positive |
| P0C1X1 | 74 | CPPKRKPGRRND- | Positive |
| P05623 | 28 | WPWNRKPTKFPIP | Positive |
| P05623 | 32 | RKPTKFPIPSPNP | Positive |
| P05623 | 34 | PTKFPIPSPNPRD | Positive |
| P05623 | 38 | PIPSPNPRDKWCR | Positive |
| B2KPN7 | 64 | YHAHPKPNSFWTL | Positive |
| Q9LYF6 | 27 | SAQSEAPAPSPTS | Positive |
| Q9LYF6 | 29 | QSEAPAPSPTSGS | Positive |
| Q9LYF6 | 31 | EAPAPSPTSGSSA | Positive |
| P0C1N1 | 64 | CGLGCHPCCG--- | Positive |
| P01526 | 10 | TWGGSYPACENNC | Positive |
| P0C1N5 | 55 | AVRDCCPLPACPF | Positive |
| P0C1N5 | 65 | CPFGCNPCCG--- | Positive |
| P08661 | 69 | RGLQGPPGKVGPA | Positive |
| Q941C7 | 63 | KHDPGVPPSATGQ | Positive |
| Q941C7 | 64 | HDPGVPPSATGQR | Positive |
| P08123 | 420 | AGVMGPPGSRGAS | Positive |
| P08123 | 441 | NGDAGRPGEPGLM | Positive |
| P08123 | 444 | AGRPGEPGLMGPR | Positive |
| Q9U3Z3 | 53 | WGGCGHPCRHPGK | Positive |
| P02746 | 33 | LSCTGPPAIPGIP | Positive |
| P02746 | 36 | TGPPAIPGIPGIP | Positive |
| P02746 | 39 | PAIPGIPGIPGTP | Positive |
| P02746 | 42 | PGIPGIPGTPGPD | Positive |
| P02746 | 51 | PGPDGQPGTPGIK | Positive |
| P02746 | 54 | DGQPGTPGIKGEK | Positive |
| P02746 | 63 | KGEKGLPGLAGDH | Positive |
| P02746 | 81 | KGDPGIPGNPGKV | Positive |
| P02746 | 84 | PGIPGNPGKVGPK | Positive |
| P02746 | 99 | MGPKGGPGAPGAP | Positive |
| P02746 | 102 | KGGPGAPGAPGPK | Positive |
| P02746 | 105 | PGAPGAPGPKGES | Positive |
| Q25460 | 89 | PTYKPKPSYPPTY | Positive |
| Q25460 | 92 | KPKPSYPPTYKSK | Positive |
| Q25460 | 93 | PKPSYPPTYKSKP | Positive |
| Q25460 | 115 | PTYKAKPSYPSSY | Positive |
| Q25460 | 118 | KAKPSYPSSYKPK | Positive |
| Q25460 | 145 | PTYKPKPSYPPTY | Positive |
| Q25460 | 148 | KPKPSYPPTYKPK | Positive |
| Q25460 | 149 | PKPSYPPTYKPKP | Positive |
| Q25460 | 155 | PTYKPKPSYPPSY | Positive |
| Q25460 | 158 | KPKPSYPPSYKTK | Positive |
| Q25460 | 159 | PKPSYPPSYKTKK | Positive |
| Q25460 | 175 | SSYKAKPSYPPTY | Positive |
| Q25460 | 178 | KAKPSYPPTYKAK | Positive |
| Q25460 | 179 | AKPSYPPTYKAKP | Positive |
| Q25460 | 185 | PTYKAKPSYPPTY | Positive |
| Q25460 | 188 | KAKPSYPPTYKAK | Positive |
| Q25460 | 189 | AKPSYPPTYKAKP | Positive |
| Q25460 | 195 | PTYKAKPSYPPTY | Positive |
| Q25460 | 198 | KAKPSYPPTYKAK | Positive |
| Q25460 | 199 | AKPSYPPTYKAKP | Positive |
| Q25460 | 211 | PTYKAKPTYPSTY | Positive |
| Q25460 | 214 | KAKPTYPSTYKAK | Positive |
| Q25460 | 221 | STYKAKPSYPPTY | Positive |
| Q25460 | 224 | KAKPSYPPTYKAK | Positive |
| Q25460 | 225 | AKPSYPPTYKAKP | Positive |
| Q25460 | 237 | PTYKAKPSYPPTY | Positive |
| Q25460 | 240 | KAKPSYPPTYKAK | Positive |
| Q25460 | 241 | AKPSYPPTYKAKP | Positive |
| Q25460 | 247 | PTYKAKPSYPPTY | Positive |
| Q25460 | 250 | KAKPSYPPTYKAK | Positive |
| Q25460 | 251 | AKPSYPPTYKAKP | Positive |
| Q25460 | 257 | PTYKAKPSYPPTY | Positive |
| Q25460 | 260 | KAKPSYPPTYKAK | Positive |
| Q25460 | 261 | AKPSYPPTYKAKP | Positive |
| Q25460 | 285 | PTYKAKPSYPPTY | Positive |
| Q25460 | 288 | KAKPSYPPTYKAK | Positive |
| Q25460 | 289 | AKPSYPPTYKAKP | Positive |
| Q25460 | 295 | PTYKAKPSYPPTY | Positive |
| Q25460 | 298 | KAKPSYPPTYKAK | Positive |
| Q25460 | 299 | AKPSYPPTYKAKP | Positive |
| Q25460 | 305 | PTYKAKPSYPPTY | Positive |
| Q25460 | 308 | KAKPSYPPTYKAK | Positive |
| Q25460 | 309 | AKPSYPPTYKAKP | Positive |
| Q25460 | 315 | PTYKAKPSYPPTY | Positive |
| Q25460 | 318 | KAKPSYPPTYKAK | Positive |
| Q25460 | 319 | AKPSYPPTYKAKP | Positive |
| Q25460 | 325 | PTYKAKPSYPPTY | Positive |
| Q25460 | 328 | KAKPSYPPTYKAK | Positive |
| Q25460 | 329 | AKPSYPPTYKAKP | Positive |
| Q25460 | 335 | PTYKAKPSYPPTY | Positive |
| Q25460 | 338 | KAKPSYPPTYKAK | Positive |
| Q25460 | 339 | AKPSYPPTYKAKP | Positive |
| Q25460 | 351 | PTYKAKPTYPSTY | Positive |
| Q25460 | 354 | KAKPTYPSTYKAK | Positive |
| Q25460 | 361 | STYKAKPSYPPTY | Positive |
| Q25460 | 364 | KAKPSYPPTYKAK | Positive |
| Q25460 | 365 | AKPSYPPTYKAKP | Positive |
| Q25460 | 371 | PTYKAKPSYPPTY | Positive |
| Q25460 | 374 | KAKPSYPPTYKAK | Positive |
| Q25460 | 375 | AKPSYPPTYKAKP | Positive |
| Q25460 | 387 | PTYKAKPSYPPTY | Positive |
| Q25460 | 390 | KAKPSYPPTYKAK | Positive |
| Q25460 | 391 | AKPSYPPTYKAKP | Positive |
| Q25460 | 397 | PTYKAKPSYPPTY | Positive |
| Q25460 | 400 | KAKPSYPPTYKAK | Positive |
| Q25460 | 401 | AKPSYPPTYKAKP | Positive |
| Q25460 | 407 | PTYKAKPSYPPTY | Positive |
| Q25460 | 410 | KAKPSYPPTYKAK | Positive |
| Q25460 | 411 | AKPSYPPTYKAKP | Positive |
| Q25460 | 423 | PTYKAKPTYPSTY | Positive |
| Q25460 | 426 | KAKPTYPSTYKAK | Positive |
| Q25460 | 433 | STYKAKPSYPPSY | Positive |
| Q25460 | 436 | KAKPSYPPSYKAK | Positive |
| Q25460 | 437 | AKPSYPPSYKAKP | Positive |
| Q25460 | 443 | PSYKAKPSYPPTY | Positive |
| Q25460 | 446 | KAKPSYPPTYKAK | Positive |
| Q25460 | 447 | AKPSYPPTYKAKP | Positive |
| Q25460 | 459 | PTYKAKPTYPSTY | Positive |
| Q25460 | 462 | KAKPTYPSTYKAK | Positive |
| Q25460 | 469 | STYKAKPSYPASY | Positive |
| Q25460 | 472 | KAKPSYPASYKAK | Positive |
| Q25460 | 479 | ASYKAKPSYPPTY | Positive |
| Q25460 | 482 | KAKPSYPPTYKSK | Positive |
| Q25460 | 483 | AKPSYPPTYKSKS | Positive |
| Q25460 | 519 | PTYKPKPSYPPSY | Positive |
| Q25460 | 522 | KPKPSYPPSYKPK | Positive |
| Q25460 | 523 | PKPSYPPSYKPKT | Positive |
| Q25460 | 542 | KPKISYPPTYKAK | Positive |
| Q25460 | 543 | PKISYPPTYKAKP | Positive |
| Q25460 | 549 | PTYKAKPSYPATY | Positive |
| Q25460 | 552 | KAKPSYPATYKAK | Positive |
| Q25460 | 559 | ATYKAKPSYPPTY | Positive |
| Q25460 | 562 | KAKPSYPPTYKAK | Positive |
| Q25460 | 563 | AKPSYPPTYKAKP | Positive |
| Q25460 | 569 | PTYKAKPSYPPTY | Positive |
| Q25460 | 572 | KAKPSYPPTYKAK | Positive |
| Q25460 | 573 | AKPSYPPTYKAKP | Positive |
| Q25460 | 579 | PTYKAKPSYPPTY | Positive |
| Q25460 | 582 | KAKPSYPPTYKAK | Positive |
| Q25460 | 583 | AKPSYPPTYKAKP | Positive |
| Q25460 | 595 | PSYKAKPTYPSTY | Positive |
| Q25460 | 598 | KAKPTYPSTYKAK | Positive |
| Q25460 | 605 | STYKAKPSYPPTY | Positive |
| Q25460 | 608 | KAKPSYPPTYKAK | Positive |
| Q25460 | 609 | AKPSYPPTYKAKP | Positive |
| Q25460 | 615 | PTYKAKPSYPPTY | Positive |
| Q25460 | 618 | KAKPSYPPTYKAK | Positive |
| Q25460 | 619 | AKPSYPPTYKAKP | Positive |
| Q25460 | 625 | PTYKAKPSYPPTY | Positive |
| Q25460 | 628 | KAKPSYPPTYKAK | Positive |
| Q25460 | 629 | AKPSYPPTYKAKP | Positive |
| Q25460 | 635 | PTYKAKPTYPSTY | Positive |
| Q25460 | 638 | KAKPTYPSTYKAK | Positive |
| Q25460 | 645 | STYKAKPSYPPTY | Positive |
| Q25460 | 648 | KAKPSYPPTYKPK | Positive |
| Q25460 | 649 | AKPSYPPTYKPKI | Positive |
| Q25460 | 658 | KPKISYPPTYKAK | Positive |
| Q25460 | 659 | PKISYPPTYKAKP | Positive |
| Q25460 | 665 | PTYKAKPSYPPTY | Positive |
| Q25460 | 668 | KAKPSYPPTYKAK | Positive |
| Q25460 | 669 | AKPSYPPTYKAKP | Positive |
| Q25460 | 675 | PTYKAKPSYPPTY | Positive |
| Q25460 | 678 | KAKPSYPPTYKAK | Positive |
| Q25460 | 679 | AKPSYPPTYKAKP | Positive |
| Q25460 | 701 | STYKAKPSYPPTY | Positive |
| Q25460 | 704 | KAKPSYPPTYKAK | Positive |
| Q25460 | 705 | AKPSYPPTYKAKP | Positive |
| Q25460 | 711 | PTYKAKPSYPPTY | Positive |
| Q25460 | 714 | KAKPSYPPTYKAK | Positive |
| Q25460 | 715 | AKPSYPPTYKAKP | Positive |
| Q25460 | 721 | PTYKAKPSYPPTY | Positive |
| Q25460 | 724 | KAKPSYPPTYKAK | Positive |
| Q25460 | 725 | AKPSYPPTYKAKP | Positive |
| Q25460 | 737 | PTYKAKPTYPSTY | Positive |
| Q25460 | 740 | KAKPTYPSTYKAK | Positive |
| Q25460 | 753 | PTYKAKPTYPPTY | Positive |
| Q25460 | 756 | KAKPTYPPTYKAK | Positive |
| Q25460 | 757 | AKPTYPPTYKAKP | Positive |
| Q25460 | 763 | PTYKAKPSYPPTY | Positive |
| Q25460 | 766 | KAKPSYPPTYKPK | Positive |
| Q25460 | 767 | AKPSYPPTYKPKP | Positive |
| Q25460 | 773 | PTYKPKPSYPPTY | Positive |
| Q25460 | 776 | KPKPSYPPTYKSK | Positive |
| Q25460 | 777 | PKPSYPPTYKSKS | Positive |
| Q25460 | 813 | PTYKPKPSYPPSY | Positive |
| Q25460 | 816 | KPKPSYPPSYKPK | Positive |
| Q25460 | 817 | PKPSYPPSYKPKI | Positive |
| Q25460 | 833 | STYKLKPSYPPTY | Positive |
| Q25460 | 836 | KLKPSYPPTYKSK | Positive |
| Q25460 | 837 | LKPSYPPTYKSKT | Positive |
| Q25460 | 866 | KAKTSYPPAYKPT | Positive |
| Q25460 | 867 | AKTSYPPAYKPTN | Positive |
| P11226 | 47 | PGINGFPGKDGRD | Positive |
| P11226 | 73 | RGLQGPPGKLGPP | Positive |
| P11226 | 79 | PGKLGPPGNPGPS | Positive |
| P11226 | 82 | LGPPGNPGPSGSP | Positive |
| P11226 | 88 | PGPSGSPGPKGQK | Positive |
| P35248 | 77 | MGLSGLPGPRGPV | Positive |
| P35248 | 95 | NGSAGEPGPKGER | Positive |
| P35248 | 170 | KGERGAPGEQGAP | Positive |
| P35248 | 176 | PGEQGAPGNAGAA | Positive |
| P69928 | 29 | VLKRGFPCRCDSD | Positive |
| P30754 | 21 | IGPRGPPGPPGSP | Positive |
| P30754 | 24 | RGPPGPPGSPGQQ | Positive |
| P30754 | 27 | PGPPGSPGQQGYQ | Positive |
| P30754 | 39 | QGLRGEPGDSGPM | Positive |
| P30754 | 53 | PIGKRGPPGPAGI | Positive |
| P30754 | 54 | IGKRGPPGPAGIA | Positive |
| P30754 | 72 | DGRDGEPGPRGGI | Positive |
| P30754 | 90 | RGAGGMPGMPGPK | Positive |
| P30754 | 93 | GGMPGMPGPKGHR | Positive |
| P30754 | 123 | QGPDGGPGPAGPS | Positive |
| P30754 | 128 | GPGPAGPSGPIGP | Positive |
| P30754 | 150 | DGKSGLPGLRGVD | Positive |
| P30754 | 161 | VDGLAGPPGPPGP | Positive |
| P30754 | 162 | DGLAGPPGPPGPI | Positive |
| P30754 | 164 | LAGPPGPPGPIGS | Positive |
| P30754 | 165 | AGPPGPPGPIGST | Positive |
| P30754 | 174 | IGSTGSPGFPGTP | Positive |
| P30754 | 177 | TGSPGFPGTPGSK | Positive |
| P30754 | 180 | PGFPGTPGSKGDR | Positive |
| P30754 | 207 | VGLSGQPGVAGEN | Positive |
| P30754 | 216 | AGENGHPGMPGMD | Positive |
| P30754 | 219 | NGHPGMPGMDGAN | Positive |
| P30754 | 228 | DGANGEPGASGES | Positive |
| P30754 | 237 | SGESGLPGPSGFP | Positive |
| P30754 | 243 | PGPSGFPGPRGMP | Positive |
| P30754 | 249 | PGPRGMPGTAGSP | Positive |
| P30754 | 255 | PGTAGSPGQAGAK | Positive |
| P30754 | 273 | TGEQGRPGAPGVK | Positive |
| P30754 | 276 | QGRPGAPGVKGSS | Positive |
| P30754 | 285 | KGSSGPPGDVGAP | Positive |
| P30754 | 291 | PGDVGAPGHAGEA | Positive |
| P30754 | 303 | AGKRGSPGSPGPA | Positive |
| P30754 | 306 | RGSPGSPGPAGSP | Positive |
| P30754 | 312 | PGPAGSPGPQGDR | Positive |
| P30754 | 321 | QGDRGLPGSRGLP | Positive |
| P30754 | 327 | PGSRGLPGMTGAS | Positive |
| P30754 | 339 | SGAMGIPGEKGPS | Positive |
| P30754 | 348 | KGPSGEPGAKGPT | Positive |
| P30754 | 366 | QGNQGTPGIAGLP | Positive |
| P30754 | 372 | PGIAGLPGNPGSD | Positive |
| P30754 | 375 | AGLPGNPGSDGRP | Positive |
| P30754 | 381 | PGSDGRPGKDGRP | Positive |
| P30754 | 387 | PGKDGRPGIRGKD | Positive |
| P30754 | 416 | LQGRAGPPGARGE | Positive |
| P30754 | 417 | QGRAGPPGARGEP | Positive |
| P30754 | 423 | PGARGEPGKNGAP | Positive |
| P30754 | 429 | PGKNGAPGEPGAH | Positive |
| P30754 | 432 | NGAPGEPGAHGEQ | Positive |
| P30754 | 453 | TGAAGPPGAAGPT | Positive |
| P30754 | 465 | TGARGPPGPRGQQ | Positive |
| P30754 | 483 | AGAQGTPGEAGKT | Positive |
| P30754 | 500 | AVGATGPSGPAGP | Positive |
| P30754 | 503 | ATGPSGPAGPGGE | Positive |
| P30754 | 506 | PSGPAGPGGERGA | Positive |
| P30754 | 513 | GGERGAPGDRGNV | Positive |
| P30754 | 525 | VGPRGMPGERGAT | Positive |
| P30754 | 533 | ERGATGPAGPTGS | Positive |
| P30754 | 536 | ATGPAGPTGSPGV | Positive |
| P30754 | 540 | AGPTGSPGVAGAK | Positive |
| P30754 | 551 | AKGQGGPPGPAGL | Positive |
| P30754 | 552 | KGQGGPPGPAGLV | Positive |
| P30754 | 561 | AGLVGLPGERGPK | Positive |
| P30754 | 603 | RGENGLPGPSGLA | Positive |
| P30754 | 621 | RGDMGSPGERGSP | Positive |
| P30754 | 627 | PGERGSPGPAGER | Positive |
| P30754 | 645 | QGIQGQPGPPGDA | Positive |
| P30754 | 647 | IQGQPGPPGDAGP | Positive |
| P30754 | 648 | QGQPGPPGDAGPA | Positive |
| P30754 | 663 | KGDIGFPGERGTR | Positive |
| P30754 | 708 | QGEIGLPGSPGQP | Positive |
| P30754 | 711 | IGLPGSPGQPGLP | Positive |
| P30754 | 714 | PGSPGQPGLPGPS | Positive |
| P30754 | 717 | PGQPGLPGPSGQP | Positive |
| P30754 | 723 | PGPSGQPGPSGPA | Positive |
| P30754 | 744 | KGARGSPGLVGKQ | Positive |
| P30754 | 759 | RGSDGEPGRDGTK | Positive |
| P30754 | 773 | ERGEDGPPGVSGP | Positive |
| P30754 | 774 | RGEDGPPGVSGPT | Positive |
| P30754 | 783 | SGPTGAPGQQGER | Positive |
| P30754 | 792 | QGERGMPGMVGLR | Positive |
| P30754 | 815 | MKGDGGPPGPSGD | Positive |
| P30754 | 816 | KGDGGPPGPSGDR | Positive |
| P30754 | 843 | SGQAGAPGQEGAP | Positive |
| P30754 | 849 | PGQEGAPGKDGLP | Positive |
| P30754 | 855 | PGKDGLPGLAGRP | Positive |
| P30754 | 861 | PGLAGRPGERGEP | Positive |
| P30754 | 867 | PGERGEPGVAGRA | Positive |
| P30754 | 888 | MGQRGLPGAAGPP | Positive |
| P30754 | 894 | PGAAGPPGDRGER | Positive |
| P30754 | 903 | RGERGEPGGQGVQ | Positive |
| P30754 | 915 | QGPVGAPGSQGPA | Positive |
| P30754 | 945 | KGWTGLPGLQGLQ | Positive |
| P30754 | 954 | QGLQGTPGHSGES | Positive |
| P30754 | 963 | SGESGPPGAPGPR | Positive |
| P30754 | 966 | SGPPGAPGPRGAR | Positive |
| P30754 | 984 | RGSQGPPGKDGQP | Positive |
| P30754 | 990 | PGKDGQPGPSGRV | Positive |
| P30754 | 1010 | DDGRSGPPGPPGP | Positive |
| P30754 | 1011 | DGRSGPPGPPGPP | Positive |
| P30754 | 1013 | RSGPPGPPGPPGP | Positive |
| P30754 | 1014 | SGPPGPPGPPGPP | Positive |
| P30754 | 1016 | PPGPPGPPGPPGN | Positive |
| P30754 | 1017 | PGPPGPPGPPGNS | Positive |
| P30754 | 1019 | PPGPPGPPGNSDY | Positive |
| P30754 | 1020 | PGPPGPPGNSDYG | Positive |
| P04925 | 44 | YPGQGSPGGNRYP | Positive |
| P08427 | 30 | DVCAGSPGIPGAP | Positive |
| P08427 | 33 | AGSPGIPGAPGNH | Positive |
| P08427 | 36 | PGIPGAPGNHGLP | Positive |
| P08427 | 42 | PGNHGLPGRDGRD | Positive |
| P08427 | 54 | DGVKGDPGPPGPM | Positive |
| P08427 | 57 | KGDPGPPGPMGPP | Positive |
| P08427 | 63 | PGPMGPPGGMPGL | Positive |
| P08427 | 67 | GPPGGMPGLPGRD | Positive |
| P08427 | 70 | GGMPGLPGRDGLP | Positive |
| P08427 | 76 | PGRDGLPGAPGAP | Positive |
| Q0VKG8 | 56 | ERRDRPPSWIPK- | Positive |
| P42849 | 113 | AGGGATPGA---- | Positive |
| Q9UKV8 | 700 | LEKDYQPGITFIV | Positive |
| P02745 | 39 | KGEAGRPGRRGRP | Positive |
| P02745 | 45 | PGRRGRPGLKGEQ | Positive |
| P02745 | 54 | KGEQGEPGAPGIR | Positive |
| P02745 | 57 | QGEPGAPGIRTGI | Positive |
| P02745 | 73 | KGDQGEPGPSGNP | Positive |
| P02745 | 85 | PGKVGYPGPSGPL | Positive |
| P02745 | 97 | LGARGIPGIKGTK | Positive |
| P58925 | 57 | RLCCGFPKSCRSR | Positive |
| P58925 | 67 | RSRQCKPHRCCGR | Positive |
| Q9XF04 | 73 | EELRTVPSGPDPL | Positive |
| Q9XF04 | 76 | RTVPSGPDPLHHH | Positive |
| P58808 | 60 | GYKLCHPC----- | Positive |
| Q93WP7 | 42 | RGTNLPPPSPASS | Positive |
| Q93WP7 | 43 | GTNLPPPSPASSP | Positive |
| Q93WP7 | 45 | NLPPPSPASSPPS | Positive |
| Q93WP7 | 49 | PSPASSPPSKEVS | Positive |
| Q93WP7 | 50 | SPASSPPSKEVSN | Positive |
| Q800F1 | 54 | EAPERPPGFTPFR | Positive |
| P00877 | 104 | IAYVAYPIDLFEE | Positive |
| P00877 | 151 | VKTFVGPPHGIQV | Positive |
| Q28084 | 232 | PGLKGKPGDTGPP | Positive |
| Q28084 | 238 | PGDTGPPAAGAVM | Positive |
| Q93WP8 | 42 | RGANLPPPSPASS | Positive |
| Q93WP8 | 43 | GANLPPPSPASSP | Positive |
| Q93WP8 | 45 | NLPPPSPASSPPS | Positive |
| Q93WP8 | 49 | PSPASSPPSKEVS | Positive |
| Q93WP8 | 50 | SPASSPPSKEVSN | Positive |
| Q93WP8 | 150 | NRKPLSPPSPKPA | Positive |
| Q93WP8 | 151 | RKPLSPPSPKPAD | Positive |
| Q93WP8 | 153 | PLSPPSPKPADGQ | Positive |
| Q0VTT8 | 52 | KRFEPVPPGFTPF | Positive |
| P24091 | 67 | SQCPGGPTPPGGG | Positive |
| P24091 | 69 | CPGGPTPPGGGDL | Positive |
| P02453 | 1163 | LPGPIGPPGPRGR | Positive |
| Q9BPJ7 | 65 | CRFGCTPCCY--- | Positive |
| P04640 | 58 | NNGLGAPAPYPDP | Positive |
| P83455 | 56 | ERRDKPPAWVPGK | Positive |
| Q9ZT16 | 24 | SALAQAPAPTPTA | Positive |
| Q9ZT16 | 26 | LAQAPAPTPTATP | Positive |
| Q9ZT16 | 28 | QAPAPTPTATPPP | Positive |
| Q9ZT16 | 32 | PTPTATPPPATPP | Positive |
| Q9ZT16 | 33 | TPTATPPPATPPP | Positive |
| Q9ZT16 | 34 | PTATPPPATPPPV | Positive |
| Q9ZT16 | 37 | TPPPATPPPVATP | Positive |
| Q9ZT16 | 38 | PPPATPPPVATPP | Positive |
| Q9ZT16 | 39 | PPATPPPVATPPP | Positive |
| P69930 | 56 | LFHGGCPNNYGYN | Positive |
| Q9M0S4 | 24 | SAIAQAPGPAPTR | Positive |
| Q9M0S4 | 26 | IAQAPGPAPTRSP | Positive |
| Q9M0S4 | 28 | QAPGPAPTRSPLP | Positive |
| Q9M0S4 | 32 | PAPTRSPLPSPAQ | Positive |
| Q9M0S4 | 36 | RSPLPSPAQPPRT | Positive |
| Q8LG54 | 24 | SCLAQAPAPSPTT | Positive |
| Q8LG54 | 26 | LAQAPAPSPTTTV | Positive |
| Q8LG54 | 28 | QAPAPSPTTTVTP | Positive |
| Q8LG54 | 35 | TTTVTPPPVATPP | Positive |
| Q8LG54 | 36 | TTVTPPPVATPPP | Positive |
| P15502 | 34 | GVPGAIPGGVPGG | Positive |
| P15502 | 65 | GGKPLKPVPGGLA | Positive |
| P15502 | 67 | KPLKPVPGGLAGA | Positive |
| P15502 | 88 | FPAVTFPGALVPG | Positive |
| P15502 | 116 | AGLGGVPGVGGLG | Positive |
| P15502 | 156 | YPGGVLPGARFPG | Positive |
| P15502 | 167 | PGVGVLPGVPTGA | Positive |
| P15502 | 170 | GVLPGVPTGAGVK | Positive |
| P15502 | 177 | TGAGVKPKAPGVG | Positive |
| P15502 | 190 | GAFAGIPGVGPFG | Positive |
| P15502 | 283 | VGGAGVPGVPGAI | Positive |
| P15502 | 286 | AGVPGVPGAIPGI | Positive |
| P15502 | 290 | GVPGAIPGIGGIA | Positive |
| P15502 | 327 | GLVPGGPGFGPGV | Positive |
| P15502 | 342 | VPGAGVPGVGVPG | Positive |
| P15502 | 347 | VPGVGVPGAGIPV | Positive |
| P15502 | 352 | VPGAGIPVVPGAG | Positive |
| P15502 | 355 | AGIPVVPGAGIPG | Positive |
| P15502 | 360 | VPGAGIPGAAVPG | Positive |
| P15502 | 415 | VGVGGIPGVAGVP | Positive |
| P15502 | 421 | PGVAGVPSVGGVP | Positive |
| P15502 | 427 | PSVGGVPGVGGVP | Positive |
| P15502 | 465 | VLGGLVPGPQAAV | Positive |
| P15502 | 467 | GGLVPGPQAAVPG | Positive |
| P15502 | 481 | PGTGGVPGVGTPA | Positive |
| P15502 | 522 | PGVGVAPGVGLAP | Positive |
| P15502 | 550 | VAPGIGPGGVAAA | Positive |
| P15502 | 580 | GLGAGIPGLGVGV | Positive |
| P15502 | 607 | GVGAGVPGFGAGA | Positive |
| P15502 | 646 | PSSPRVPGALAAA | Positive |
| P15502 | 677 | LGGVGIPGGVVGA | Positive |
| P15502 | 769 | PGFGLSPIFPGGA | Positive |
| P15502 | 772 | GLSPIFPGGACLG | Positive |
| P01042 | 383 | SLMKRPPGFSPFR | Positive |
| Q9STQ3 | 31 | AAAVEAPAPSPTS | Positive |
| Q9STQ3 | 33 | AVEAPAPSPTSDA | Positive |
| Q9STQ3 | 35 | EAPAPSPTSDASL | Positive |
| P20908 | 570 | MGLTGRPGPVGPP | Positive |
| P20908 | 576 | PGPVGPPGSGGLK | Positive |
| P20908 | 621 | DGARGMPGQTGPK | Positive |
| P20908 | 639 | DGLAGLPGEKGHR | Positive |
| P20908 | 648 | KGHRGDPGPSGPP | Positive |
| P20908 | 654 | PGPSGPPGPPGDD | Positive |
| P20908 | 657 | SGPPGPPGDDGER | Positive |
| P20908 | 675 | VGPRGLPGEPGPR | Positive |
| P20908 | 678 | RGLPGEPGPRGLL | Positive |
| P20908 | 680 | LPGEPGPRGLLGP | Positive |
| P20908 | 686 | PRGLLGPKGPPGP | Positive |
| P20908 | 690 | LGPKGPPGPPGPP | Positive |
| P20908 | 696 | PGPPGPPGVTGMD | Positive |
| P20908 | 705 | TGMDGQPGPKGNV | Positive |
| P20908 | 717 | VGPQGEPGPPGQQ | Positive |
| P20908 | 720 | QGEPGPPGQQGNP | Positive |
| P20908 | 726 | PGQQGNPGAQGLP | Positive |
| P20908 | 732 | PGAQGLPGPQGAI | Positive |
| P20908 | 750 | KGPLGKPGLPGMP | Positive |
| P20908 | 756 | PGLPGMPGADGPP | Positive |
| P20908 | 762 | PGADGPPGHPGKE | Positive |
| P20908 | 765 | DGPPGHPGKEGPP | Positive |
| P20908 | 771 | PGKEGPPGEKGGQ | Positive |
| P20908 | 780 | KGGQGPPGPQGPI | Positive |
| P20908 | 789 | QGPIGYPGPRGVK | Positive |
| P20908 | 816 | KGEDGFPGFKGDM | Positive |
| P20908 | 834 | RGEIGPPGPRGED | Positive |
| P20908 | 861 | PGPLGPPGEKGKL | Positive |
| P20908 | 870 | KGKLGVPGLPGYP | Positive |
| P20908 | 873 | LGVPGLPGYPGRQ | Positive |
| P20908 | 876 | PGLPGYPGRQGPK | Positive |
| P20908 | 888 | KGSIGFPGFPGAN | Positive |
| P20908 | 891 | IGFPGFPGANGEK | Positive |
| P20908 | 903 | KGGRGTPGKPGPR | Positive |
| P20908 | 906 | RGTPGKPGPRGQR | Positive |
| P20908 | 930 | RGITGKPGPKGNS | Positive |
| P20908 | 945 | DGPAGPPGERGPN | Positive |
| P20908 | 1017 | MGERGHPGPPGPP | Positive |
| P20908 | 1020 | RGHPGPPGPPGEQ | Positive |
| P20908 | 1023 | PGPPGPPGEQGLP | Positive |
| P20908 | 1029 | PGEQGLPGLAGKE | Positive |
| P20908 | 1221 | EGPRGFPGPPGPV | Positive |
| P20908 | 1224 | RGFPGPPGPVGLQ | Positive |
| P20908 | 1467 | PGPMGPPGLPGLK | Positive |
| P20908 | 1470 | MGPPGLPGLKGDS | Positive |
| Q02388 | 2167 | AGPEGKPGLQGPR | Positive |
| Q02388 | 2176 | QGPRGPPGPVGGH | Positive |
| Q02388 | 2185 | VGGHGDPGPPGAP | Positive |
| Q02388 | 2188 | HGDPGPPGAPGLA | Positive |
| Q02388 | 2664 | KGEMGEPGVPGQS | Positive |
| Q02388 | 2667 | MGEPGVPGQSGAP | Positive |
| Q02388 | 2673 | PGQSGAPGKEGLI | Positive |
| P29602 | 115 | NATVSMPPPSSSP | Positive |
| P29602 | 116 | ATVSMPPPSSSPP | Positive |
| P29602 | 117 | TVSMPPPSSSPPS | Positive |
| P29602 | 121 | PPPSSSPPSSVMP | Positive |
| P29602 | 122 | PPSSSPPSSVMPP | Positive |
| P29602 | 127 | PPSSVMPPPVMPP | Positive |
| P29602 | 128 | PSSVMPPPVMPPP | Positive |
| P29602 | 129 | SSVMPPPVMPPPS | Positive |
| P29602 | 132 | MPPPVMPPPSPS- | Positive |
| P29602 | 133 | PPPVMPPPSPS-- | Positive |
| P29602 | 134 | PPVMPPPSPS--- | Positive |
| P29602 | 136 | VMPPPSPS----- | Positive |
| Q15848 | 44 | GWMAGIPGHPGHN | Positive |
| Q15848 | 47 | AGIPGHPGHNGAP | Positive |
| Q15848 | 53 | PGHNGAPGRDGRD | Positive |
| Q15848 | 71 | KGEKGDPGLIGPK | Positive |
| Q15848 | 76 | DPGLIGPKGDIGE | Positive |
| Q15848 | 91 | VPGAEGPRGFPGI | Positive |
| Q15848 | 95 | EGPRGFPGIQGRK | Positive |
| Q7Z096 | 38 | RVKNRGPSFCKAD | Positive |
| Q7Z096 | 47 | CKADEKPCKYHAD | Positive |
| Q7Z096 | 65 | LGGICKPSTSWIG | Positive |
| P0C8V9 | 55 | AGNYCGPTVMKIC | Positive |
| P0C8V9 | 67 | CCGFCSPFSKICM | Positive |
| P85886 | 55 | RTCCSRPTCRMEY | Positive |
| P28880 | 55 | CRSSGSPCGVTSI | Positive |
| P58786 | 57 | LRRSGCPWEPWCG | Positive |
| P0C1W6 | 14 | INTRDSPWGRCCR | Positive |
| Q24940 | 109 | ANNRAVPDKIDWR | Positive |
| Q24940 | 196 | ETESSYPYTAVEG | Positive |
| P69765 | 49 | RCCPGKPCCRIG- | Positive |
| P58913 | 57 | KEACYAPGTFCGI | Positive |
| P58913 | 65 | TFCGIKPGLCCSE | Positive |
| Q15485 | 77 | RGERGPPGPPGKA | Positive |
| Q15485 | 80 | RGPPGPPGKAGPP | Positive |
| Q15485 | 86 | PGKAGPPGPNGAP | Positive |
| P12111 | 2100 | KGSRGFPGEKGEV | Positive |
| P12111 | 2206 | FGRRGPPGAKGNK | Positive |
| P12111 | 2239 | AGPAGPPGLIGEQ | Positive |
| P12111 | 2316 | KGERGFPGYPGPK | Positive |
| P12111 | 2319 | RGFPGYPGPKGNP | Positive |
| Q9C5S0 | 26 | GQAPTSPPTATPA | Positive |
| Q9C5S0 | 27 | QAPTSPPTATPAP | Positive |
| Q9C5S0 | 31 | SPPTATPAPPTPT | Positive |
| Q9C5S0 | 33 | PTATPAPPTPTTP | Positive |
| P02747 | 36 | TGCYGIPGMPGLP | Positive |
| P02747 | 39 | YGIPGMPGLPGAP | Positive |
| P02747 | 42 | PGMPGLPGAPGKD | Positive |
| P02747 | 45 | PGLPGAPGKDGYD | Positive |
| P02747 | 54 | DGYDGLPGPKGEP | Positive |
| P02747 | 63 | KGEPGIPAIPGIR | Positive |
| P02747 | 81 | KGEPGLPGHPGKN | Positive |
| P02747 | 93 | NGPMGPPGMPGVP | Positive |
| P02747 | 96 | MGPPGMPGVPGPM | Positive |
| P02747 | 99 | PGMPGVPGPMGIP | Positive |
| P02747 | 105 | PGPMGIPGEPGEE | Positive |
| P12108 | 158 | PGKPGPPGHIQGV | Positive |
| P12108 | 178 | CPTNCPPGPKGPQ | Positive |
| O97939 | 877 | AGGSMWPKNNPLA | Negative |
| Q9ZT16 | 88 | VPTASPPAPEGPT | Negative |
| Q8LG54 | 71 | PSSAPSPSSDAPT | Negative |
| P01042 | 612 | FPDTTSPKCPGRP | Negative |
| P08123 | 809 | ISGPPGPPGPAGK | Negative |
| P08123 | 293 | LPGLSGPVGPPGN | Negative |
| P12111 | 2903 | VTIINQPSVKPAA | Negative |
| Q9UKV8 | 373 | ATARSAPDRQEEI | Negative |
| P12108 | 558 | AMGPPGPPGPPGP | Negative |
| P12108 | 580 | MGPRGVPGLLGAA | Negative |
| P08123 | 1071 | DGRTGHPGTVGPA | Negative |
| P12108 | 128 | PGQVGLPGEIGVP | Negative |
| Q02388 | 2649 | KGEAGPPGRPGLA | Negative |
| P05997 | 1156 | LQGLPGPPGPNGE | Negative |
| O97939 | 803 | HQKENQPYSNNSP | Negative |
| O97939 | 288 | PTGQNGPAVNVSG | Negative |
| Q05707 | 1740 | SPGPRGPPGHLGV | Negative |
| Q05707 | 625 | FTTEEVPAQQYLE | Negative |
| Q16665 | 658 | TSATSSPYRDTQS | Negative |
| Q02388 | 1478 | PPGAIGPKGDRGF | Negative |
| O97939 | 478 | EDNMLVPNFNSID | Negative |
| P08125 | 242 | RGEAGIPGPQGPP | Negative |
| P12111 | 2987 | KPATTKPMVKMSR | Negative |
| Q02388 | 1464 | PGQPGSPGEQGPR | Negative |
| Q9ZT16 | 43 | PPPVATPPPVATP | Negative |
| Q9UKV8 | 14 | ALAPPAPPPPIQG | Negative |
| P20908 | 1355 | PPGEPGPAGQDGP | Negative |
| P05997 | 601 | EDGRPGPPGSIGI | Negative |
| P02453 | 663 | PGEQGVPGDLGAP | Negative |
| P20908 | 995 | KTGPPGPPGVVGP | Negative |
| P35248 | 136 | PQGKPGPKGEAGP | Negative |
| Q28084 | 142 | SGPKGPPGVRGEP | Negative |
| P02745 | 29 | EDLCRAPDGKKGE | Negative |
| O97939 | 607 | DPRENSPYLRSNT | Negative |
| O97939 | 403 | RRKPEGPNKNPMV | Negative |
| Q16665 | 492 | ELSFTMPQIQDQT | Negative |
| Q02388 | 88 | VQYSDDPRTEFGL | Negative |
| P30754 | 1001 | RVGPRGPSGDDGR | Negative |
| P02453 | 1187 | PPGPPGPPGPPSG | Negative |
| Q15485 | 53 | TILRGCPGLPGAP | Negative |
| P05997 | 400 | ARGPEGPQGQRGE | Negative |
| P84349 | 66 | PVPYPDPLEPRRE | Negative |
| P20908 | 342 | GDYDYVPSEDYYT | Negative |
| P04640 | 62 | GAPAPYPDPLEPH | Negative |
| Q28084 | 439 | WLASLDPKRMFRK | Negative |
| P20908 | 1164 | KGEIGEPGQKGSK | Negative |
| Q02388 | 2726 | SAGPPGPPGSVGP | Negative |
| P05997 | 1010 | RGMPGLPGPAGTP | Negative |
| P20908 | 1805 | VLEIDTPKVEQVP | Negative |
| P12108 | 559 | MGPPGPPGPPGPP | Negative |
| Q15485 | 61 | LPGAPGPKGEAGT | Negative |
| P05997 | 1195 | NPGPLGPIGPPGV | Negative |
| P05997 | 1046 | NGPVGEPGPEGPA | Negative |
| P15502 | 232 | LPYGYGPGGVAGA | Negative |
| Q9C5S0 | 83 | PPPASPPPATPPP | Negative |
| P08252 | 36 | AGGARCPSGLCCS | Negative |
| Q9C5S0 | 106 | PPPATPPPVATPP | Negative |
| P05997 | 1090 | APGTPGPVGAPGD | Negative |
| Q05707 | 810 | YKVTVTPIYTDGE | Negative |
| Q9XF04 | 86 | HHHVNPPRQPRNN | Negative |
| P0C8W0 | 73 | GPCCCKPNFTCQI | Negative |
| P12108 | 576 | LHGPMGPRGVPGL | Negative |
| P24091 | 303 | SILGVSPGDNLDC | Negative |
| Q05707 | 1777 | APHPDQPEFTPVQ | Negative |
| Q02388 | 1811 | PGRDGLPGLRGEQ | Negative |
| P08125 | 175 | KGEPGVPGINGQK | Negative |
| P24091 | 234 | LWFWMTPQSPKPS | Negative |
| P20908 | 1100 | PAGAAGPIGIPGR | Negative |
| Q9ZT16 | 72 | PAPATTPPSVAPS | Negative |
| P05997 | 1180 | PRGPPGPVGPSGK | Negative |
| P05997 | 971 | PGDKGDPGEDGQP | Negative |
| P02453 | 459 | TGIQGPPGPAGEE | Negative |
| Q9Y2N7 | 229 | CEAIPHPGSLEPP | Negative |
| P20908 | 1130 | EKGPQGPAGRDGL | Negative |
| P08123 | 839 | EVGAVGPPGFAGE | Negative |
| P02453 | 237 | DGEAGKPGRPGER | Negative |
| P08125 | 314 | PPGFPGPKGDQGP | Negative |
| P20908 | 1377 | PGQTGSPGPTGEP | Negative |
| Q02388 | 2172 | KPGLQGPRGPPGP | Negative |
| P02747 | 78 | KGQKGEPGLPGHP | Negative |
| P20908 | 923 | PRGERGPRGITGK | Negative |
| Q9ZT16 | 44 | PPVATPPPVATPP | Negative |
| Q16665 | 511 | STRQSSPEPNSPS | Negative |
| P35248 | 130 | KQGNIGPQGKPGP | Negative |
| P12108 | 346 | PGSKGGPGDKGEV | Negative |
| Q02388 | 1847 | NGKNGEPGDPGED | Negative |
| P02747 | 23 | LLLLLLPLRGQAN | Negative |
| O97939 | 569 | QQEISPPFKEDPG | Negative |
| P12111 | 2830 | NAFYLSPDIRKQC | Negative |
| P19999 | 60 | LRGLQGPPGKLGP | Negative |
| P08123 | 1049 | APGSVGPAGPRGP | Negative |
| O97939 | 763 | SWSSWDPRIQAQG | Negative |
| P20908 | 1022 | HPGPPGPPGEQGL | Negative |
| P05623 | 36 | KFPIPSPNPRDKW | Negative |
| P02453 | 1459 | FGFDVGPACFL-- | Negative |
| P05997 | 739 | MAGGHGPDGPKGS | Negative |
| P01042 | 580 | PPISPAPIQSDDD | Negative |
| P02453 | 645 | PGFQGLPGPAGPP | Negative |
| P05997 | 250 | DPGPMGPIGSRGP | Negative |
| P02453 | 770 | LTGPIGPPGPAGA | Negative |
| P02747 | 244 | SGFLLFPD----- | Negative |
| Q9UKV8 | 585 | LPQGRPPVFQQPV | Negative |
| Q9Y2N7 | 201 | MRAYKPPAQTSPA | Negative |
| P30754 | 998 | PSGRVGPRGPSGD | Negative |
| P20908 | 1122 | AGEKGAPGEKGPQ | Negative |
| P08125 | 136 | ARGPQGPPGIPGP | Negative |
| P20908 | 292 | LGKEPTPSKKPVE | Negative |
| P58786 | 60 | SGCPWEPWCG--- | Negative |
| P02453 | 650 | LPGPAGPPGEAGK | Negative |
| P12108 | 246 | PKGETGPAGYKGM | Negative |
| Q9C5S0 | 47 | PAATPPPVSAPPP | Negative |
| P20908 | 435 | EIGPGMPANQDTI | Negative |
| Q9Y2N7 | 431 | ASVAATPSTPLAT | Negative |
| P12111 | 2733 | FESAPNPRDLKIV | Negative |
| P12111 | 2133 | SGEKGNPGRRGDK | Negative |
| P05997 | 1222 | EPGPPGPPGPPGH | Negative |
| P20908 | 1041 | EGTKGDPGPAGLP | Negative |
| Q15848 | 86 | IGETGVPGAEGPR | Negative |
| Q02388 | 2648 | DKGEAGPPGRPGL | Negative |
| P08125 | 162 | PPGAQGPRGPPGE | Negative |
| O97939 | 730 | PSYNTAPTVSSPV | Negative |
| P08661 | 161 | QGTVATPRNAEEN | Negative |
| Q02388 | 1020 | RVTGLEPGVSYIF | Negative |
| P58925 | 21 | FPITALPMDGDQP | Negative |
| Q25460 | 267 | PTYKAKPTYKAKP | Negative |
| Q28084 | 237 | KPGDTGPPAAGAV | Negative |
| Q05707 | 1720 | PPGPAGPSGESRP | Negative |
| P20908 | 1250 | DVGQMGPPGPPGP | Negative |
| P05997 | 397 | PTGARGPEGPQGQ | Negative |
| Q28084 | 35 | FPGVTIPGQKGDR | Negative |
| Q3Y5Z3 | 150 | KFLCNIPGLYYFS | Negative |
| Q28084 | 193 | CGPKGKPGEDGPP | Negative |
| P05997 | 904 | GRGTQGPPGATGF | Negative |
| P05997 | 100 | PVCSQTPGGGNTN | Negative |
| Q02388 | 2771 | RGEQGRPGPAGPR | Negative |
| P02453 | 671 | DLGAPGPSGARGE | Negative |
| Q9M0S4 | 57 | PTPTPTPSATPTA | Negative |
| P02453 | 777 | PGPAGAPGDKGEA | Negative |
| P08125 | 305 | MKGHRGPEGPPGF | Negative |
| Q02388 | 1739 | KGDPGLPGAPGER | Negative |
| P12111 | 2260 | GGAAGAPGERGRT | Negative |
| P05997 | 514 | DPGTVGPPGPVGE | Negative |
| P08123 | 453 | MGPRGLPGSPGNI | Negative |
| Q25460 | 60 | KLSSYKPIKTTYN | Negative |
| P02453 | 495 | PGSRGFPGADGVA | Negative |
| Q9C5S0 | 53 | PVSAPPPVTTSPP | Negative |
| P69929 | 201 | PSRKREPEPNVAV | Negative |
| P05997 | 454 | PPGSPGPQGSTGP | Negative |
| Q02388 | 2747 | QKGERGPPGERVV | Negative |
| P20908 | 1223 | PRGFPGPPGPVGL | Negative |
| P35248 | 149 | KGEVGAPGMQGSA | Negative |
| Q02388 | 1699 | KGDRGEPGPPGPP | Negative |
| Q02388 | 1046 | ASVTQTPVCPRGL | Negative |
| P08125 | 215 | SAGIGKPGENGLP | Negative |
| P02453 | 1058 | APGPVGPAGKSGD | Negative |
| P20908 | 1296 | AGEPGLPGEGGPP | Negative |
| P05997 | 467 | QGIRGQPGDPGVP | Negative |
| Q9ZT16 | 66 | PPPAATPAPATTP | Negative |
| Q02388 | 2714 | IGGFPGPSGNDGS | Negative |
| P12108 | 594 | QIGNIGPKGKRGE | Negative |
| P15502 | 540 | PGVGVAPGVGVAP | Negative |
| Q9ZT16 | 101 | VSPSSAPGPSDAS | Negative |
| Q9UKV8 | 609 | AGDGKKPSIAAVV | Negative |
| Q05707 | 1761 | QPGYCDPSSCSAY | Negative |
| P12111 | 1590 | QTITNDPRLVFTV | Negative |
| P12108 | 672 | ACLGALPTPRHG- | Negative |
| Q9M0S4 | 80 | LPSSASPPAPPTS | Negative |
| Q05707 | 377 | VNWTHAPGNVEKY | Negative |
| P02453 | 602 | ERGVPGPPGAVGP | Negative |
| Q9ZT16 | 55 | PPPAATPAPATPP | Negative |
| P20908 | 1287 | VGEKGEPGEAGEP | Negative |
| Q9XF04 | 78 | VPSGPDPLHHHVN | Negative |
| P08123 | 767 | AAGPAGPNGPPGP | Negative |
| P08123 | 1101 | PGPPGPPGVSGGG | Negative |
| Q9Y2N7 | 564 | DPSASSPMAGARK | Negative |
| P12111 | 1104 | LTLLGGPTPNTGA | Negative |
| Q9Y2N7 | 618 | FLLTGGPAPGSLQ | Negative |
| P02453 | 1149 | PGSAGSPGKDGLN | Negative |
| P05997 | 644 | PGQRGAPGKDGEV | Negative |
| P05997 | 184 | HPSHPGPDGLSRP | Negative |
| Q05707 | 1463 | NEVALGPAGPPGG | Negative |
| P05997 | 1249 | SMPDPLPEFTEDQ | Negative |
| P08123 | 471 | EGPVGLPGIDGRP | Negative |
| P05997 | 247 | EPGDPGPMGPIGS | Negative |
| Q16665 | 688 | KSHPRSPNVLSVA | Negative |
| Q05707 | 15 | RYWLLPPFLAIVY | Negative |
| P12108 | 372 | LDGEPGPPGDAGT | Negative |
| Q28084 | 198 | KPGEDGPPGTPGP | Negative |
| Q557E4 | 44 | DAPIPLPNVTSTI | Negative |
| Q02388 | 1911 | VPGGTGPKGDRGE | Negative |
| P02453 | 1136 | PSGASGPAGPRGP | Negative |
| P24091 | 177 | TPSGQWPCAPGRK | Negative |
| P05997 | 1042 | PPGSNGPVGEPGP | Negative |
| O97939 | 1089 | DGDPIMPTETPNS | Negative |
| P23805 | 128 | KQGSMGPPGTPGP | Negative |
| P12108 | 492 | VRGYPGPPGPRGL | Negative |
| P24091 | 149 | GGWATAPDGPYAW | Negative |
| P12108 | 561 | PPGPPGPPGPPGE | Negative |
| Q02388 | 1523 | AKGPEGPPGPTGR | Negative |
| Q15485 | 97 | APGEPQPCLTGPR | Negative |
| P86289 | 26 | PSGPQGPSGAPGP | Negative |
| P52285 | 110 | NYLDIKPLLDVTC | Negative |
| Q9SJY7 | 76 | SPVASPPQTDAPA | Negative |
| P08123 | 504 | KGPTGDPGKNGDK | Negative |
| Q60994 | 132 | VTVPNVPIRFTKI | Negative |
| P58846 | 27 | PLDGDQPADQPAE | Negative |
| P11226 | 72 | LRGLQGPPGKLGP | Negative |
| P02453 | 729 | PGLQGMPGERGAA | Negative |
| Q02388 | 688 | RTDPLGPVRTVHV | Negative |
| P15502 | 23 | LLSILHPSRPGGV | Negative |
| Q02388 | 2400 | PGLPGAPGVVGFP | Negative |
| P0C8V6 | 43 | RHEMKNPEASKLN | Negative |
| P35248 | 106 | ERGLVGPPGSPGI | Negative |
| Q9Y2N7 | 453 | DLPDELPVGTENV | Negative |
| P08123 | 434 | PAGVRGPNGDAGR | Negative |
| Q9XF04 | 96 | RNNFQLP------ | Negative |
| Q28084 | 115 | PGFYGFPGMKGKK | Negative |
| Q9C5S0 | 52 | PPVSAPPPVTTSP | Negative |
| Q02388 | 1972 | SSGSFLPVPERRR | Negative |
| P12111 | 2859 | HKQVNVPNNVTSS | Negative |
| Q02388 | 227 | GVPVTRPPDDSTS | Negative |
| P02453 | 480 | PGPAGLPGPPGER | Negative |
| P05997 | 485 | AGPKGEPGPHGIQ | Negative |
| P30754 | 719 | QPGLPGPSGQPGP | Negative |
| P02453 | 567 | DGRPGPPGPPGAR | Negative |
| P12108 | 555 | GVGAMGPPGPPGP | Negative |
| Q9C5S0 | 115 | ATPPPAPLASPPA | Negative |
| P12108 | 111 | PGPPGLPGPSLPG | Negative |
| P15502 | 433 | PGVGGVPGVGISP | Negative |
| P02453 | 1160 | LNGLPGPIGPPGP | Negative |
| P20908 | 1536 | PGPPGLPGPPGPK | Negative |
| P02747 | 71 | IPGIRGPKGQKGE | Negative |
| P0C8V5 | 34 | GLKDLFPKARHEM | Negative |
| P12111 | 2363 | DPGYPGPAGPKGN | Negative |
| Q02388 | 1702 | RGEPGPPGPPGRL | Negative |
| Q16665 | 567 | MLAPYIPMDDDFQ | Negative |
| P20908 | 321 | TEAAPMPETSEGA | Negative |
| P02747 | 56 | YDGLPGPKGEPGI | Negative |
| O97939 | 173 | LFPYQQPLWHVPH | Negative |
| Q9M0S4 | 55 | ITPTPTPTPSATP | Negative |
| P05997 | 1380 | YGDHQSPNTAITQ | Negative |
| P12108 | 226 | QGVPGPPGPQGQR | Negative |
| P52285 | 94 | FCKVDQPTLFELI | Negative |
| P58786 | 35 | ADRNAVPRDDNPG | Negative |
| P05997 | 223 | PVGPRGPQGLQGQ | Negative |
| Q9Y2N7 | 440 | PLATRHPQSPLSA | Negative |
| O97939 | 147 | NEPSPTPTQPEEE | Negative |
| P02453 | 37 | QEEDIPPVTCVQN | Negative |
| P08123 | 1122 | FYRADQPRSAPSL | Negative |
| P12108 | 490 | PGVRGYPGPPGPR | Negative |
| Q9UKV8 | 661 | KSTRFKPTRIIFY | Negative |
| O97939 | 93 | FPQYQMPMWPQPP | Negative |
| P12111 | 1398 | STFRELPSLEQKL | Negative |
| O97939 | 676 | LSFKEDPTVRHYE | Negative |
| P02453 | 474 | RGARGEPGPAGLP | Negative |
| Q02388 | 2364 | QKGAPGPKGFKGD | Negative |
| P20908 | 1722 | VDAEGNPVGVVQM | Negative |
| Q02388 | 622 | VAWGPVPGASGFR | Negative |
| Q7XAD0 | 124 | SPPPPKPQDEQRQ | Negative |
| P02453 | 189 | SGPRGLPGPPGAP | Negative |
| P20908 | 1010 | PTGETGPMGERGH | Negative |
| Q05707 | 1299 | RILPDTPQEPFAL | Negative |
| P30754 | 125 | PDGGPGPAGPSGP | Negative |
| P35248 | 254 | KKAALFPDGQSVG | Negative |
| P12108 | 39 | RGPPGPPGVPGAD | Negative |
| P12108 | 370 | PGLDGEPGPPGDA | Negative |
| P30754 | 992 | KDGQPGPSGRVGP | Negative |
| B2KPN7 | 62 | WEYHAHPKPNSFW | Negative |
| P12111 | 2157 | VGIRGDPGNPGQD | Negative |
| P02453 | 87 | CPNAKVPTDECCP | Negative |
| P52285 | 40 | IGESDSPIPLPNV | Negative |
| P12108 | 382 | AGTAGVPGLKGDR | Negative |
| P02453 | 924 | PGEVGPPGPPGPA | Negative |
| Q9Y2N7 | 84 | VGAGGEPLDACYL | Negative |
| O97939 | 134 | QPQPKTPTPKQPL | Negative |
| P04925 | 83 | GGSWGQPHGGGWG | Negative |
| P20908 | 1811 | PKVEQVPIVDIMF | Negative |
| P12111 | 1513 | RLRGGSPLNTGKA | Negative |
| P05997 | 380 | PGSSGFPGNPGMK | Negative |
| P35248 | 134 | IGPQGKPGPKGEA | Negative |
| P02453 | 119 | PKGDTGPRGPRGP | Negative |
| Q25460 | 691 | PTYKAKPTNPSTY | Negative |
| Q9Y2N7 | 443 | TRHPQSPLSADLP | Negative |
| Q9ZT16 | 57 | PAATPAPATPPPA | Negative |
| P05997 | 686 | PGPPGPPGEGGKP | Negative |
| P05997 | 1473 | QNVARLPIIDLAP | Negative |
| P12111 | 1922 | NMRSQHPYVLTED | Negative |
| O97939 | 143 | KQPLNEPSPTPTQ | Negative |
| P12111 | 2328 | KGNPGEPGLNGTT | Negative |
| P05997 | 833 | PGSRGNPGSRGEN | Negative |
| Q05707 | 776 | MTAQGDPEEEVIG | Negative |
| Q02388 | 868 | TPPEAPPALGTLH | Negative |
| Q02388 | 476 | QLDGLQPGTEYRL | Negative |
| P35248 | 142 | PKGEAGPKGEVGA | Negative |
| Q9Y2N7 | 159 | RRKVEAPTERCFS | Negative |
| P20908 | 1094 | SPGERGPAGAAGP | Negative |
| P12111 | 2896 | VTTTTKPVTIINQ | Negative |
| P20908 | 86 | DAQLSAPTKQLYP | Negative |
| P05997 | 245 | PGEPGDPGPMGPI | Negative |
| P20908 | 1485 | KGEKGHPGLIGLI | Negative |
| P15502 | 26 | ILHPSRPGGVPGA | Negative |
| Q16665 | 230 | CEPIPHPSNIEIP | Negative |
| P23805 | 59 | QDGRECPHGEKGD | Negative |
| Q8LG54 | 53 | APTTTPPPAVSPA | Negative |
| P35248 | 85 | PRGPVGPKGENGS | Negative |
| O97939 | 98 | MPMWPQPPPNKKH | Negative |
| P08123 | 101 | LMGPRGPPGAAGA | Negative |
| P02453 | 515 | ERGAPGPAGPKGS | Negative |
| Q05707 | 1016 | TRPPTFPPTIPPA | Negative |
| Q9Y2N7 | 554 | RLSCSSPSRGDPS | Negative |
| Q05707 | 557 | ARLTWDPTSRQIN | Negative |
| Q25460 | 453 | PTYKAKPTYKAKP | Negative |
| Q9SJY7 | 33 | APTTVTPPPTALP | Negative |
| P05997 | 460 | PQGSTGPQGIRGQ | Negative |
| P20908 | 1427 | KTGPIGPQGAPGK | Negative |
| Q02388 | 1552 | GPAVAGPKGEKGD | Negative |
| P20908 | 476 | PPGPEGPAGLPGP | Negative |
| Q02388 | 1539 | KGEPGRPGDPAVV | Negative |
| P12108 | 301 | KGDMGLPGIDGKD | Negative |
| P05997 | 164 | QGIDGEPGVPGQP | Negative |
| P12111 | 2595 | DICNIDPSCGFGS | Negative |
| P08123 | 248 | PVGPAGPIGSAGP | Negative |
| P23805 | 5 | #NAME? | Negative |
| P02453 | 822 | PGADGQPGAKGEP | Negative |
| P84349 | 99 | YRRFYGPV----- | Negative |
| P20908 | 1349 | FPGDPGPPGEPGP | Negative |
| P20908 | 575 | RPGPVGPPGSGGL | Negative |
| Q9UKV8 | 191 | SEGCSNPLGGGRE | Negative |
| P12111 | 2361 | KGDPGYPGPAGPK | Negative |
| P08123 | 245 | SVGPVGPAGPIGS | Negative |
| Q02388 | 1333 | SPGLPGPRGDPGE | Negative |
| P12111 | 3017 | RPEPPGPYFYDLT | Negative |
| P08125 | 92 | PPGPLGPPGFSTV | Negative |
| Q16665 | 468 | LRSSADPALNQEV | Negative |
| Q25460 | 73 | AKTNYPPVYKPKM | Negative |
| P08125 | 426 | QGVKGVPGINGEP | Negative |
| P0C1N1 | 16 | ICLLLFPLTAVPL | Negative |
| P20908 | 761 | MPGADGPPGHPGK | Negative |
| O97939 | 431 | NENIQNPREKQVS | Negative |
| P08123 | 46 | PRGERGPPGPPGR | Negative |
| P08123 | 49 | ERGPPGPPGRDGE | Negative |
| O75636 | 112 | WYHLCLPEGRALP | Negative |
| P42849 | 69 | ISHMTTPPVKIML | Negative |
| P20908 | 480 | EGPAGLPGPPGTM | Negative |
| P20908 | 692 | PKGPPGPPGPPGV | Negative |
| P23805 | 249 | KKAVLFPDGQAVG | Negative |
| P30754 | 404 | EQGPQGPQGLAGL | Negative |
| Q16665 | 267 | ELMGYEPEELLGR | Negative |
| P04925 | 104 | WNKPSKPKTNLKH | Negative |
| P08123 | 210 | PGENGTPGQTGAR | Negative |
| Q02388 | 554 | ERTLVLPGSQTAF | Negative |
| P20908 | 1089 | PGPAGSPGERGPA | Negative |
| Q3Y5Z3 | 93 | RGFPGTPGRKGEP | Negative |
| P02453 | 461 | IQGPPGPAGEEGK | Negative |
| Q93WP8 | 113 | FISYLLPVSYVWN | Negative |
| Q05707 | 277 | SQDDIIPPSRNLR | Negative |
| P05997 | 497 | QGPIGPPGEEGKR | Negative |
| P20908 | 996 | TGPPGPPGVVGPQ | Negative |
| Q05707 | 309 | QEIASEPDSTHVY | Negative |
| P02453 | 281 | AKGDAGPAGPKGE | Negative |
| Q9M0S4 | 53 | PSITPTPTPTPSA | Negative |
| Q02388 | 645 | SSQTLPPDSTATD | Negative |
| P02746 | 131 | TRTINVPLRRDQT | Negative |
| P01042 | 578 | MMPPISPAPIQSD | Negative |
| P02453 | 222 | MGPRGPPGPPGKN | Negative |
| Q02388 | 2199 | LAGPAGPQGPSGL | Negative |
| P12111 | 2215 | KGNKGGPGQPGFE | Negative |
| P12108 | 42 | PGPPGVPGADGID | Negative |
| O97939 | 1024 | QLVFGTPDKEPRP | Negative |
| P20908 | 1397 | KRGPPGPAGPEGR | Negative |
| Q05707 | 1020 | TFPPTIPPAKEVC | Negative |
| Q9SJY7 | 83 | QTDAPAPGPSAGL | Negative |
| Q02388 | 1410 | DRGERGPPGPGEG | Negative |
| P20908 | 1052 | LPGKDGPPGLRGF | Negative |
| P20908 | 944 | GDGPAGPPGERGP | Negative |
| P02453 | 963 | RGVVGLPGQRGER | Negative |
| Q02388 | 2178 | PRGPPGPVGGHGD | Negative |
| P08125 | 248 | PGPQGPPGEPGEV | Negative |
| P23805 | 140 | PKGETGPKGGVGA | Negative |
| P02453 | 522 | AGPKGSPGEAGRP | Negative |
| O97939 | 630 | MGQPENPHYPMNT | Negative |
| P08123 | 371 | SAGPQGPPGPSGE | Negative |
| P05997 | 235 | QQGGAGPTGPPGE | Negative |
| Q02388 | 1820 | RGEQGLPGPSGPP | Negative |
| P02453 | 557 | KTGPPGPAGQDGR | Negative |
| O97939 | 84 | GNGMQLPQFFPQY | Negative |
| P20908 | 950 | PPGERGPNGPQGP | Negative |
| Q02388 | 1515 | PGVAGRPGAKGPE | Negative |
| P20908 | 1514 | PQGSSGPKGEQGI | Negative |
| Q02388 | 1277 | PGDPGLPGRTGAP | Negative |
| O97939 | 1073 | TSSIGTPSSLGRQ | Negative |
| P02453 | 828 | PGAKGEPGDAGAK | Negative |
| Q15485 | 85 | PPGKAGPPGPNGA | Negative |
| Q28084 | 471 | VCMKMRP------ | Negative |
| P24091 | 237 | WMTPQSPKPSCHD | Negative |
| P30754 | 80 | PRGGIGPMGPRGA | Negative |
| Q02388 | 816 | GRSEGGPMRHQIL | Negative |
| Q9C5S0 | 79 | PVSSPPPASPPPA | Negative |
| Q02388 | 1319 | AGNPGTPGAPGLK | Negative |
| Q9BPJ7 | 16 | ICLLLFPLTAVPM | Negative |
| P23805 | 107 | DTGPRGPPGMPGP | Negative |
| P02453 | 1085 | PVGARGPAGPQGP | Negative |
| Q9SJY7 | 39 | PPPTALPPVTAET | Negative |
| Q05707 | 1315 | LNKNSDPLVGVIL | Negative |
| P05997 | 334 | PMGAMGPLGPRGM | Negative |
| O97939 | 108 | KKHPQKPSASKQQ | Negative |
| P08125 | 414 | AGHPGLPGPVGPQ | Negative |
| P24091 | 189 | KYFGRGPIQISHN | Negative |
| P02453 | 858 | IGNVGAPGPKGAR | Negative |
| P08125 | 104 | VGKLGMPGLPGKP | Negative |
| P19999 | 203 | NWKKDEPNDHGSG | Negative |
| Q9C5S0 | 46 | PPAATPPPVSAPP | Negative |
| P15502 | 720 | VGGLGVPGVGGLG | Negative |
| Q05707 | 91 | IIQGLMPDQNYTV | Negative |
| P02453 | 564 | AGQDGRPGPPGPP | Negative |
| P05997 | 599 | PGEDGRPGPPGSI | Negative |
| P05997 | 1121 | AGKRGLPGPQGPR | Negative |
| P08123 | 324 | AGAPGLPGPRGIP | Negative |
| O97939 | 213 | GFGGRPPYYSEEM | Negative |
| P05997 | 985 | PDGPPGPAGTTGQ | Negative |
| P08123 | 638 | TAGPSGPSGLPGE | Negative |
| Q800F1 | 53 | REAPERPPGFTPF | Negative |
| Q02388 | 1030 | YIFSLTPVLDGVR | Negative |
| Q28084 | 181 | PGPPGPPGDPGPC | Negative |
| P02453 | 185 | PMGPSGPRGLPGP | Negative |
| P02453 | 1079 | PAGPIGPVGARGP | Negative |
| P12111 | 1327 | RNIFKRPLGSRIE | Negative |
| P05997 | 1036 | PPGPVGPPGSNGP | Negative |
| P12108 | 615 | RGHPGMPGPPGIP | Negative |
| Q02388 | 2175 | LQGPRGPPGPVGG | Negative |
| P02747 | 131 | TRQTHQPPAPNSL | Negative |
| P08123 | 783 | RGDGGPPGMTGFP | Negative |
| O75636 | 18 | LLLLGGPACLKTQ | Negative |
| P05997 | 722 | PGERGEPGITGLP | Negative |
| P84349 | 64 | GAPVPYPDPLEPR | Negative |
| O97939 | 449 | VVPTRDPSGPWRN | Negative |
| P12111 | 815 | PQQLIQPLTTYVS | Negative |
| Q9UKV8 | 176 | PSMRYTPVGRSFF | Negative |
| P05997 | 56 | NRDIWKPAPCQIC | Negative |
| P08125 | 128 | AGPVGLPGARGPQ | Negative |
| P02453 | 771 | TGPIGPPGPAGAP | Negative |
| O97939 | 417 | NVAPPGPKHGTVD | Negative |
| P20908 | 1460 | SPGPDGPPGPMGP | Negative |
| Q16665 | 454 | LAMSPLPTAETPK | Negative |
| P69929 | 65 | PSRKREPEPNVAV | Negative |
| Q02388 | 1364 | GEGPGLPGRKGDP | Negative |
| Q28084 | 55 | NPGMPGPPGPPGS | Negative |
| P02453 | 1073 | ETGPAGPAGPIGP | Negative |
| Q02388 | 1274 | VGPPGDPGLPGRT | Negative |
| Q9Y2N7 | 406 | AALAADPRRFCSP | Negative |
| P04925 | 239 | TVLFSSPPVILLI | Negative |
| Q02388 | 763 | VAGVDGPPASVVV | Negative |
| P02453 | 218 | ASGPMGPRGPPGP | Negative |
| Q16665 | 582 | SFDQLSPLESSSA | Negative |
| Q25460 | 502 | KPKKTYPPTYKPK | Negative |
| P30754 | 353 | EPGAKGPTGDTGR | Negative |
| P05997 | 505 | EEGKRGPRGDPGT | Negative |
| P02453 | 842 | DAGPPGPAGPAGP | Negative |
| P05997 | 173 | PGQPGAPGPPGHP | Negative |
| P08123 | 1032 | NGLQGLPGIAGHH | Negative |
| P04925 | 59 | GGTWGQPHGGGWG | Negative |
| Q9M0S4 | 40 | PSPAQPPRTAAPT | Negative |
| P05997 | 239 | AGPTGPPGEPGDP | Negative |
| Q02388 | 1524 | KGPEGPPGPTGRQ | Negative |
| P12111 | 2321 | FPGYPGPKGNPGE | Negative |
| Q9UKV8 | 7 | MYSGAGPALAPPA | Negative |
| O97939 | 145 | PLNEPSPTPTQPE | Negative |
| Q15485 | 128 | YLPDCRPLTVLCD | Negative |
| P12111 | 1426 | LASTRYPPPAVES | Negative |
| P05997 | 566 | PGRPGEPGLPGAR | Negative |
| P12108 | 477 | ELGFPGPSGDAGS | Negative |
| P12108 | 484 | SGDAGSPGVRGYP | Negative |
| P02453 | 135 | PGRDGIPGQPGLP | Negative |
| Q05707 | 1009 | EKTQSLPTRPPTF | Negative |
| Q9UKV8 | 229 | AFYKAQPVIEFVC | Negative |
| P15502 | 546 | PGVGVAPGIGPGG | Negative |
| P02453 | 566 | QDGRPGPPGPPGA | Negative |
| Q05707 | 1588 | ITGSMGPQGALGP | Negative |
| P85886 | 62 | TCRMEYPELCGGR | Negative |
| P12108 | 26 | LAQLRGPPGEPGP | Negative |
| Q02388 | 2691 | RGFDGQPGPKGDQ | Negative |
| P08252 | 185 | GQWPCAPGRKYFG | Negative |
| P02453 | 204 | QGFQGPPGEPGEP | Negative |
| P35248 | 68 | KGDPGLPGPMGLS | Negative |
| P08123 | 902 | PLGIAGPPGARGP | Negative |
| Q02388 | 1472 | EQGPRGPPGAIGP | Negative |
| Q28084 | 327 | SYWLSTPAMIPMD | Negative |
| Q02388 | 891 | LRWEPVPRAQGFL | Negative |
| P12111 | 1226 | LQPLPSPGVGGKR | Negative |
| P02745 | 125 | AIRRNPPMGGNVV | Negative |
| Q7XAD0 | 98 | TTITTTPHHDDTV | Negative |
| Q15485 | 88 | KAGPPGPNGAPGE | Negative |
| P35248 | 110 | VGPPGSPGISGPA | Negative |
| Q557E4 | 42 | ESDAPIPLPNVTS | Negative |
| P08123 | 651 | RGAAGIPGGKGEK | Negative |
| Q02388 | 1220 | FAVDDGPSLDQAV | Negative |
| O97939 | 182 | HVPHRIPPGYGRP | Negative |
| Q24940 | 320 | ASLASLPMVARFP | Negative |
| Q9SJY7 | 75 | TSPVASPPQTDAP | Negative |
| P08661 | 209 | NWNEGEPNNVGSG | Negative |
| P02453 | 113 | TTGVEGPKGDTGP | Negative |
| P05997 | 154 | PKGRPGPRGPQGI | Negative |
| P12108 | 641 | DGERGPPGVPGDA | Negative |
| Q25460 | 871 | YPPAYKPTNRY-- | Negative |
| P20908 | 471 | MLIEGPPGPEGPA | Negative |
| Q02388 | 1158 | GRRQHVPGVMVLL | Negative |
| P08123 | 456 | RGLPGSPGNIGPA | Negative |
| P00877 | 410 | GGTLGHPWGNAPG | Negative |
| Q25460 | 503 | PKKTYPPTYKPKL | Negative |
| P02453 | 896 | NAGPPGPPGPAGK | Negative |
| O75636 | 118 | PEGRALPVFCDMD | Negative |
| P05997 | 1183 | PPGPVGPSGKEGN | Negative |
| Q05707 | 110 | KDKESKPAQGQFR | Negative |
| P05997 | 850 | AVGFAGPQGPDGQ | Negative |
| O97939 | 719 | EFYPWNPEENFPS | Negative |
| Q02388 | 889 | LRLRWEPVPRAQG | Negative |
| Q02388 | 2438 | REGIPGPLGPPGP | Negative |
| Q05707 | 1749 | HLGVPGPQGPSGQ | Negative |
| P20908 | 1452 | VGEQGLPGSPGPD | Negative |
| O75636 | 73 | PPGKMGPKGEPGD | Negative |
| P83455 | 60 | KPPAWVPGK---- | Negative |
| P02453 | 1317 | WYISKNPKEKRHV | Negative |
| P02453 | 1050 | PGAPGAPGAPGPV | Negative |
| P12111 | 2366 | YPGPAGPKGNRGD | Negative |
| P01523 | 27 | PMDGDEPANRPVE | Negative |
| P08125 | 264 | KPGPMGPPGPAGI | Negative |
| Q8LG54 | 41 | PPVATPPPAATPA | Negative |
| Q9UKV8 | 731 | GKSGNIPAGTTVD | Negative |
| P69929 | 38 | EPNVAVPPCGDCY | Negative |
| O97939 | 12 | RHGASSPKLDNLV | Negative |
| P20908 | 389 | TSNSSNPAPPPGE | Negative |
| P20908 | 1575 | PGEVIQPLPIQAS | Negative |
| P08125 | 434 | INGEPGPRGPSGI | Negative |
| P05997 | 757 | TPGDTGPPGLQGM | Negative |
| P02453 | 591 | KGAAGEPGKAGER | Negative |
| Q02388 | 2869 | YQDPEAPWDSDDP | Negative |
| O97939 | 1030 | PDKEPRPEGIPNE | Negative |
| P08123 | 396 | AGPPGPPGLRGSP | Negative |
| Q02388 | 1704 | EPGPPGPPGRLVD | Negative |
| Q24940 | 326 | PMVARFP------ | Negative |
| Q05707 | 1655 | IRTVQGPPGEPGR | Negative |
| P08123 | 951 | KGERGYPGNIGPV | Negative |
| P02453 | 695 | PPGPAGPRGANGA | Negative |
| P24091 | 56 | TNDYCGPGNCQSQ | Negative |
| P12111 | 2280 | EPGEPGPKGGIGN | Negative |
| P08125 | 63 | PPGPPGPIGPRGQ | Negative |
| O97939 | 627 | PNTMGQPENPHYP | Negative |
| Q05707 | 444 | ETTLALPMASDLL | Negative |
| P12111 | 2954 | KPAAVRPPAAAAA | Negative |
| Q02388 | 1732 | QEGPRGPKGDPGL | Negative |
| P69929 | 195 | RVPSLCPSRKREP | Negative |
| P05997 | 538 | SDGLPGPKGAQGE | Negative |
| Q02388 | 2277 | RGSPGVPGSPGLP | Negative |
| P12111 | 1629 | PGVDTPPPSRPEK | Negative |
| P02746 | 163 | KFTCKVPGLYYFT | Negative |
| Q9C5S0 | 95 | PVASPPPPVASPP | Negative |
| P69765 | 10 | LLITSTPSVDARL | Negative |
| P02453 | 82 | DELKDCPNAKVPT | Negative |
| P20908 | 513 | PGADGLPGPPGTM | Negative |
| P08123 | 1212 | AQPENIPAKNWYR | Negative |
| P02453 | 153 | PGPPGPPGLGGNF | Negative |
| O97939 | 644 | DPKETIPYNEEDP | Negative |
| Q16665 | 702 | SQRTTVPEEELNP | Negative |
| Q25460 | 50 | SKTNYLPLAKKLS | Negative |
| O97939 | 568 | NQQEISPPFKEDP | Negative |
| P12111 | 87 | VQFNGNPHTEFLL | Negative |
| P05997 | 1157 | QGLPGPPGPNGEQ | Negative |
| P12111 | 550 | RAAEGIPKLLVLI | Negative |
| P20908 | 15 | ARSALRPGAPLLP | Negative |
| P05997 | 619 | SMGLPGPKGSSGD | Negative |
| P12108 | 313 | DGTPGIPGVKGTA | Negative |
| P84900 | 58 | RPPGFSPFRIY-- | Negative |
| P12108 | 393 | DRGERGPVGAPGE | Negative |
| Q05707 | 800 | LLKPLLPDTEYKV | Negative |
| P08123 | 134 | PAGARGPAGPPGK | Negative |
| Q02388 | 725 | WHSAHGPEKSQLV | Negative |
| Q25460 | 345 | PTYKAKPTYKAKP | Negative |
| P30754 | 74 | RDGEPGPRGGIGP | Negative |
| P02453 | 342 | TGPAGPPGFPGAV | Negative |
| P02453 | 458 | PTGIQGPPGPAGE | Negative |
| P15502 | 729 | GGLGGIPPAAAAK | Negative |
| P08125 | 505 | LPGPPGPPGPPGQ | Negative |
| P20908 | 1530 | SGPIGPPGPPGLP | Negative |
| P86289 | 106 | PAGPAGPPGPAGA | Negative |
| P05997 | 1154 | TGLQGLPGPPGPN | Negative |
| P02453 | 1185 | PGPPGPPGPPGPP | Negative |
| P12111 | 290 | VQFSDEPRTMFSL | Negative |
| P20908 | 855 | GGPNGDPGPLGPP | Negative |
| P02453 | 210 | PGEPGEPGASGPM | Negative |
| P05997 | 536 | PGSDGLPGPKGAQ | Negative |
| P23805 | 42 | TLVMCSPLESGLP | Negative |
| P08123 | 995 | PAGAVGPRGPSGP | Negative |
| P12108 | 328 | PGRPGPPGHRGQA | Negative |
| Q9SJY7 | 71 | TSPTTSPVASPPQ | Negative |
| O97939 | 188 | PPGYGRPPTSNEE | Negative |
| Q02388 | 2598 | AGIPGDPGSPGKD | Negative |
| P08123 | 68 | PGPPGPPGPPGLG | Negative |
| O97939 | 369 | GKQAVRPGYPTYR | Negative |
| P20908 | 1508 | DRGLPGPQGSSGP | Negative |
| Q25460 | 847 | SKTSYPPTYNKKI | Negative |
| P08125 | 93 | PGPLGPPGFSTVG | Negative |
| P05997 | 409 | QRGETGPPGPVGS | Negative |
| Q02388 | 2196 | APGLAGPAGPQGP | Negative |
| Q9U3Z3 | 57 | GHPCRHPGKRSKL | Negative |
| P12108 | 460 | KGESGEPGPKGQQ | Negative |
| Q9STQ3 | 44 | DASLAIPAFFASV | Negative |
| P12111 | 2141 | RRGDKGPRGEKGE | Negative |
| P20908 | 1478 | LKGDSGPKGEKGH | Negative |
| Q02388 | 1176 | RGDIFSPIREAQA | Negative |
| Q05707 | 646 | FRVTWHPLSADEG | Negative |
| Q02388 | 1473 | QGPRGPPGAIGPK | Negative |
| P20908 | 1178 | DKGEQGPPGPTGP | Negative |
| P20908 | 1293 | PGEAGEPGLPGEG | Negative |
| Q02388 | 1611 | PPGDSGPPGEKGD | Negative |
| P12111 | 2336 | LNGTTGPKGIRGR | Negative |
| P15502 | 203 | GPQPGVPLGYPIK | Negative |
| P20908 | 1431 | IGPQGAPGKPGPD | Negative |
| P08123 | 1076 | HPGTVGPAGIRGP | Negative |
| Q16665 | 461 | TAETPKPLRSSAD | Negative |
| P08123 | 552 | QGPAGPPGFQGLP | Negative |
| P04640 | 25 | DLAGAKPSDSESD | Negative |
| P12111 | 2191 | DGVPGGPGETGKN | Negative |
| Q02388 | 1753 | IEGFRGPPGPQGD | Negative |
| P12108 | 562 | PGPPGPPGPPGEQ | Negative |
| B2KPN7 | 17 | IFLVLFPMATLQL | Negative |
| P0C1N1 | 21 | FPLTAVPLDGDQP | Negative |
| Q557E4 | 128 | MIRGKTPEEIRKI | Negative |
| P02453 | 1017 | SGREGAPGAEGSP | Negative |
| Q02388 | 235 | DDSTSAPRDLVLS | Negative |
| P20908 | 1083 | EGPPGPPGPAGSP | Negative |
| P08123 | 278 | NAGPAGPAGPRGE | Negative |
| P08125 | 308 | HRGPEGPPGFPGP | Negative |
| Q02388 | 2350 | AGRAGEPGDPGED | Negative |
| P20908 | 1394 | PPGKRGPPGPAGP | Negative |
| P20908 | 92 | PTKQLYPASAFPE | Negative |
| Q02388 | 180 | GIKNADPEELKRV | Negative |
| P12111 | 1246 | GSQSAGPEFQYVR | Negative |
| O97939 | 848 | AFPDLIPPDYPGG | Negative |
| P08125 | 557 | ILSKAYPGATVPI | Negative |
| Q9UKV8 | 67 | IKPEKCPRRVNRE | Negative |
| P20908 | 1434 | QGAPGKPGPDGLR | Negative |
| P05997 | 752 | PGPSGTPGDTGPP | Negative |
| P02747 | 147 | NAVLTNPQGDYDT | Negative |
| Q02388 | 1461 | PGLPGQPGSPGEQ | Negative |
| P20908 | 242 | YCEHYSPDCDTAV | Negative |
| Q9C5S0 | 113 | PVATPPPAPLASP | Negative |
| Q28084 | 226 | PGSDGLPGLKGKP | Negative |
| P84349 | 78 | EVCELNPDCDELA | Negative |
| Q25460 | 39 | RPTKSYPPTYGSK | Negative |
| Q02388 | 2712 | PGIGGFPGPSGND | Negative |
| P15502 | 324 | AAAGLVPGGPGFG | Negative |
| Q02388 | 1381 | PPGPRGPLGDPGP | Negative |
| P84900 | 50 | EKKRESPDRPPGF | Negative |
| Q02388 | 2543 | DMGERGPRGLDGD | Negative |
| P20908 | 1059 | PGLRGFPGDRGLP | Negative |
| Q9XF04 | 89 | VNPPRQPRNNFQL | Negative |
| P01523 | 45 | ISSEQYPLFEKRR | Negative |
| P12111 | 959 | SDRVDGPASNLKQ | Negative |
| Q25460 | 806 | KPKLTYPPTYKPK | Negative |
| P05997 | 449 | PGSAGPPGSPGPQ | Negative |
| P02453 | 1119 | SGLQGPPGPPGSP | Negative |
| P01042 | 260 | KDFVQPPTKICVG | Negative |
| P08123 | 704 | AAGPAGPAGPRGS | Negative |
| P20908 | 1082 | NEGPPGPPGPAGS | Negative |
| P05997 | 1058 | AGNDGTPGRDGAV | Negative |
| Q05707 | 355 | AHAITGPPTELIT | Negative |
| Q16665 | 805 | DCEVNAPIQGSRN | Negative |
| P58786 | 28 | QGDGDQPADRNAV | Negative |
| Q02388 | 1499 | EKGERGPPGPAGS | Negative |
| P20908 | 941 | NSGGDGPAGPPGE | Negative |
| P12111 | 3103 | LMVSTEPLALTET | Negative |
| Q02388 | 2092 | PPGPPGPKVSVDE | Negative |
| P08123 | 1092 | QGPAGPPGPPGPP | Negative |
| P20908 | 448 | YEGIGGPRGEKGQ | Negative |
| P02820 | 64 | GAPAPYPDPLEPK | Negative |
| P08252 | 244 | TPQSPKPSCHDVI | Negative |
| P30754 | 629 | ERGSPGPAGERGP | Negative |
| P02453 | 804 | PGDRGEPGPPGPA | Negative |
| Q25460 | 826 | KPKITYPSTYKLK | Negative |
| Q02388 | 2101 | SVDEPGPGLSGEQ | Negative |
| P05997 | 442 | SPGTSGPPGSAGP | Negative |
| P08125 | 399 | KGHPGLPGQKGDT | Negative |
| P02453 | 1277 | GEYWIDPNQGCNL | Negative |
| P08123 | 686 | AVGAPGPAGATGD | Negative |
| Q9GQV7 | 111 | RSAESEPFVTRIR | Negative |
| Q9ZT16 | 103 | PSSAPGPSDASPA | Negative |
| Q16665 | 332 | NTKNSQPQCIVCV | Negative |
| Q9UKV8 | 416 | GRVLQPPSILYGG | Negative |
| P20908 | 1325 | AAGPPGPKGPPGD | Negative |
| O97939 | 942 | PGESQNPSPFKDD | Negative |
| P02453 | 503 | ADGVAGPKGPAGE | Negative |
| P20908 | 1191 | QGPIGQPGPSGAD | Negative |
| P20908 | 31 | LLLLWAPPPSRAA | Negative |
| Q28084 | 132 | FPGPPGPPGQSGP | Negative |
| P20908 | 52 | LDFHNLPDGITKT | Negative |
| O97939 | 1130 | LQIGTNPQDQVQD | Negative |
| P08123 | 138 | RGPAGPPGKAGED | Negative |
| P69765 | 46 | INTRCCPGKPCCR | Negative |
| P08125 | 259 | EVGIGKPGPMGPP | Negative |
| P08123 | 963 | VGAAGAPGPHGPV | Negative |
| O97939 | 400 | ANLRRKPEGPNKN | Negative |
| Q02388 | 1906 | QGFPGVPGGTGPK | Negative |
| P20908 | 470 | GMLIEGPPGPEGP | Negative |
| P08125 | 578 | RQQHYDPRTGIFT | Negative |
| P12111 | 618 | PLQGMLPGLLAPL | Negative |
| P12108 | 57 | KGSPGAPGSPGAK | Negative |
| P12111 | 223 | VHSSVSPERAGDT | Negative |
| P08125 | 221 | PGENGLPGQPGMK | Negative |
| P02746 | 92 | KVGPKGPMGPKGG | Negative |
| Q941C7 | 60 | ANPKHDPGVPPSA | Negative |
| P12108 | 340 | AGLPGQPGSKGGP | Negative |
| P02453 | 197 | PPGAPGPQGFQGP | Negative |
| O97939 | 212 | HGFGGRPPYYSEE | Negative |
| P02453 | 284 | DAGPAGPKGEPGS | Negative |
| Q05707 | 1594 | PQGALGPPGVPGA | Negative |
| P02453 | 869 | ARGSAGPPGATGF | Negative |
| Q9UKV8 | 762 | IQGTSRPSHYHVL | Negative |
| Q02388 | 1585 | LVLPGDPGPKGDP | Negative |
| P08125 | 72 | PRGQPGPAGKPGF | Negative |
| Q02388 | 2384 | SPGPPGPPGVKGD | Negative |
| P02453 | 144 | PGLPGPPGPPGPP | Negative |
| P05997 | 806 | PGPLGPPGPAGPT | Negative |
| P84349 | 25 | GQAGAKPSGAESS | Negative |
| P08125 | 449 | VRGPIGPPGMPGA | Negative |
| Q9Y2N7 | 592 | LLGVRPPKRSPSP | Negative |
| Q8LG54 | 90 | EGPGVSPGELAPT | Negative |
| P86289 | 74 | PSGPPGPTGARGS | Negative |
| P24091 | 172 | PGDYCTPSGQWPC | Negative |
| Q28084 | 402 | GQALASPGSCLEE | Negative |
| Q9ZT16 | 83 | PSPADVPTASPPA | Negative |
| P05997 | 701 | QGVPGDPGAVGPL | Negative |
| P01042 | 627 | SVSEINPTTQMKE | Negative |
| Q02388 | 2468 | DPGVGLPGPRGER | Negative |
| Q02388 | 1705 | PGPPGPPGRLVDT | Negative |
| P08123 | 297 | SGPVGPPGNPGAN | Negative |
| P02453 | 518 | APGPAGPKGSPGE | Negative |
| P58925 | 45 | ISSEEHPFEKRQR | Negative |
| P20908 | 1236 | QGLPGPPGEKGET | Negative |
| P12108 | 495 | YPGPPGPRGLLGE | Negative |
| Q02388 | 863 | SIVVTTPPEAPPA | Negative |
| Q557E4 | 40 | IGESDAPIPLPNV | Negative |
| P12111 | 21 | LFLSGFPTTHAQQ | Negative |
| P08123 | 255 | IGSAGPPGFPGAP | Negative |
| P08123 | 548 | GKGEQGPAGPPGF | Negative |
| P02746 | 9 | IPWGSIPVLMLLL | Negative |
| O97939 | 549 | TEGIPSPAKEHFP | Negative |
| P05997 | 349 | ERGRLGPQGAPGQ | Negative |
| Q9M0S4 | 39 | LPSPAQPPRTAAP | Negative |
| P04640 | 76 | EVCELNPNCDELA | Negative |
| Q02388 | 2706 | KGERGTPGIGGFP | Negative |
| Q60994 | 107 | PGRKGEPGEAAYM | Negative |
| Q9UKV8 | 430 | NKAIATPVQGVWD | Negative |
| Q93WP7 | 41 | GRGTNLPPPSPAS | Negative |
| O97939 | 471 | NYKLPQPEDNMLV | Negative |
| Q02388 | 675 | RGREEGPAAVIVA | Negative |
| P20908 | 1113 | PGPQGPPGPAGEK | Negative |
| Q02388 | 2155 | DGNPGLPGERGMA | Negative |
| Q15485 | 56 | RGCPGLPGAPGPK | Negative |
| P02453 | 513 | AGERGAPGPAGPK | Negative |
| O97939 | 1086 | DSFDGDPIMPTET | Negative |
| P15502 | 475 | AAVPGVPGTGGVP | Negative |
| P05997 | 1217 | EGPPGEPGPPGPP | Negative |
| P08123 | 522 | AGARGAPGPDGNN | Negative |
| P00877 | 142 | EDLRIPPAYVKTF | Negative |
| Q02388 | 1168 | VLLVDEPLRGDIF | Negative |
| Q3Y5Z3 | 57 | DGRDGTPGEKGEK | Negative |
| P05997 | 524 | VGERGAPGNRGFP | Negative |
| Q25460 | 133 | YPPTYKPKLTYPP | Negative |
| P58808 | 31 | KTEDDVPMSSVYG | Negative |
| P12111 | 2348 | RRGNSGPPGIVGQ | Negative |
| P02453 | 845 | PPGPAGPAGPPGP | Negative |
| P12111 | 1162 | KRGGAVPIGIGIG | Negative |
| O97939 | 920 | ARQTVSPTSIVPG | Negative |
| P08123 | 296 | LSGPVGPPGNPGA | Negative |
| P08123 | 258 | AGPPGFPGAPGPK | Negative |
| P02453 | 195 | PGPPGAPGPQGFQ | Negative |
| Q02388 | 2163 | ERGMAGPEGKPGL | Negative |
| P08125 | 271 | PGPAGIPGAKGLP | Negative |
| P23805 | 71 | DPGSPGPAGRAGR | Negative |
| Q9C5S0 | 70 | APPPANPPPPVSS | Negative |
| P08123 | 719 | ERGEVGPAGPNGF | Negative |
| O97939 | 724 | NPEENFPSYNTAP | Negative |
| P20908 | 18 | ALRPGAPLLPPLL | Negative |
| P12111 | 568 | LDEISQPAQELKR | Negative |
| Q25460 | 771 | YPPTYKPKPSYPP | Negative |
| P02453 | 570 | PGPPGPPGARGQA | Negative |
| Q28084 | 27 | TGPQGAPGFPGVT | Negative |
| P86289 | 38 | PKGVQGPPGPQGP | Negative |
| Q02388 | 2057 | VGEAGRPGERGER | Negative |
| P30754 | 983 | GRGSQGPPGKDGQ | Negative |
| Q05707 | 855 | WDPPSSPVKGYRI | Negative |
| P05997 | 410 | RGETGPPGPVGSP | Negative |
| Q9Y2N7 | 434 | AATPSTPLATRHP | Negative |
| O97939 | 242 | ETPATEPSVNTTV | Negative |
| P02453 | 1139 | ASGPAGPRGPPGS | Negative |
| P02453 | 425 | PQGPSGPPGPKGN | Negative |
| Q02388 | 1880 | KGERGAPGILGPQ | Negative |
| P08123 | 1130 | SAPSLRPKDYEVD | Negative |
| Q02388 | 1582 | PPGLVLPGDPGPK | Negative |
| Q02388 | 991 | YILSWRPLRGPGQ | Negative |
| P12108 | 149 | DGPRGPPGPPGKP | Negative |
| P05997 | 853 | FAGPQGPDGQPGV | Negative |
| P08427 | 195 | MIEDQTPGDFHYL | Negative |
| P02453 | 894 | SGNAGPPGPPGPA | Negative |
| Q02388 | 2081 | DGPPGLPGTPGPP | Negative |
| Q25460 | 143 | YPPTYKPKPSYPP | Negative |
| Q02388 | 2086 | LPGTPGPPGPPGP | Negative |
| Q05707 | 206 | AQYSGDPRIEWHL | Negative |
| P08123 | 848 | FAGEKGPSGEAGT | Negative |
| Q16665 | 644 | LIASPSPTHIHKE | Negative |
| Q02388 | 1455 | DSEDGAPGLPGQP | Negative |
| O97939 | 555 | PAKEHFPAGRNTW | Negative |
| P00877 | 372 | QDWCSMPGVMPVA | Negative |
| Q02388 | 2013 | DRGDPGPQGPPGL | Negative |
| P35248 | 97 | SAGEPGPKGERGL | Negative |
| P23805 | 69 | KGDPGSPGPAGRA | Negative |
| P15502 | 93 | FPGALVPGGVADA | Negative |
| P20908 | 1251 | VGQMGPPGPPGPR | Negative |
| P08125 | 446 | IPGVRGPIGPPGM | Negative |
| P12111 | 3014 | HWERPEPPGPYFY | Negative |
| Q02388 | 1000 | GPGQEVPGSPQTL | Negative |
| P24091 | 64 | NCQSQCPGGPTPP | Negative |
| P12111 | 2235 | AQGPAGPAGPPGL | Negative |
| P05997 | 965 | AGPPGGPGDKGDP | Negative |
| P35248 | 41 | TLVLCSPTENGLP | Negative |
| Q9Y2N7 | 655 | DEDTTQPGGPFQP | Negative |
| Q28084 | 94 | MGSPGHPGAPGVP | Negative |
| O97939 | 331 | ARRQWRPPGPAMG | Negative |
| Q9UKV8 | 484 | SRDAGMPIQGQPC | Negative |
| P05997 | 1051 | EPGPEGPAGNDGT | Negative |
| P35248 | 290 | GGQLASPRSATEN | Negative |
| P05997 | 670 | ERGEQGPPGPTGF | Negative |
| P20908 | 860 | DPGPLGPPGEKGK | Negative |
| P15502 | 198 | VGPFGGPQPGVPL | Negative |
| Q9Y2N7 | 420 | LRRLLGPILDGAS | Negative |
| P08123 | 242 | SDGSVGPVGPAGP | Negative |
| Q9M0S4 | 83 | SASPPAPPTSLTP | Negative |
| P15502 | 161 | LPGARFPGVGVLP | Negative |
| P35248 | 272 | AANSEEPFEDAKE | Negative |
| P20908 | 965 | FPGPKGPPGPPGK | Negative |
| P12108 | 173 | SADFLCPTNCPPG | Negative |
| P58846 | 21 | FSLNAVPLDGDQP | Negative |
| Q02388 | 2681 | KEGLIGPKGDRGF | Negative |
| O97939 | 256 | ETNSTQPNAPNPR | Negative |
| P05997 | 212 | SQVGLMPGSVGPV | Negative |
| Q557E4 | 94 | FCKVDQPTLFELI | Negative |
| P02453 | 1142 | PAGPRGPPGSAGS | Negative |
| P08123 | 1044 | HGDQGAPGSVGPA | Negative |
| P12111 | 2866 | NNVTSSPTSNPVT | Negative |
| Q02388 | 456 | RETGLEPPQKVVL | Negative |
| P08123 | 971 | PHGPVGPAGKHGN | Negative |
| Q28084 | 141 | QSGPKGPPGVRGE | Negative |
| Q02388 | 2187 | GHGDPGPPGAPGL | Negative |
| Q9UKV8 | 523 | LVVVILPGKTPVY | Negative |
| Q02388 | 1564 | DVGPAGPRGATGV | Negative |
| P05997 | 341 | LGPRGMPGERGRL | Negative |
| P19999 | 5 | #NAME? | Negative |
| Q28084 | 133 | PGPPGPPGQSGPK | Negative |
| P20908 | 1334 | PPGDDGPKGSPGP | Negative |
| P12108 | 142 | PKGDPGPDGPRGP | Negative |
| P02745 | 115 | IKDQPRPAFSAIR | Negative |
| Q02388 | 2486 | RGEDGRPGQEGPR | Negative |
| Q9GQV7 | 82 | KRSAQRPPSLKTR | Negative |
| P08123 | 98 | PMGLMGPRGPPGA | Negative |
| P08123 | 593 | PRGERGPPGESGA | Negative |
| Q8LG54 | 81 | APTASPPAPEGPG | Negative |
| Q16665 | 793 | MDESGLPQLTSYD | Negative |
| O97939 | 238 | PPKTETPATEPSV | Negative |
| Q28084 | 81 | EPGQRGPPGAIGD | Negative |
| O97939 | 139 | TPTPKQPLNEPSP | Negative |
| P08123 | 758 | VVGPTGPVGAAGP | Negative |
| P12108 | 223 | SGEQGVPGPPGPQ | Negative |
| O97939 | 637 | HYPMNTPDPKETI | Negative |
| Q9Y2N7 | 596 | RPPKRSPSPEHEN | Negative |
| P05997 | 473 | PGDPGVPGFKGEA | Negative |
| Q9Y2N7 | 541 | LEPSLLPRWGSDP | Negative |
| Q02388 | 2084 | PGLPGTPGPPGPP | Negative |
| Q02388 | 1651 | PGDPGLPGKAGER | Negative |
| Q9UKV8 | 25 | QGYAFKPPPRPDF | Negative |
| P02453 | 177 | STGISVPGPMGPS | Negative |
| Q9UKV8 | 29 | FKPPPRPDFGTSG | Negative |
| P05997 | 958 | RVGDRGPAGPPGG | Negative |
| Q9Y2N7 | 377 | QKDTPNPGDSLDT | Negative |
| Q9UKV8 | 557 | NVQRTTPQTLSNL | Negative |
| P12108 | 69 | KGEPGAPGPDGPP | Negative |
| P12111 | 2178 | ETGDLGPMGVPGR | Negative |
| P20908 | 1104 | AGPIGIPGRPGPQ | Negative |
| P15502 | 200 | PFGGPQPGVPLGY | Negative |
| Q05707 | 1466 | ALGPAGPPGGPGL | Negative |
| P12108 | 235 | QGQRGYPGMAGPK | Negative |
| Q02388 | 2232 | VGLPGPPGPSGLV | Negative |
| P02453 | 1047 | AGPPGAPGAPGAP | Negative |
| P15502 | 763 | GGVAARPGFGLSP | Negative |
| Q02388 | 2595 | PGAAGIPGDPGSP | Negative |
| Q02388 | 1729 | DRGQEGPRGPKGD | Negative |
| P05997 | 979 | EDGQPGPDGPPGP | Negative |
| P20908 | 978 | DGLPGHPGQRGET | Negative |
| Q9Y2N7 | 559 | SPSRGDPSASSPM | Negative |
| P08252 | 182 | TPSGQWPCAPGRK | Negative |
| P12108 | 152 | RGPPGPPGKPGPP | Negative |
| P02453 | 860 | NVGAPGPKGARGS | Negative |
| Q02388 | 2011 | KGDRGDPGPQGPP | Negative |
| P12108 | 78 | DGPPGKPGLDGLT | Negative |
| P02453 | 248 | ERGPPGPQGARGL | Negative |
| P08123 | 533 | NNGAQGPPGPQGV | Negative |
| Q16665 | 482 | LKLEPNPESLELS | Negative |
| Q02388 | 2294 | PKGEPGPTGAPGQ | Negative |
| P05997 | 10 | NWAEARPLLILIV | Negative |
| P08125 | 351 | PGENGLPGPKGDM | Negative |
| O97939 | 735 | APTVSSPVESRGY | Negative |
| Q16665 | 775 | KTIILIPSDLACR | Negative |
| P01042 | 554 | IPSLAKPGVTVTF | Negative |
| P02453 | 1070 | DRGETGPAGPAGP | Negative |
| P12111 | 2876 | PVTTTKPVTTTKP | Negative |
| P08123 | 806 | PSGISGPPGPPGP | Negative |
| P02453 | 428 | PSGPPGPKGNSGE | Negative |
| Q02388 | 1940 | GEPGSVPNVDRLL | Negative |
| P02453 | 651 | PGPAGPPGEAGKP | Negative |
| P02453 | 1158 | DGLNGLPGPIGPP | Negative |
| Q25460 | 168 | KTKKTYPSSYKAK | Negative |
| P12111 | 2540 | SDAGITPLFLTRQ | Negative |
| P35248 | 341 | NWAPGEPNNNGGA | Negative |
| P20908 | 147 | EDHTGKPGPEDYP | Negative |
| P02453 | 1040 | DRGETGPAGPPGA | Negative |
| Q9C5S0 | 59 | PVTTSPPPVTTAP | Negative |
| P02746 | 47 | IPGTPGPDGQPGT | Negative |
| P20908 | 1001 | PPGVVGPQGPTGE | Negative |
| P84349 | 62 | WLGAPVPYPDPLE | Negative |
| P12111 | 631 | RTLSGTPEVHSNK | Negative |
| P20908 | 1419 | AGLEGPPGKTGPI | Negative |
| Q02388 | 1618 | PGEKGDPGRPGPP | Negative |
| Q02388 | 995 | WRPLRGPGQEVPG | Negative |
| P05997 | 367 | MPGKPGPMGPLGI | Negative |
| P08123 | 584 | EFGLPGPAGPRGE | Negative |
| P29602 | 74 | DVERTSPVIERLD | Negative |
| Q9ZT16 | 60 | TPAPATPPPAATP | Negative |
| P52285 | 67 | HHQHPSPQGDDKK | Negative |
| Q28084 | 127 | KGNSGFPGPPGPP | Negative |
| O97939 | 696 | QPKEYLPYSLDNP | Negative |
| P08125 | 58 | RGEQGPPGPPGPI | Negative |
| Q05707 | 1737 | PPGSPGPRGPPGH | Negative |
| P69929 | 140 | EPNVAVPPCGDCY | Negative |
| Q02388 | 2815 | IASGSRPLPSYAA | Negative |
| P05997 | 1126 | LPGPQGPRGDKGD | Negative |
| Q02388 | 1760 | PGPQGDPGVRGPA | Negative |
| P08123 | 684 | PGAVGAPGPAGAT | Negative |
| P08125 | 465 | KGEAGAPGLPGPA | Negative |
| P30754 | 95 | MPGMPGPKGHRGF | Negative |
| P05997 | 131 | TGIRGRPGPAGPP | Negative |
| P02453 | 1204 | SFLPQPPQEKAHD | Negative |
| P12108 | 310 | DGKDGTPGIPGVK | Negative |
| P05997 | 412 | ETGPPGPVGSPGL | Negative |
| Q02388 | 581 | VSARVGPREGSAS | Negative |
| Q9Y2N7 | 375 | PSQKDTPNPGDSL | Negative |
| Q02388 | 1889 | LGPQGPPGLPGPV | Negative |
| P08123 | 840 | VGAVGPPGFAGEK | Negative |
| P04640 | 67 | YPDPLEPHREVCE | Negative |
| P12111 | 2912 | KPAAAKPAPAKPV | Negative |
| Q9C5S0 | 137 | KPDSPSPSPSSSP | Negative |
| O97939 | 268 | RGNDTSPTGTSGQ | Negative |
| Q28084 | 186 | PPGDPGPCGPKGK | Negative |
| P02453 | 506 | VAGPKGPAGERGA | Negative |
| Q25460 | 653 | YPPTYKPKISYPP | Negative |
| P12111 | 1366 | KQFGVAPFTIARN | Negative |
| P05997 | 692 | PGEGGKPGDQGVP | Negative |
| Q9Y2N7 | 520 | RPLGAVPRPRARS | Negative |
| P12108 | 262 | IGAAGRPGREGPK | Negative |
| P05997 | 877 | DAGSPGPQGLAGS | Negative |
| Q16665 | 741 | GTLLQQPDDHAAT | Negative |
| P05997 | 775 | IAGTPGPKGDRGG | Negative |
| P02453 | 359 | EGGPQGPRGSEGP | Negative |
| Q02388 | 2045 | PGIPGLPGRAGGV | Negative |
| P12111 | 3081 | STKKSQPPPPQPA | Negative |
| P20908 | 585 | GGLKGEPGDVGPQ | Negative |
| Q9C5S0 | 34 | TATPAPPTPTTPP | Negative |
| Q05707 | 1570 | PPGPPGPIGIPGT | Negative |
| P08125 | 502 | EPGLPGPPGPPGP | Negative |
| P20908 | 1151 | PAGPVGPPGEDGD | Negative |
| P08125 | 165 | AQGPRGPPGEKGE | Negative |
| P19999 | 137 | TNHERMPFSKVKA | Negative |
| P08123 | 120 | QGPAGEPGEPGQT | Negative |
| P05997 | 190 | PDGLSRPFSAQMA | Negative |
| P08123 | 1362 | FFVDIGPVCFK-- | Negative |
| Q02388 | 2727 | AGPPGPPGSVGPR | Negative |
| P86289 | 44 | PPGPQGPRGLTGP | Negative |
| Q02388 | 2215 | PGETGPPGRGLTG | Negative |
| Q9UKV8 | 584 | LLPQGRPPVFQQP | Negative |
| P08123 | 348 | RGLVGEPGPAGSK | Negative |
| P08123 | 449 | EPGLMGPRGLPGS | Negative |
| Q02388 | 286 | RQEVNVPAGETSV | Negative |
| P20908 | 501 | PGERGPPGRPGLP | Negative |
| P12111 | 2967 | KPVATKPEVPRPQ | Negative |
| P04640 | 64 | PAPYPDPLEPHRE | Negative |
| P20908 | 1389 | PGPSGPPGKRGPP | Negative |
| Q9ZT16 | 87 | DVPTASPPAPEGP | Negative |
| P02746 | 89 | NPGKVGPKGPMGP | Negative |
| Q28084 | 157 | VKIISLPGSPGPP | Negative |
| Q9C5S0 | 65 | PPVTTAPPPANPP | Negative |
| P23805 | 98 | FVGEPGPKGDTGP | Negative |
| Q9C5S0 | 39 | PPTPTTPPPAATP | Negative |
| P12108 | 32 | PPGEPGPRGPPGP | Negative |
| P02453 | 1188 | PGPPGPPGPPSGG | Negative |
| Q9Y2N7 | 598 | PKRSPSPEHENFL | Negative |
| Q02388 | 1391 | PGPRGPPGLPGTA | Negative |
| P42849 | 80 | MLNTTGPQYYICT | Negative |
| P05997 | 325 | SKGEAGPTGPMGA | Negative |
| P58846 | 49 | NNPFYDPAKRCCR | Negative |
| P12111 | 2070 | PGDEGGPGERGPP | Negative |
| Q05707 | 395 | PTRGGKPDEVVVD | Negative |
| P30754 | 818 | DGGPPGPSGDRGE | Negative |
| O97939 | 523 | LEPRRIPYESETN | Negative |
| Q25460 | 537 | YPPTYKPKISYPP | Negative |
| Q16665 | 47 | AHQLPLPHNVSSH | Negative |
| P08123 | 669 | RGEIGNPGRDGAR | Negative |
| P02453 | 147 | PGPPGPPGPPGPP | Negative |
| Q02388 | 2470 | GVGLPGPRGERGE | Negative |
| P08123 | 350 | LVGEPGPAGSKGE | Negative |
| P12108 | 274 | KGPPGDPGEKGEL | Negative |
| Q9ZT16 | 77 | TPPSVAPSPADVP | Negative |
| P20908 | 1529 | PSGPIGPPGPPGL | Negative |
| P08123 | 40 | PAGDRGPRGERGP | Negative |
| P20908 | 523 | GTMLMLPFRFGGG | Negative |
| P02453 | 884 | AAGRVGPPGPSGN | Negative |
| P01042 | 148 | CLGCVHPISTQSP | Negative |
| P08123 | 419 | RAGVMGPPGSRGA | Negative |
| P05997 | 569 | PGEPGLPGARGLT | Negative |
| P12108 | 74 | APGPDGPPGKPGL | Negative |
| P12108 | 674 | LGALPTPRHG--- | Negative |
| P02453 | 893 | PSGNAGPPGPPGP | Negative |
| P02453 | 161 | LGGNFAPQLSYGY | Negative |
| P20908 | 1187 | PTGPQGPIGQPGP | Negative |
| P30754 | 344 | IPGEKGPSGEPGA | Negative |
| P0C8W0 | 19 | LLPLANPAENGDG | Negative |
| P20908 | 993 | QGKTGPPGPPGVV | Negative |
| P12111 | 612 | AEFRAAPLQGMLP | Negative |
| P08123 | 1095 | AGPPGPPGPPGPP | Negative |
| Q9U3Z3 | 23 | FLNLVVPTSACRA | Negative |
| Q05707 | 865 | YRIVYKPVSVPGP | Negative |
| P02453 | 482 | PAGLPGPPGERGG | Negative |
| Q02388 | 1838 | PGEDGKPGLNGKN | Negative |
| P08123 | 395 | SAGPPGPPGLRGS | Negative |
| P15502 | 9 | GLTAAAPRPGVLL | Negative |
| P15502 | 194 | GIPGVGPFGGPQP | Negative |
| P24091 | 262 | RAANRLPGFGVIT | Negative |
| P12111 | 432 | HIVLKPPTIVTQV | Negative |
| P05997 | 1285 | IETMRSPDGSKKH | Negative |
| P02453 | 885 | AGRVGPPGPSGNA | Negative |
| P20908 | 1833 | FGFEVGPACFMG- | Negative |
| Q28084 | 204 | PPGTPGPTGEKGN | Negative |
| P30754 | 14 | IQAQVGPIGPRGP | Negative |
| P08123 | 90 | KGVGLGPGPMGLM | Negative |
| P08125 | 470 | APGLPGPAGIVTK | Negative |
| P20908 | 458 | KGQKGEPAIIEPG | Negative |
| Q02388 | 1502 | ERGPPGPAGSRGL | Negative |
| P02453 | 926 | EVGPPGPPGPAGE | Negative |
| P69765 | 23 | KAKDNMPLASFHD | Negative |
| P08125 | 156 | KPGPQGPPGAQGP | Negative |
| P08125 | 157 | PGPQGPPGAQGPR | Negative |
| P0C8V6 | 34 | GLKNLFPKARHEM | Negative |
| Q9M0S4 | 102 | GPTGSTPVDNNNA | Negative |
| Q9C5S0 | 124 | SPPAQVPAPAPTT | Negative |
| P02453 | 383 | PAGAAGPAGNPGA | Negative |
| P15502 | 83 | AGLGAFPAVTFPG | Negative |
| P20908 | 1179 | KGEQGPPGPTGPQ | Negative |
| P15502 | 439 | PGVGISPEAQAAA | Negative |
| P12111 | 1470 | RRLNIGPSKVRVG | Negative |
| P69929 | 169 | RKREPEPNVAVPP | Negative |
| Q02388 | 67 | LEGLVLPFSGAAS | Negative |
| P02453 | 375 | RGEPGPPGPAGAA | Negative |
| P20908 | 1550 | AKGSSGPTGPKGE | Negative |
| O97939 | 324 | PNIRGFPARRQWR | Negative |
| P20908 | 1148 | LPGPAGPVGPPGE | Negative |
| P20908 | 734 | AQGLPGPQGAIGP | Negative |
| Q02388 | 855 | GDREGTPVSIVVT | Negative |
| Q9UKV8 | 26 | GYAFKPPPRPDFG | Negative |
| Q28084 | 103 | PGVPGQPGARGDP | Negative |
| P42849 | 14 | DMEWKRPSDPKFY | Negative |
| Q05707 | 1490 | PKGPDGPRGEIGL | Negative |
| P0C8V6 | 56 | KRDECYPPGTFCG | Negative |
| P15502 | 337 | PGVVGVPGAGVPG | Negative |
| P08427 | 100 | RGLPGFPAYLDEE | Negative |
| Q9Y2N7 | 629 | LQDPSTPLLNLNE | Negative |
| P86289 | 41 | VQGPPGPQGPRGL | Negative |
| O97939 | 1034 | PRPEGIPNEMQGN | Negative |
| Q9UKV8 | 17 | PPAPPPPIQGYAF | Negative |
| P01042 | 405 | ETTVSPPHTSMAP | Negative |
| P01042 | 372 | PTVNCQPLGMISL | Negative |
| Q25460 | 83 | PKMTYPPTYKPKP | Negative |
| P20908 | 746 | PPGEKGPLGKPGL | Negative |
| P05997 | 175 | QPGAPGPPGHPSH | Negative |
| Q02388 | 2756 | ERVVGAPGVPGAP | Negative |
| P20908 | 1539 | PGLPGPPGPKGAK | Negative |
| Q02388 | 1289 | PGPQGPPGSATAK | Negative |
| P08125 | 224 | NGLPGQPGMKGDR | Negative |
| O97939 | 716 | LYGEFYPWNPEEN | Negative |
| P20908 | 1278 | PGGIGNPGAVGEK | Negative |
| P69929 | 174 | EPNVAVPPCGDCY | Negative |
| P08661 | 231 | GKWNDVPCSDSFL | Negative |
| P20908 | 1233 | VGLQGLPGPPGEK | Negative |
| P08123 | 489 | AGARGEPGNIGFP | Negative |
| P12111 | 161 | KDGLALPSAELKS | Negative |
| P20908 | 1493 | LIGLIGPPGEQGE | Negative |
| Q02388 | 1285 | RTGAPGPQGPPGS | Negative |
| P00877 | 176 | LGCTIKPKLGLSA | Negative |
| P20908 | 507 | PGRPGLPGADGLP | Negative |
| P08123 | 467 | PAGKEGPVGLPGI | Negative |
| Q02388 | 2078 | QGRDGPPGLPGTP | Negative |
| P08123 | 128 | EPGQTGPAGARGP | Negative |
| Q9UKV8 | 323 | KLVLRYPHLPCLQ | Negative |
| O97939 | 650 | PYNEEDPIDPTGD | Negative |
| Q02388 | 501 | TPATVVPTGPELP | Negative |
| Q28084 | 109 | PGARGDPGFYGFP | Negative |
| P20908 | 1347 | VGFPGDPGPPGEP | Negative |
| P02820 | 4 | #NAME? | Negative |
| P20908 | 833 | DRGEIGPPGPRGE | Negative |
| P12111 | 3083 | KKSQPPPPQPARS | Negative |
| P20908 | 1565 | HPGPPGPPGPPGE | Negative |
| P30754 | 635 | PAGERGPAGSQGI | Negative |
| P05997 | 1292 | DGSKKHPARTCDD | Negative |
| P23805 | 285 | KGQLASPRSSAEN | Negative |
| Q9C5S0 | 102 | PVASPPPATPPPV | Negative |
| Q8LG54 | 69 | SPPSSAPSPSSDA | Negative |
| Q05707 | 14 | MRYWLLPPFLAIV | Negative |
| P02453 | 129 | RGPAGPPGRDGIP | Negative |
| Q28084 | 129 | NSGFPGPPGPPGQ | Negative |
| P15502 | 685 | GVVGAGPAAAAAA | Negative |
| Q02388 | 209 | ILRTLLPLVSRRV | Negative |
| P12111 | 2933 | TATVRPPVAVKPA | Negative |
| P12111 | 1603 | REFRELPNIEERI | Negative |
| Q02388 | 1387 | PLGDPGPRGPPGL | Negative |
| Q16665 | 595 | SPESASPQSTVTV | Negative |
| Q05707 | 1017 | RPPTFPPTIPPAK | Negative |
| P08123 | 500 | FPGPKGPTGDPGK | Negative |
| Q3Y5Z3 | 121 | ERQVTVPNVPIRF | Negative |
| O97939 | 796 | KTPTSSPHQKENQ | Negative |
| P02453 | 1001 | PPGPMGPPGLAGP | Negative |
| P0C1N1 | 27 | PLDGDQPADRPAE | Negative |
| P12111 | 1045 | GVRSGFPLLKEFV | Negative |
| P02453 | 312 | PGERGRPGAPGPA | Negative |
| Q25460 | 507 | YPPTYKPKLTYKP | Negative |
| P02453 | 923 | RPGEVGPPGPPGP | Negative |
| O97939 | 595 | RVHVYYPDYNPYD | Negative |
| P20908 | 968 | PKGPPGPPGKDGL | Negative |
| P08123 | 300 | VGPPGNPGANGLT | Negative |
| Q02388 | 510 | PELPVSPVTDLQA | Negative |
| P02453 | 899 | PPGPPGPAGKEGS | Negative |
| P12111 | 2882 | PVTTTKPVTTTTK | Negative |
| Q02388 | 1713 | RLVDTGPGAREKG | Negative |
| O97939 | 150 | SPTPTQPEEETQT | Negative |
| P05997 | 179 | PGPPGHPSHPGPD | Negative |
| P01042 | 574 | LIATMMPPISPAP | Negative |
| P20908 | 1371 | KGDDGEPGQTGSP | Negative |
| P23805 | 119 | PAGREGPSGKQGS | Negative |
| Q02388 | 2382 | PGSPGPPGPPGVK | Negative |
| P02453 | 225 | RGPPGPPGKNGDD | Negative |
| P69929 | 161 | RVPSLCPSRKREP | Negative |
| P12108 | 155 | PGPPGKPGPPGHI | Negative |
| P12111 | 1000 | LAAESLPKIGDLH | Negative |
| P12108 | 412 | KGEQGPPGIPGPQ | Negative |
| P02453 | 806 | DRGEPGPPGPAGF | Negative |
| P12111 | 793 | EQIAFNPSLVYLM | Negative |
| P12108 | 202 | PGALGEPGQQGKQ | Negative |
| P01042 | 618 | PKCPGRPWKSVSE | Negative |
| Q0VKG8 | 55 | EERRDRPPSWIPK | Negative |
| Q02388 | 764 | AGVDGPPASVVVR | Negative |
| P69929 | 123 | NTCVRVPSLCPSR | Negative |
| P23805 | 336 | NWADGEPNNSDEG | Negative |
| Q16665 | 437 | YNDVMLPSPNEKL | Negative |
| P08123 | 168 | QGARGFPGTPGLP | Negative |
| P05997 | 493 | PHGIQGPIGPPGE | Negative |
| P12111 | 1908 | DFDEYQPEMLEKF | Negative |
| P02453 | 291 | KGEPGSPGENGAP | Negative |
| Q16665 | 138 | VFDFTHPCDHEEM | Negative |
| P08123 | 288 | RGEVGLPGLSGPV | Negative |
| P02453 | 191 | PRGLPGPPGAPGP | Negative |
| P12111 | 2397 | YGPLECPVFPTEL | Negative |
| O97939 | 540 | KHSAYQPVYTEGI | Negative |
| P12111 | 2604 | GFGSWRPSFRDRR | Negative |
| P05997 | 682 | FQGLPGPPGPPGE | Negative |
| P05997 | 671 | RGEQGPPGPTGFQ | Negative |
| Q02388 | 520 | LQATELPGQRVRV | Negative |
| Q9ZT16 | 68 | PAATPAPATTPPS | Negative |
| Q9C5S0 | 107 | PPATPPPVATPPP | Negative |
| Q05707 | 1406 | MVRSRGPGGNSAP | Negative |
| Q02388 | 2759 | VGAPGVPGAPGER | Negative |
| P12108 | 325 | AGQPGRPGPPGHR | Negative |
| P20908 | 770 | HPGKEGPPGEKGG | Negative |
| Q9UKV8 | 800 | TRSVSIPAPAYYA | Negative |
| P20908 | 572 | LTGRPGPVGPPGS | Negative |
| P02453 | 1029 | PGRDGSPGAKGDR | Negative |
| Q9M0S4 | 81 | PSSASPPAPPTSL | Negative |
| Q25460 | 381 | PTYKAKPTYKAKP | Negative |
| P08123 | 315 | KGAAGLPGVAGAP | Negative |
| P05997 | 452 | AGPPGSPGPQGST | Negative |
| Q02388 | 1605 | LTGRAGPPGDSGP | Negative |
| P69929 | 106 | EPNVAVPPCGDCY | Negative |
| Q02388 | 1756 | FRGPPGPQGDPGV | Negative |
| Q02388 | 1825 | LPGPSGPPGLPGK | Negative |
| P02453 | 908 | KEGSKGPRGETGP | Negative |
| P02453 | 975 | RGFPGLPGPSGEP | Negative |
| P08123 | 147 | AGEDGHPGKPGRP | Negative |
| P02745 | 75 | DQGEPGPSGNPGK | Negative |
| P30754 | 44 | EPGDSGPMGPIGK | Negative |
| P20908 | 992 | FQGKTGPPGPPGV | Negative |
| P02453 | 1055 | APGAPGPVGPAGK | Negative |
| Q05707 | 1752 | VPGPQGPSGQPGY | Negative |
| Q02388 | 1271 | RGQVGPPGDPGLP | Negative |
| P02453 | 927 | VGPPGPPGPAGEK | Negative |
| P05997 | 1345 | ANPSSVPRKTWWA | Negative |
| P20908 | 312 | VPEELTPTPTEAA | Negative |
| P05997 | 1223 | PGPPGPPGPPGHL | Negative |
| P30754 | 581 | SRGDIGPRGKAGE | Negative |
| P20908 | 296 | PTPSKKPVEAAKE | Negative |
| P05997 | 728 | PGITGLPGEKGMA | Negative |
| P08427 | 165 | GGNIAVPRTPEEN | Negative |
| Q28084 | 56 | PGMPGPPGPPGSP | Negative |
| O97939 | 1028 | GTPDKEPRPEGIP | Negative |
| P02453 | 582 | AGVMGFPGPKGAA | Negative |
| P02453 | 941 | APGADGPAGAPGT | Negative |
| Q28084 | 178 | QGEPGPPGPPGDP | Negative |
| Q8LG54 | 83 | TASPPAPEGPGVS | Negative |
| P29602 | 16 | NTGWSVPSSPNFY | Negative |
| Q02388 | 1894 | PPGLPGPVGPPGQ | Negative |
| O97939 | 47 | MQMPRMPGFSSKS | Negative |
| P02453 | 317 | RPGAPGPAGARGN | Negative |
| Q9XF04 | 85 | LHHHVNPPRQPRN | Negative |
| P05997 | 563 | QGDPGRPGEPGLP | Negative |
| Q05707 | 1714 | TQGPRGPPGPAGP | Negative |
| Q02388 | 1372 | RKGDPGPSGPPGP | Negative |
| P05997 | 142 | PPGSQGPRGERGP | Negative |
| P15502 | 405 | VGAGGFPGFGVGV | Negative |
| P58808 | 18 | LLTASAPGVVVLP | Negative |
| Q28084 | 202 | DGPPGTPGPTGEK | Negative |
| Q05707 | 595 | PLKGLTPLTEYTI | Negative |
| P12108 | 157 | PPGKPGPPGHIQG | Negative |
| P58925 | 16 | ICLLLFPITALPM | Negative |
| Q02388 | 2214 | EPGETGPPGRGLT | Negative |
| P02453 | 215 | EPGASGPMGPRGP | Negative |
| Q9M0S4 | 75 | PAGSPLPSSASPP | Negative |
| Q9Y2N7 | 386 | SLDTPGPRILAFL | Negative |
| Q28084 | 336 | IPMDMAPITGRAL | Negative |
| P12111 | 1633 | TPPPSRPEKKKAD | Negative |
| P02453 | 948 | AGAPGTPGPQGIA | Negative |
| P02745 | 243 | SGFLIFPSA---- | Negative |
| Q02388 | 1322 | PGTPGAPGLKGSP | Negative |
| Q9UKV8 | 489 | MPIQGQPCFCKYA | Negative |
| Q9UKV8 | 580 | VNNILLPQGRPPV | Negative |
| Q02388 | 1805 | AGKAGDPGRDGLP | Negative |
| Q16665 | 590 | ESSSASPESASPQ | Negative |
| O97939 | 119 | QQSKTDPAPESQK | Negative |
| P02747 | 89 | HPGKNGPMGPPGM | Negative |
| P20908 | 1256 | PPGPPGPRGPSGA | Negative |
| P08125 | 481 | TKGLRGPMGPLGP | Negative |
| P20908 | 1004 | VVGPQGPTGETGP | Negative |
| P12111 | 2076 | PGERGPPGVNGTQ | Negative |
| Q02388 | 2568 | KGSKGEPGDKGSA | Negative |
| P02453 | 315 | RGRPGAPGPAGAR | Negative |
| Q60994 | 29 | TTEELAPALVPPP | Negative |
| Q15485 | 59 | PGLPGAPGPKGEA | Negative |
| Q9ZT16 | 79 | PSVAPSPADVPTA | Negative |
| P08123 | 909 | PGARGPPGAVGSP | Negative |
| P24091 | 115 | NAARSFPGFGTSG | Negative |
| P05997 | 920 | AGRVGPPGPAGAP | Negative |
| P12111 | 1541 | RIEDGVPQHLVLV | Negative |
| P08125 | 151 | LSVLGKPGPQGPP | Negative |
| Q05707 | 1347 | TVTFEGPEIRKIF | Negative |
| P08123 | 563 | LPGPSGPAGEVGK | Negative |
| P04925 | 67 | GGGWGQPHGGSWG | Negative |
| Q02388 | 2234 | LPGPPGPSGLVGP | Negative |
| P02453 | 851 | PAGPPGPIGNVGA | Negative |
| P58787 | 40 | VPRDDNPSGTDGK | Negative |
| Q02388 | 1242 | ASFTTQPRPEPCP | Negative |
| P02746 | 32 | QLSCTGPPAIPGI | Negative |
| P20908 | 21 | PGAPLLPPLLLLL | Negative |
| P05997 | 1171 | SAGIPGPFGPRGP | Negative |
| Q25460 | 807 | PKLTYPPTYKPKP | Negative |
| Q9UKV8 | 288 | CNVTRRPASHQTF | Negative |
| P15502 | 62 | LGPGGKPLKPVPG | Negative |
| P08123 | 903 | LGIAGPPGARGPP | Negative |
| Q16665 | 216 | CGYKKPPMTCLVL | Negative |
| O97939 | 169 | FGNGLFPYQQPLW | Negative |
| Q02388 | 1629 | PPGPVGPRGRDGE | Negative |
| P02453 | 620 | EAGAQGPPGPAGP | Negative |
| P19999 | 155 | RGTVAIPRNAEEN | Negative |
| P12108 | 119 | PSLPGPPGLPGQV | Negative |
| Q02388 | 1426 | PGEPGLPGLPGSP | Negative |
| Q02388 | 1576 | VQGERGPPGLVLP | Negative |
| P02453 | 1088 | ARGPAGPQGPRGD | Negative |
| P20908 | 914 | PRGQRGPTGPRGE | Negative |
| Q02388 | 2875 | PWDSDDPCSLPLD | Negative |
| P30754 | 23 | PRGPPGPPGSPGQ | Negative |
| Q9UKV8 | 16 | APPAPPPPIQGYA | Negative |
| Q02388 | 944 | GPAGEGPSAEVTA | Negative |
| P12111 | 2524 | TPTRASPQLREAV | Negative |
| P69928 | 43 | PSVHGNPLSGTIW | Negative |
| Q02388 | 2379 | VGVPGSPGPPGPP | Negative |
| P23805 | 185 | PSGAIGPQGPSGA | Negative |
| O97939 | 387 | TARSNPPNYAGNS | Negative |
| Q25460 | 108 | KPKITYPPTYKAK | Negative |
| P00877 | 453 | SACKWSPELAAAC | Negative |
| P15502 | 142 | VKPGKVPGVGLPG | Negative |
| P05997 | 638 | AGNAGVPGQRGAP | Negative |
| P12111 | 2033 | KACCGVPCKCSGQ | Negative |
| Q9UKV8 | 50 | FFEMDIPKIDIYH | Negative |
| P12108 | 271 | EGPKGPPGDPGEK | Negative |
| P15502 | 755 | GGAGQFPLGGVAA | Negative |
| P20908 | 1566 | PGPPGPPGPPGEV | Negative |
| P05997 | 284 | VGFAGSPGARGFP | Negative |
| P02453 | 798 | TGARGAPGDRGEP | Negative |
| P05997 | 1495 | FGVEIGPVCFV-- | Negative |
| Q05707 | 584 | NEVEVDPITTFPL | Negative |
| P08125 | 506 | PGPPGPPGPPGQS | Negative |
| P69929 | 33 | IKREPEPNVAVPP | Negative |
| P12108 | 177 | LCPTNCPPGPKGP | Negative |
| Q9ZT16 | 108 | GPSDASPAPSAAF | Negative |
| P08123 | 560 | FQGLPGPSGPAGE | Negative |
| Q02388 | 2240 | PSGLVGPQGSPGL | Negative |
| P05997 | 1258 | TEDQAAPDDKNKT | Negative |
| O97939 | 452 | TRDPSGPWRNSQD | Negative |
| O97939 | 414 | MVTNVAPPGPKHG | Negative |
| P02453 | 393 | PGADGQPGAKGAN | Negative |
| Q02388 | 1085 | LVLALGPLGPQAV | Negative |
| Q9UKV8 | 622 | GSMDAHPNRYCAT | Negative |
| Q9UKV8 | 602 | ADVTHPPAGDGKK | Negative |
| P12111 | 1733 | RVNHFVPEAGSRL | Negative |
| Q02388 | 1787 | SGLDGKPGAAGPS | Negative |
| P20908 | 1143 | QGPVGLPGPAGPV | Negative |
| P20908 | 782 | GQGPPGPQGPIGY | Negative |
| P20908 | 689 | LLGPKGPPGPPGP | Negative |
| Q7XAD0 | 108 | DTVAAPPVGGRHD | Negative |
| P12108 | 655 | RPGSPGPAGLPGF | Negative |
| P00877 | 89 | RCYDIEPVPGEDN | Negative |
| P12108 | 113 | PPGLPGPSLPGPP | Negative |
| Q02388 | 1873 | REGRDGPKGERGA | Negative |
| P69929 | 229 | RVPSLCPSRKR-- | Negative |
| P02453 | 950 | APGTPGPQGIAGQ | Negative |
| P01042 | 252 | QNCDIYPGKDFVQ | Negative |
| P01042 | 84 | IKEGDCPVQSGKT | Negative |
| P01042 | 366 | WEKKIYPTVNCQP | Negative |
| Q02388 | 138 | LPQLARPGVPKVC | Negative |
| P05997 | 148 | PRGERGPKGRPGP | Negative |
| P02745 | 143 | ITNQEEPYQNHSG | Negative |
| P08123 | 1001 | PRGPSGPQGIRGD | Negative |
| P24091 | 180 | GQWPCAPGRKYFG | Negative |
| Q16665 | 429 | QQLEEVPLYNDVM | Negative |
| P05997 | 370 | KPGPMGPLGIPGS | Negative |
| Q9UKV8 | 170 | VVMRHLPSMRYTP | Negative |
| P20908 | 1538 | PPGLPGPPGPKGA | Negative |
| O97939 | 261 | QPNAPNPRGNDTS | Negative |
| Q93WP8 | 91 | NQLFSFPTSADNY | Negative |
| Q28084 | 302 | QRFTTMPFLFCNI | Negative |
| P12111 | 1622 | SAATPAPPGVDTP | Negative |
| P23805 | 179 | RAGVTGPSGAIGP | Negative |
| P30754 | 401 | KQGEQGPQGPQGL | Negative |
| Q9Y2N7 | 42 | QLAHTLPFARGVS | Negative |
| P20908 | 650 | HRGDPGPSGPPGP | Negative |
