# Supplementary material for: Prediction and Analysis of Protein Hydroxyproline and Hydroxylysine
Source: PLoS One. 2010 Dec 31;5(12):e15917. doi: 10.1371/journal.pone.0015917 (PMC3013141; doi:10.1371/journal.pone.0015917)
Supplement: Table S2 — 324 peptides extracted from hydroxylysine dataset. (DOC) [file pone.0015917.s002.doc]

**Table S2. 324 peptides extracted from hydroxylysine dataset**

Peptides with 13 residues that consisted of a proline residue, 6 residues upstream and 6 residues downstream of the proline residue were extracted from the protein sequences in hydroxylysine dataset. If the peptides exceeded the boundary of protein sequence, we inserted the non-existent residues coded by ‘-’ to make up of the peptides with 13 residues. Totally, there were 1025 peptides consisting of 108 hydroxylated lysine residues and 917 non-hydroxylated lysine residues with experimental verification in the dataset. Then the 108 peptides with hydroxylated lysine residues were assigned as positive samples, while 216 peptides that were randomly selected from the 917 peptides containing non-hydroxylated lysine residues were assigned as negative samples. All the 324 samples are listed as follows.

| Uniprot-AC | Position | Peptide | Class |
| --- | --- | --- | --- |
| P23805 | 63 | ECPHGEKGDPGSP | Positive |
| P23805 | 87 | VGPIGPKGDNGFV | Positive |
| P23805 | 99 | VGEPGPKGDTGPR | Positive |
| P23805 | 135 | PGTPGPKGETGPK | Positive |
| P23805 | 141 | KGETGPKGGVGAP | Positive |
| P23805 | 159 | PGPSGLKGEKGAP | Positive |
| P23805 | 162 | SGLKGEKGAPGET | Positive |
| P23805 | 198 | RGPPGLKGDRGDP | Positive |
| P23805 | 210 | PGETGAKGESGLA | Positive |
| P02461 | 263 | PGFPGMKGHRGFD | Positive |
| P02461 | 284 | TGAPGLKGENGLP | Positive |
| P02461 | 860 | PGPQGVKGERGSP | Positive |
| P02461 | 977 | PGPQGVKGESGKP | Positive |
| P02461 | 1094 | QGPRGDKGETGER | Positive |
| P02461 | 1106 | RGAAGIKGHRGFP | Positive |
| P19999 | 44 | DGRDGPKGEKGEP | Positive |
| P19999 | 47 | DGPKGEKGEPGQG | Positive |
| Q00433 | 56 | AGAAMDKSAKAPQ | Positive |
| Q60994 | 68 | DGTPGEKGEKGDA | Positive |
| Q60994 | 71 | PGEKGEKGDAGLL | Positive |
| Q60994 | 80 | AGLLGPKGETGDV | Positive |
| Q60994 | 104 | PGTPGRKGEPGEA | Positive |
| P02452 | 265 | AGLPGMKGHRGFS | Positive |
| Q3Y5Z3 | 28 | EDPPLPKGACAGW | Positive |
| Q3Y5Z3 | 60 | DGTPGEKGEKGDP | Positive |
| Q3Y5Z3 | 63 | PGEKGEKGDPGLV | Positive |
| Q3Y5Z3 | 72 | PGLVGPKGDTGET | Positive |
| Q3Y5Z3 | 96 | PGTPGRKGEPGES | Positive |
| Q05707 | 1476 | PGLRGPKGQQGEP | Positive |
| Q05707 | 1485 | QGEPGPKGPDGPR | Positive |
| Q05707 | 1523 | PGMPGEKGEKGDT | Positive |
| Q05707 | 1526 | PGEKGEKGDTGLP | Positive |
| Q05707 | 1601 | PGVPGAKGERGER | Positive |
| Q05707 | 1698 | RGLTGIKGEKGNP | Positive |
| Q05707 | 1701 | TGIKGEKGNPGVG | Positive |
| P02746 | 57 | PGTPGIKGEKGLP | Positive |
| P02746 | 60 | PGIKGEKGLPGLA | Positive |
| P02746 | 75 | HGEFGEKGDPGIP | Positive |
| P02746 | 90 | PGKVGPKGPMGPK | Positive |
| P02746 | 96 | KGPMGPKGGPGAP | Positive |
| P02746 | 108 | PGAPGPKGESGDY | Positive |
| P35248 | 86 | RGPVGPKGENGSA | Positive |
| P35248 | 98 | AGEPGPKGERGLV | Positive |
| P30754 | 183 | PGTPGSKGDRGQS | Positive |
| P30754 | 342 | MGIPGEKGPSGEP | Positive |
| P30754 | 351 | SGEPGAKGPTGDT | Positive |
| P30754 | 546 | PGVAGAKGQGGPP | Positive |
| P30754 | 567 | PGERGPKGVGGSK | Positive |
| P30754 | 933 | KGEAGGKGAKGDK | Positive |
| P30754 | 939 | KGAKGDKGWTGLP | Positive |
| O18495 | 27 | EAGWLRKAAKSVG | Positive |
| O18495 | 30 | WLRKAAKSVGKFY | Positive |
| O18495 | 34 | AAKSVGKFYYKHK | Positive |
| O18495 | 38 | VGKFYYKHKYYIK | Positive |
| O18495 | 40 | KFYYKHKYYIKAA | Positive |
| O18495 | 44 | KHKYYIKAAWQIG | Positive |
| P02745 | 33 | RAPDGKKGEAGRP | Positive |
| P02745 | 48 | RGRPGLKGEQGEP | Positive |
| P02745 | 67 | TGIQGLKGDQGEP | Positive |
| P02745 | 100 | RGIPGIKGTKGSP | Positive |
| P02745 | 103 | PGIKGTKGSPGNI | Positive |
| P02454 | 254 | AGLPGMKGHRGFS | Positive |
| P04258 | 95 | PGPPGMKGPAGMP | Positive |
| P04258 | 107 | PGFPGMKGHRGFD | Positive |
| P04258 | 119 | DGRNGEKGEPGAP | Positive |
| P04258 | 938 | QGPRGDKGETGER | Positive |
| P04258 | 950 | RGAMGIKGHRGFP | Positive |
| P02453 | 264 | AGLPGMKGHRGFS | Positive |
| P01170 | 120 | CKNFYWKGFTSC- | Positive |
| Q9Y383 | 266 | RSRSHSKNPKRSR | Positive |
| Q9Y383 | 269 | SHSKNPKRSRSRE | Positive |
| P20908 | 627 | PGQTGPKGDRGFD | Positive |
| P20908 | 642 | AGLPGEKGHRGDP | Positive |
| P20908 | 708 | DGQPGPKGNVGPQ | Positive |
| P20908 | 744 | IGPPGEKGPLGKP | Positive |
| P20908 | 774 | EGPPGEKGGQGPP | Positive |
| P20908 | 795 | PGPRGVKGADGIR | Positive |
| P20908 | 804 | DGIRGLKGTKGEK | Positive |
| P20908 | 807 | RGLKGTKGEKGED | Positive |
| P20908 | 810 | KGTKGEKGEDGFP | Positive |
| P20908 | 819 | DGFPGFKGDMGIK | Positive |
| P20908 | 846 | DGPEGPKGRGGPN | Positive |
| P20908 | 864 | LGPPGEKGKLGVP | Positive |
| P20908 | 882 | PGRQGPKGSIGFP | Positive |
| P20908 | 897 | PGANGEKGGRGTP | Positive |
| Q02388 | 2625 | MGPRGLKGERGVK | Positive |
| Q02388 | 2631 | KGERGVKGACGLD | Positive |
| P26368 | 15 | RQLNENKQERDKE | Positive |
| P26368 | 276 | LNDDQVKELLTSF | Positive |
| Q15848 | 65 | DGTPGEKGEKGDP | Positive |
| Q15848 | 68 | PGEKGEKGDPGLI | Positive |
| Q15848 | 77 | PGLIGPKGDIGET | Positive |
| Q15848 | 101 | PGIQGRKGEPGEG | Positive |
| P04142 | 47 | IRDGIVKAGPAIE | Positive |
| P30943 | 41 | VNMAMDKSAKAPV | Positive |
| P12111 | 2103 | RGFPGEKGEVGEI | Positive |
| P12111 | 2209 | RGPPGAKGNKGGP | Positive |
| P12111 | 2212 | PGAKGNKGGPGQP | Positive |
| P12111 | 2322 | PGYPGPKGNPGEP | Positive |
| P12111 | 2337 | NGTTGPKGIRGRR | Positive |
| Q9SE35 | 174 | GSYSGSKGSKRRI | Positive |
| Q9SE35 | 193 | GSYSGSKGSKRRN | Positive |
| Q9SE35 | 212 | GSYSGSKGSKRRI | Positive |
| Q9SE35 | 231 | GSYSGSKGSKRRN | Positive |
| Q9SE35 | 250 | GSYSGSKGSKRRI | Positive |
| P02747 | 57 | DGLPGPKGEPGIP | Positive |
| P02747 | 75 | RGPKGQKGEPGLP | Positive |
| P12108 | 181 | NCPPGPKGPQGLQ | Positive |
| P02461 | 350 | PGSPGAKGEVGPA | Negative |
| Q02388 | 2113 | QGPPGLKGAKGEP | Negative |
| P02461 | 46 | ADRDVWKPEPCQI | Negative |
| Q05707 | 1311 | LWEILNKNSDPLV | Negative |
| P02454 | 748 | ADGSPGKDGVRGL | Negative |
| P19999 | 106 | AEINTLKSKLELT | Negative |
| P35248 | 322 | DVGTEGKFTYPTG | Negative |
| P35248 | 62 | EGPRGEKGDPGLP | Negative |
| P02453 | 834 | PGDAGAKGDAGPP | Negative |
| P20908 | 164 | INLSDGKWHRIAL | Negative |
| P02745 | 110 | GSPGNIKDQPRPA | Negative |
| P12108 | 295 | QGDIGPKGDMGLP | Negative |
| Q02388 | 2780 | AGPRGEKGEAALT | Negative |
| P02452 | 586 | MGFPGPKGAAGEP | Negative |
| Q05707 | 51 | SIQISWKAPRGKF | Negative |
| Q02388 | 2457 | PGASGLKGDKGDP | Negative |
| Q02388 | 2116 | PGLKGAKGEPGSN | Negative |
| P12111 | 1772 | LTQRGVKVFAVGV | Negative |
| P12111 | 1856 | NVFVAQKGFESKV | Negative |
| P35248 | 118 | ISGPAGKEGPSGK | Negative |
| P12108 | 211 | QGKQGPKGDVGVS | Negative |
| Q05707 | 382 | APGNVEKYRVVYY | Negative |
| Q05707 | 1091 | FKLNAYKTKETLL | Negative |
| P02747 | 14 | LPHLGLKLLLLLL | Negative |
| P04258 | 767 | PGISGPKGDSGPP | Negative |
| P02746 | 157 | YEPRSGKFTCKVP | Negative |
| P20908 | 989 | ETGFQGKTGPPGP | Negative |
| P20908 | 1826 | FGEASQKFGFEVG | Negative |
| Q05707 | 754 | TSSLRVKWDISDS | Negative |
| P02452 | 1321 | SKNPKDKRHVWFG | Negative |
| P20908 | 971 | PPGPPGKDGLPGH | Negative |
| Q05707 | 1326 | ILDNGGKTLTYFN | Negative |
| P04142 | 59 | EVLGSAKAIGK-- | Negative |
| Q02388 | 1367 | PGLPGRKGDPGPS | Negative |
| P12111 | 551 | AAEGIPKLLVLIT | Negative |
| P12111 | 2472 | KSVLLDKIKNLQV | Negative |
| P02452 | 781 | AGAPGDKGESGPS | Negative |
| P30754 | 146 | ERGRDGKSGLPGL | Negative |
| P35248 | 236 | MEALNGKLQRLEA | Negative |
| Q02388 | 1786 | RSGLDGKPGAAGP | Negative |
| P02746 | 119 | DYKATQKIAFSAT | Negative |
| P20908 | 1473 | PGLPGLKGDSGPK | Negative |
| P02746 | 161 | SGKFTCKVPGLYY | Negative |
| Q60994 | 183 | SLFKKDKAVLFTY | Negative |
| P02452 | 1152 | SAGAPGKDGLNGL | Negative |
| P02454 | 1225 | EVDTTLKSLSQQI | Negative |
| Q02388 | 2341 | PGPRGEKGEAGRA | Negative |
| Q02388 | 2571 | KGEPGDKGSAGLP | Negative |
| P02461 | 1388 | ASGNVKKALKLMG | Negative |
| P02454 | 160 | SYGYDEKSAGVSV | Negative |
| P20908 | 1284 | PGAVGEKGEPGEA | Negative |
| P02461 | 1256 | SPDGSRKNPARNC | Negative |
| Q02388 | 1402 | TAMKGDKGDRGER | Negative |
| O18495 | 51 | AAWQIGKHALGDM | Negative |
| P02745 | 32 | CRAPDGKKGEAGR | Negative |
| P12111 | 964 | GPASNLKQSGVVP | Negative |
| P04258 | 428 | MGFPGPKGNDGAP | Negative |
| Q05707 | 270 | ILITDGKSQDDII | Negative |
| P02452 | 114 | TGVEGPKGDTGPR | Negative |
| P20908 | 1410 | QGEKGAKGEAGLE | Negative |
| P12111 | 2854 | LVKFGHKQVNVPN | Negative |
| Q05707 | 61 | GKFGGYKLLVTPT | Negative |
| P02453 | 611 | AVGPAGKDGEAGA | Negative |
| P12108 | 87 | DGLTGAKGSRGPW | Negative |
| P12111 | 3 | #NAME? | Negative |
| P26368 | 300 | SATGLSKGYAFCE | Negative |
| Q02388 | 1639 | DGEVGEKGDEGPP | Negative |
| P26368 | 84 | RSPRHEKKKKVRK | Negative |
| Q9Y383 | 135 | LNEEIGKLLAKVE | Negative |
| Q02388 | 1399 | LPGTAMKGDKGDR | Negative |
| P12111 | 2846 | QGDQPTKNLVKFG | Negative |
| P12111 | 1946 | SSPDSVKVVIHFT | Negative |
| P12111 | 585 | AFAIGNKGADQAE | Negative |
| P12108 | 406 | AGQSGPKGEQGPP | Negative |
| P02461 | 757 | ADGVPGKDGPRGP | Negative |
| P02461 | 923 | PGVSGPKGDAGQP | Negative |
| P02453 | 1032 | DGSPGAKGDRGET | Negative |
| P35248 | 362 | NGQWNDKACGEQR | Negative |
| Q60994 | 175 | VYMKDVKVSLFKK | Negative |
| P30943 | 101 | LATSLAKMTK--- | Negative |
| Q9Y383 | 367 | DRDRKDKKRSYES | Negative |
| P12111 | 505 | FNTHPTKREVITA | Negative |
| P02461 | 901 | PSGSPGKDGPPGP | Negative |
| P12111 | 783 | GASQANKAELEQI | Negative |
| Q05707 | 1004 | SVSIMEKTQSLPT | Negative |
| P02747 | 117 | EGRYKQKFQSVFT | Negative |
| P19999 | 63 | LQGPPGKLGPPGS | Negative |
| P19999 | 114 | KLELTNKLHAFSM | Negative |
| Q02388 | 1801 | PNGAAGKAGDPGR | Negative |
| P12111 | 2130 | PGSSGEKGNPGRR | Negative |
| P20908 | 1335 | PGDDGPKGSPGPV | Negative |
| P12111 | 699 | LNTYQTKSDILGH | Negative |
| P12111 | 2640 | QFNEMKKYIAYLV | Negative |
| Q05707 | 796 | QNNLLLKPLLPDT | Negative |
| P26368 | 38 | SRSRDRKRRSRSR | Negative |
| P20908 | 1034 | LPGLAGKEGTKGD | Negative |
| P26368 | 462 | NRVVVTKYCDPDS | Negative |
| P02452 | 1288 | CNLDAIKVFCNME | Negative |
| P12111 | 2948 | AKPVAAKPAAVRP | Negative |
| P02453 | 1061 | PVGPAGKSGDRGE | Negative |
| Q02388 | 2603 | DPGSPGKDGVPGI | Negative |
| P26368 | 329 | MQLGDKKLLVQRA | Negative |
| P02453 | 983 | PSGEPGKQGPSGA | Negative |
| P04258 | 601 | VDGAPGKDGPRGP | Negative |
| Q02388 | 1672 | RGPVGEKGDQGDP | Negative |
| Q02388 | 2388 | PGPPGVKGDLGLP | Negative |
| P04258 | 347 | NGLPGEKGPPGDR | Negative |
| P02453 | 1423 | HTGAWGKTVIEYK | Negative |
| Q9SE35 | 204 | RNLSSKKSGSYSG | Negative |
| P04258 | 305 | PGIAGPKGEDGKD | Negative |
| Q60994 | 172 | HITVYMKDVKVSL | Negative |
| P20908 | 866 | PPGEKGKLGVPGL | Negative |
| P02453 | 906 | AGKEGSKGPRGET | Negative |
| P26368 | 444 | SVFDCQKAMQGLT | Negative |
| Q02388 | 2128 | NGDQGPKGDRGVP | Negative |
| P23805 | 222 | AEVNALKQRVTIL | Negative |
| Q05707 | 1231 | IDLAGFKMMEMFG | Negative |
| P02453 | 1318 | YISKNPKEKRHVW | Negative |
| P02453 | 447 | KGDTGAKGEPGPT | Negative |
| P02461 | 161 | YDSYDVKSGVAVG | Negative |
| P30754 | 113 | SKGEQGKSGNQGP | Negative |
| P12111 | 1048 | SGFPLLKEFVQRV | Negative |
| P02452 | 1385 | QQTGNLKKALLLQ | Negative |
| Q9Y383 | 106 | RTEVAKKRLAETQ | Negative |
| Q00433 | 93 | DDEMMVKVASTKV | Negative |
| P20908 | 146 | YEDHTGKPGPEDY | Negative |
| Q05707 | 805 | LPDTEYKVTVTPI | Negative |
| P20908 | 452 | GGPRGEKGQKGEP | Negative |
| P12111 | 909 | VKRMKIKTGKALN | Negative |
| P12111 | 2639 | FQFNEMKKYIAYL | Negative |
| P20908 | 294 | KEPTPSKKPVEAA | Negative |
| Q9SE35 | 223 | RILSSKKSGSYSG | Negative |
| Q05707 | 655 | ADEGLHKLMWIPV | Negative |
| Q02388 | 459 | GLEPPQKVVLPSD | Negative |
| P02454 | 266 | SGLDGAKGDTGPA | Negative |
| P19999 | 108 | INTLKSKLELTNK | Negative |
| P12111 | 3156 | NKFGSQKECEKVC | Negative |
| Q02388 | 2038 | LAGEPGKPGIPGL | Negative |
| P12111 | 2386 | IQSIKDKCPCCYG | Negative |
| P02452 | 505 | DGVAGPKGPAGER | Negative |
| Q02388 | 1733 | EGPRGPKGDPGLP | Negative |
| Q02388 | 1718 | GPGAREKGEPGDR | Negative |
| P20908 | 69 | ATRRSSKGPDVAY | Negative |
| P02452 | 286 | AGPAGPKGEPGSP | Negative |
| P35248 | 249 | AFSRYKKAALFPD | Negative |
| P12111 | 470 | IRDFIAKVIQRLE | Negative |
| P02452 | 657 | PPGEAGKPGEQGV | Negative |
| P26368 | 340 | RASVGAKNATLVS | Negative |
| P02746 | 2 | #NAME? | Negative |
| P26368 | 85 | SPRHEKKKKVRKY | Negative |
| P12111 | 1347 | VLISSGKSDDEVD | Negative |
| P02454 | 1252 | RTCRDLKMCHSDW | Negative |
| P26368 | 413 | SKYGLVKSIEIPR | Negative |
| Q9SE35 | 155 | SGYSTKKSGSRRI | Negative |
| P20908 | 1479 | KGDSGPKGEKGHP | Negative |
| P20908 | 1119 | PGPAGEKGAPGEK | Negative |
| P20908 | 825 | KGDMGIKGDRGEI | Negative |
| Q05707 | 250 | IFENSFKPEAGSR | Negative |
| P02454 | 67 | CDGVLCKEDLDCP | Negative |
| P02452 | 1062 | PVGPAGKSGDRGE | Negative |
| Q05707 | 7 | MKIFQRKMRYWLL | Negative |
| P35248 | 133 | NIGPQGKPGPKGE | Negative |
| P12108 | 595 | IGNIGPKGKRGEK | Negative |
| P20908 | 1433 | PQGAPGKPGPDGL | Negative |
| P12108 | 154 | PPGPPGKPGPPGH | Negative |
| P20908 | 288 | DPEDLGKEPTPSK | Negative |
| P12111 | 310 | QVLGAVKALGFAG | Negative |
| P04258 | 994 | PSGPPGKDGASGH | Negative |
| P12111 | 2466 | FADSKRKSVLLDK | Negative |
| P02452 | 170 | SYGYDEKSTGGIS | Negative |
| P20908 | 329 | TSEGAGKEEDVGI | Negative |
| P02747 | 157 | YDTSTGKFTCKVP | Negative |
| Q3Y5Z3 | 164 | HITVYLKDVKVSL | Negative |
| Q02388 | 727 | SAHGPEKSQLVSG | Negative |
| Q02388 | 1295 | PGSATAKGERGFP | Negative |
| P12111 | 2697 | DYGSKEKLVDFLS | Negative |
| P12111 | 514 | VITAVRKMKPLDG | Negative |
| P02461 | 932 | AGQPGEKGSPGAQ | Negative |
| Q9SE35 | 242 | RNLSSKKSGSYSG | Negative |
| P12108 | 463 | SGEPGPKGQQGIQ | Negative |
| P12111 | 835 | LAQPESKRDILFL | Negative |
| P23805 | 279 | QLCREAKGQLASP | Negative |
| Q02388 | 2359 | PGEDGQKGAPGPK | Negative |
| P02453 | 1429 | KTVIEYKTTKTSR | Negative |
| P12111 | 1635 | PPSRPEKKKADIV | Negative |
| P26368 | 87 | RHEKKKKVRKYWD | Negative |
| Q05707 | 1558 | QRGLPGKDGSSGP | Negative |
| P02454 | 77 | DCPNPQKREGECC | Negative |
| P02452 | 751 | RGDAGPKGADGSP | Negative |
| P12111 | 2834 | LSPDIRKQCDWFQ | Negative |
| Q9Y383 | 122 | SAEVAAKAERVHE | Negative |
| P02453 | 519 | PGPAGPKGSPGEA | Negative |
| Q02388 | 2093 | PGPPGPKVSVDEP | Negative |
| P02461 | 1323 | TDSSAEKKHVWFG | Negative |
| P12111 | 1637 | SRPEKKKADIVFL | Negative |
| P30943 | 61 | GCSRAPKEYTGAK | Negative |
| P19999 | 162 | RNAEENKAIQEVA | Negative |
| P02454 | 770 | AGAPGDKGETGPS | Negative |
| P04258 | 473 | TGPSGDKGDTGPP | Negative |
| P02454 | 1051 | PVGPAGKNGDRGE | Negative |
| Q02388 | 2368 | PGPKGFKGDPGVG | Negative |
| P02454 | 851 | VGAPGPKGSRGAA | Negative |
| P20908 | 605 | PPGPAGKPGRRGR | Negative |
| P12111 | 33 | QQQADVKNGAAAD | Negative |
| Q3Y5Z3 | 187 | HDQFQDKNVDQAS | Negative |
| P12108 | 385 | AGVPGLKGDRGER | Negative |
| P12111 | 2384 | ALIQSIKDKCPCC | Negative |
| P02454 | 583 | TAGEPGKAGERGV | Negative |
| P02452 | 907 | AGKEGGKGPRGET | Negative |
| P12111 | 3113 | TETDICKLPKDEG | Negative |
| P02461 | 1273 | FCHPELKSGEYWV | Negative |
| Q05707 | 1050 | GDENFNKIISFLY | Negative |
| P02452 | 277 | SGLDGAKGDAGPA | Negative |
| Q05707 | 71 | TPTSGGKTNQLNL | Negative |
| P12111 | 904 | EILNLVKRMKIKT | Negative |
| P12111 | 3077 | HGSFSTKKSQPPP | Negative |
