# Supplementary material for: Prediction and Analysis of Protein Hydroxyproline and Hydroxylysine
Source: PLoS One. 2010 Dec 31;5(12):e15917. doi: 10.1371/journal.pone.0015917 (PMC3013141; doi:10.1371/journal.pone.0015917)
Supplement: Table S4 — The MaxRel feature list and the mRMR feature list for hydroxylysine dataset. (DOC) [file pone.0015917.s004.doc]

**Table S4. The MaxRel feature list and the mRMR feature list for hydroxylysine dataset**

Each of the following 2 tables consists of 500 features selected by the mRMR feature selection method from the 6,345-D feature space based on the peptides extracted from the hydroxylysine dataset. The features in MaxRel feature list were sorted according to the Max-Relevance criterion, while the features in mRMR feature list were ranked according to both the Max-Relevance criterion and the Min-Redundancy criterion. See the text of the paper for further explanation.

**MaxRel Feature List**

| Order | Amino Acid Position | Feature |
| --- | --- | --- |
| 1 | AA8 | Normalized positional residue frequency at helix termini C' (Aurora-Rose |
| 2 | AA8 | Side chain angle theta(AAR) (Levitt 1976) |
| 3 | AA8 | Relative preference value at C-cap (Richardson-Richardson 1988) |
| 4 | AA8 | Normalized frequency of zeta L (Maxfield-Scheraga 1976) |
| 5 | AA8 | Turn propensity scale for transmembrane helices (Monne et al. 1999) |
| 6 | AA5 | Relative preference value at C-cap (Richardson-Richardson 1988) |
| 7 | AA5 | Turn propensity scale for transmembrane helices (Monne et al. 1999) |
| 8 | AA5 | Normalized positional residue frequency at helix termini C' (Aurora-Rose |
| 9 | AA5 | Normalized frequency of left-handed alpha-helix (Maxfield-Scheraga 1976) |
| 10 | AA11 | Normalized frequency of left-handed alpha-helix (Maxfield-Scheraga 1976) |
| 11 | AA11 | Thermodynamic beta sheet propensity (Kim-Berg 1993) |
| 12 | AA11 | Relative preference value at C-cap (Richardson-Richardson 1988) |
| 13 | AA11 | Normalized positional residue frequency at helix termini C' (Aurora-Rose |
| 14 | AA11 | Side chain angle theta(AAR) (Levitt 1976) |
| 15 | AA11 | Normalized frequency of left-handed helix (Tanaka-Scheraga 1977) |
| 16 | AA11 | Ratio of average and computed composition (Nakashima et al. 1990) |
| 17 | AA2 | Normalized frequency of left-handed alpha-helix (Maxfield-Scheraga 1976) |
| 18 | AA11 | Average relative fractional occurrence in AL(i-1) (Rackovsky-Scheraga 1982) |
| 19 | AA11 | Normalized relative frequency of coil (Isogai et al. 1980) |
| 20 | AA11 | Normalized frequency of zeta L (Maxfield-Scheraga 1976) |
| 21 | AA11 | Normalized frequency of coil (Tanaka-Scheraga 1977) |
| 22 | AA11 | Alpha-helix propensity derived from designed sequences (Koehl-Levitt 1999) |
| 23 | AA2 | Average relative fractional occurrence in AL(i-1) (Rackovsky-Scheraga 1982) |
| 24 | AA11 | Average relative fractional occurrence in ER(i) (Rackovsky-Scheraga 1982) |
| 25 | AA11 | Average relative probability of beta-sheet (Kanehisa-Tsong 1980) |
| 26 | AA2 | Normalized frequency of coil (Tanaka-Scheraga 1977) |
| 27 | AA2 | Ratio of average and computed composition (Nakashima et al. 1990) |
| 28 | AA2 | Normalized relative frequency of coil (Isogai et al. 1980) |
| 29 | AA11 | RF rank (Zimmerman et al. 1968) |
| 30 | AA2 | Alpha-helix propensity derived from designed sequences (Koehl-Levitt 1999) |
| 31 | AA2 | Relative preference value at C-cap (Richardson-Richardson 1988) |
| 32 | AA2 | Normalized frequency of zeta L (Maxfield-Scheraga 1976) |
| 33 | AA11 | Side chain hydropathy corrected for solvation (Roseman 1988) |
| 34 | AA8 | Side chain hydropathy corrected for solvation (Roseman 1988) |
| 35 | AA2 | Normalized positional residue frequency at helix termini C' (Aurora-Rose |
| 36 | AA2 | Side chain angle theta(AAR) (Levitt 1976) |
| 37 | AA8 | Interior composition of amino acids in intracellular proteins of mesophiles |
| 38 | AA11 | Normalized frequency of beta-sheet from CF (Palau et al. 1981) |
| 39 | AA2 | Thermodynamic beta sheet propensity (Kim-Berg 1993) |
| 40 | AA11 | Normalized frequency of beta-sheet (Chou-Fasman 1978b) |
| 41 | AA2 | Beta-strand indices for beta-proteins (Geisow-Roberts 1980) |
| 42 | AA8 | Average membrane preference: AMP07 (Degli Esposti et al. 1990) |
| 43 | AA8 | Direction of hydrophobic moment (Eisenberg-McLachlan 1986) |
| 44 | AA11 | Hydrophobicity scales (Ponnuswamy 1993) |
| 45 | AA11 | Average relative probability of inner beta-sheet (Kanehisa-Tsong 1980) |
| 46 | AA5 | Normalized positional residue frequency at helix termini C4' (Aurora-Rose |
| 47 | AA11 | PSSM-G |
| 48 | AA11 | Beta-strand indices for alpha/beta-proteins (Geisow-Roberts 1980) |
| 49 | AA11 | Information measure for pleated-sheet (Robson-Suzuki 1976) |
| 50 | AA11 | Beta-strand indices for beta-proteins (Geisow-Roberts 1980) |
| 51 | AA11 | Average membrane preference: AMP07 (Degli Esposti et al. 1990) |
| 52 | AA8 | A parameter of charge transfer capability (Charton-Charton 1983) |
| 53 | AA5 | Side chain angle theta(AAR) (Levitt 1976) |
| 54 | AA2 | PSSM-G |
| 55 | AA11 | Hydropathy scale based on self-information values in the two-state model (16% |
| 56 | AA11 | Conformational parameter of beta-structure (Beghin-Dirkx 1975) |
| 57 | AA8 | 8 A contact number (Nishikawa-Ooi 1980) |
| 58 | AA8 | Beta-strand indices for beta-proteins (Geisow-Roberts 1980) |
| 59 | AA11 | Conformational preference for antiparallel beta-strands (Lifson-Sander 1979) |
| 60 | AA11 | Propensity of amino acids within pi-helices (Fodje-Al-Karadaghi 2002) |
| 61 | AA8 | Hydrophobic parameter (Levitt 1976) |
| 62 | AA8 | Ratio of buried and accessible molar fractions (Janin 1979) |
| 63 | AA8 | Percentage of buried residues (Janin et al. 1978) |
| 64 | AA11 | Normalized frequency of beta-sheet (Crawford et al. 1973) |
| 65 | AA8 | Membrane preference for cytochrome b: MPH89 (Degli Esposti et al. 1990) |
| 66 | AA2 | Interior composition of amino acids in intracellular proteins of mesophiles |
| 67 | AA8 | Conformational parameter of beta-structure (Beghin-Dirkx 1975) |
| 68 | AA11 | Direction of hydrophobic moment (Eisenberg-McLachlan 1986) |
| 69 | AA8 | Hydropathy scale based on self-information values in the two-state model (5% |
| 70 | AA8 | Hydrophobicity scales (Ponnuswamy 1993) |
| 71 | AA11 | Free energy in beta-strand conformation (Munoz-Serrano 1994) |
| 72 | AA11 | Weights for beta-sheet at the window position of -1 (Qian-Sejnowski 1988) |
| 73 | AA8 | Average relative fractional occurrence in AL(i) (Rackovsky-Scheraga 1982) |
| 74 | AA5 | Membrane preference for cytochrome b: MPH89 (Degli Esposti et al. 1990) |
| 75 | AA8 | Flexibility parameter for one rigid neighbor (Karplus-Schulz 1985) |
| 76 | AA11 | Weights for coil at the window position of -2 (Qian-Sejnowski 1988) |
| 77 | AA11 | Ratio of buried and accessible molar fractions (Janin 1979) |
| 78 | AA8 | Proportion of residues 95% buried (Chothia 1976) |
| 79 | AA11 | Aperiodic indices for beta-proteins (Geisow-Roberts 1980) |
| 80 | AA5 | Weights for beta-sheet at the window position of 3 (Qian-Sejnowski 1988) |
| 81 | AA11 | 8 A contact number (Nishikawa-Ooi 1980) |
| 82 | AA5 | A parameter of charge transfer capability (Charton-Charton 1983) |
| 83 | AA8 | Hydropathy scale based on self-information values in the two-state model (36% |
| 84 | AA11 | Transmembrane regions of non-mt-proteins (Nakashima et al. 1990) |
| 85 | AA11 | The Chou-Fasman parameter of the coil conformation (Charton-Charton 1983) |
| 86 | AA11 | Weights for coil at the window position of 1 (Qian-Sejnowski 1988) |
| 87 | AA11 | Weights for beta-sheet at the window position of 0 (Qian-Sejnowski 1988) |
| 88 | AA11 | Graph shape index (Fauchere et al. 1988) |
| 89 | AA8 | Weights for beta-sheet at the window position of -1 (Qian-Sejnowski 1988) |
| 90 | AA5 | Normalized frequency of left-handed helix (Tanaka-Scheraga 1977) |
| 91 | AA8 | Transmembrane regions of non-mt-proteins (Nakashima et al. 1990) |
| 92 | AA11 | Free energy change of epsilon(i) to epsilon(ex) (Wertz-Scheraga 1978) |
| 93 | AA11 | Average relative fractional occurrence in AL(i) (Rackovsky-Scheraga 1982) |
| 94 | AA11 | Normalized frequency of beta-sheet in alpha/beta class (Palau et al. 1981) |
| 95 | AA11 | Weights for beta-sheet at the window position of 1 (Qian-Sejnowski 1988) |
| 96 | AA11 | Hydropathy scale based on self-information values in the two-state model (36% |
| 97 | AA11 | p-Values of mesophilic proteins based on the distributions of B values |
| 98 | AA8 | Linker propensity from small dataset (linker length is less than six |
| 99 | AA11 | Interior composition of amino acids in intracellular proteins of mesophiles |
| 100 | AA8 | AA composition of MEM of multi-spanning proteins (Nakashima-Nishikawa 1992) |
| 101 | AA2 | Transmembrane regions of non-mt-proteins (Nakashima et al. 1990) |
| 102 | AA11 | Weights for beta-sheet at the window position of -5 (Qian-Sejnowski 1988) |
| 103 | AA8 | Weights for coil at the window position of 5 (Qian-Sejnowski 1988) |
| 104 | AA11 | Long range non-bonded energy per atom (Oobatake-Ooi 1977) |
| 105 | AA2 | Hydrophobic parameter (Levitt 1976) |
| 106 | AA11 | Conformational preference for all beta-strands (Lifson-Sander 1979) |
| 107 | AA11 | Normalized frequency of beta-sheet unweighted (Levitt 1978) |
| 108 | AA11 | Aperiodic indices for alpha/beta-proteins (Geisow-Roberts 1980) |
| 109 | AA11 | Side chain orientational preference (Rackovsky-Scheraga 1977) |
| 110 | AA8 | p-Values of mesophilic proteins based on the distributions of B values |
| 111 | AA5 | Interior composition of amino acids in intracellular proteins of mesophiles |
| 112 | AA11 | Entire chain composition of amino acids in intracellular proteins of |
| 113 | AA11 | Frequency of the 4th residue in turn (Chou-Fasman 1978b) |
| 114 | AA11 | Hydropathy scale based on self-information values in the two-state model (25% |
| 115 | AA2 | Normalized frequency of beta-sheet from LG (Palau et al. 1981) |
| 116 | AA11 | Bulkiness (Zimmerman et al. 1968) |
| 117 | AA8 | Weights for beta-sheet at the window position of -5 (Qian-Sejnowski 1988) |
| 118 | AA2 | Average membrane preference: AMP07 (Degli Esposti et al. 1990) |
| 119 | AA8 | Hydropathy index (Kyte-Doolittle 1982) |
| 120 | AA8 | Weights for beta-sheet at the window position of 0 (Qian-Sejnowski 1988) |
| 121 | AA8 | Free energy in beta-strand conformation (Munoz-Serrano 1994) |
| 122 | AA8 | Average relative probability of inner beta-sheet (Kanehisa-Tsong 1980) |
| 123 | AA11 | Hydropathy scale based on self-information values in the two-state model (20% |
| 124 | AA5 | Weights for beta-sheet at the window position of -6 (Qian-Sejnowski 1988) |
| 125 | AA5 | Average relative fractional occurrence in EL(i-1) (Rackovsky-Scheraga 1982) |
| 126 | AA8 | Hydropathy scale based on self-information values in the two-state model (20% |
| 127 | AA8 | Hydropathy scale based on self-information values in the two-state model (16% |
| 128 | AA11 | Hydrophobicity (Jones 1975) |
| 129 | AA11 | Hydrophobicity index (Argos et al. 1982) |
| 130 | AA11 | Side-chain contribution to protein stability (kJ/mol) (Takano-Yutani 2001) |
| 131 | AA8 | Normalized frequency of left-handed helix (Tanaka-Scheraga 1977) |
| 132 | AA8 | Hydration free energy (Robson-Osguthorpe 1979) |
| 133 | AA11 | Normalized hydrophobicity scales for alpha/beta-proteins (Cid et al. 1992) |
| 134 | AA2 | Normalized frequency of extended structure (Maxfield-Scheraga 1976) |
| 135 | AA2 | Side chain hydropathy corrected for solvation (Roseman 1988) |
| 136 | AA11 | Linker index (Bae et al. 2005) |
| 137 | AA11 | Side chain interaction parameter (Krigbaum-Komoriya 1979) |
| 138 | AA8 | Interior composition of amino acids in nuclear proteins (percent) |
| 139 | AA8 | Average relative fractional occurrence in EL(i-1) (Rackovsky-Scheraga 1982) |
| 140 | AA3 | PSSM-S |
| 141 | AA8 | Knowledge-based membrane-propensity scale from 3D_Helix in MPtopo databases |
| 142 | AA11 | Mean fractional area loss (Rose et al. 1985) |
| 143 | AA11 | Normalized relative frequency of bend R (Isogai et al. 1980) |
| 144 | AA2 | Weights for beta-sheet at the window position of -1 (Qian-Sejnowski 1988) |
| 145 | AA8 | Hydrophobic parameter pi (Fauchere-Pliska 1983) |
| 146 | AA8 | Mean fractional area loss (Rose et al. 1985) |
| 147 | AA5 | Hydropathy scale based on self-information values in the two-state model (5% |
| 148 | AA8 | Modified Kyte-Doolittle hydrophobicity scale (Juretic et al. 1998) |
| 149 | AA2 | AA composition of MEM of multi-spanning proteins (Nakashima-Nishikawa 1992) |
| 150 | AA8 | Entire chain composition of amino acids in intracellular proteins of |
| 151 | AA8 | Normalized frequency of beta-sheet (Chou-Fasman 1978b) |
| 152 | AA8 | Apparent partial specific volume (Bull-Breese 1974) |
| 153 | AA11 | Information value for accessibility; average fraction 23% (Biou et al. 1988) |
| 154 | AA8 | AA composition of MEM of single-spanning proteins (Nakashima-Nishikawa 1992) |
| 155 | AA2 | Normalized frequency of beta-sheet unweighted (Levitt 1978) |
| 156 | AA11 | Polar requirement (Woese 1973) |
| 157 | AA11 | Polarity (Grantham 1974) |
| 158 | AA11 | A parameter of charge transfer capability (Charton-Charton 1983) |
| 159 | AA11 | Normalized frequency of extended structure (Maxfield-Scheraga 1976) |
| 160 | AA8 | Average gain ratio in surrounding hydrophobicity (Ponnuswamy et al. 1980) |
| 161 | AA11 | Information measure for coil (Robson-Suzuki 1976) |
| 162 | AA5 | Weights for beta-sheet at the window position of -2 (Qian-Sejnowski 1988) |
| 163 | AA11 | Normalized frequency of C-terminal beta-sheet (Chou-Fasman 1978b) |
| 164 | AA11 | Normalized frequency of beta-sheet with weights (Levitt 1978) |
| 165 | AA11 | Relative frequency in beta-sheet (Prabhakaran 1990) |
| 166 | AA9 | PSSM-E |
| 167 | AA8 | Solvation free energy (Eisenberg-McLachlan 1986) |
| 168 | AA5 | Frequency of the 1st residue in turn (Chou-Fasman 1978b) |
| 169 | AA2 | Normalized positional residue frequency at helix termini N4 (Aurora-Rose |
| 170 | AA2 | Normalized frequency of beta-sheet (Chou-Fasman 1978b) |
| 171 | AA8 | Normalized positional residue frequency at helix termini N4 (Aurora-Rose |
| 172 | AA8 | Long range non-bonded energy per atom (Oobatake-Ooi 1977) |
| 173 | AA11 | Average reduced distance for side chain (Meirovitch et al. 1980) |
| 174 | AA11 | Hydrophobic parameter pi (Fauchere-Pliska 1983) |
| 175 | AA5 | Weights for alpha-helix at the window position of 3 (Qian-Sejnowski 1988) |
| 176 | AA8 | Normalized frequency of extended structure (Burgess et al. 1974) |
| 177 | AA11 | Membrane preference for cytochrome b: MPH89 (Degli Esposti et al. 1990) |
| 178 | AA11 | Normalized frequency of beta-sheet in alpha+beta class (Palau et al. 1981) |
| 179 | AA8 | PSSM-G |
| 180 | AA11 | Linker propensity from small dataset (linker length is less than six |
| 181 | AA2 | Solvation free energy (Eisenberg-McLachlan 1986) |
| 182 | AA11 | Normalized hydrophobicity scales for beta-proteins (Cid et al. 1992) |
| 183 | AA11 | Modified Kyte-Doolittle hydrophobicity scale (Juretic et al. 1998) |
| 184 | AA11 | Normalized positional residue frequency at helix termini N"' (Aurora-Rose |
| 185 | AA5 | Weights for coil at the window position of 5 (Qian-Sejnowski 1988) |
| 186 | AA9 | PSSM-R |
| 187 | AA8 | Normalized frequency of beta-sheet from LG (Palau et al. 1981) |
| 188 | AA8 | Polarity (Grantham 1974) |
| 189 | AA8 | Normalized frequency of extended structure (Maxfield-Scheraga 1976) |
| 190 | AA8 | Polar requirement (Woese 1973) |
| 191 | AA8 | Normalized positional residue frequency at helix termini Nc (Aurora-Rose |
| 192 | AA6 | PSSM-D |
| 193 | AA8 | Information value for accessibility; average fraction 23% (Biou et al. 1988) |
| 194 | AA5 | Weights for coil at the window position of -1 (Qian-Sejnowski 1988) |
| 195 | AA8 | Normalized frequency of chain reversal S (Tanaka-Scheraga 1977) |
| 196 | AA8 | AA composition of membrane proteins (Nakashima et al. 1990) |
| 197 | AA8 | Side chain hydropathy uncorrected for solvation (Roseman 1988) |
| 198 | AA9 | PSSM-K |
| 199 | AA11 | 14 A contact number (Nishikawa-Ooi 1986) |
| 200 | AA8 | Normalized frequency of extended structure (Tanaka-Scheraga 1977) |
| 201 | AA2 | 8 A contact number (Nishikawa-Ooi 1980) |
| 202 | AA8 | Frequency of the 1st residue in turn (Chou-Fasman 1978b) |
| 203 | AA8 | RF rank (Zimmerman et al. 1968) |
| 204 | AA11 | Interactivity scale obtained by maximizing the mean of correlation |
| 205 | AA8 | Information value for accessibility; average fraction 35% (Biou et al. 1988) |
| 206 | AA11 | Hydropathy scale based on self-information values in the two-state model (9% |
| 207 | AA3 | PSSM-E |
| 208 | AA11 | Conformational parameter of inner helix (Beghin-Dirkx 1975) |
| 209 | AA8 | Transfer free energy from chx to wat (Radzicka-Wolfenden 1988) |
| 210 | AA11 | Information value for accessibility; average fraction 35% (Biou et al. 1988) |
| 211 | AA2 | Normalized frequency of beta-sheet from CF (Palau et al. 1981) |
| 212 | AA11 | Aperiodic indices (Geisow-Roberts 1980) |
| 213 | AA8 | Weights for alpha-helix at the window position of 2 (Qian-Sejnowski 1988) |
| 214 | AA11 | Normalized positional residue frequency at helix termini Cc (Aurora-Rose |
| 215 | AA8 | Hydrophilicity value (Hopp-Woods 1981) |
| 216 | AA11 | Optimal matching hydrophobicity (Sweet-Eisenberg 1983) |
| 217 | AA11 | Normalized frequency of N-terminal beta-sheet (Chou-Fasman 1978b) |
| 218 | AA8 | Normalized frequency of beta-sheet (Crawford et al. 1973) |
| 219 | AA5 | Interior composition of amino acids in extracellular proteins of mesophiles |
| 220 | AA11 | Transfer free energy CHP/water (Lawson et al. 1984) |
| 221 | AA12 | PSSM-T |
| 222 | AA2 | Membrane preference for cytochrome b: MPH89 (Degli Esposti et al. 1990) |
| 223 | AA8 | Knowledge-based membrane-propensity scale from 1D_Helix in MPtopo databases |
| 224 | AA5 | Normalized positional residue frequency at helix termini N4 (Aurora-Rose |
| 225 | AA8 | Hydropathy scale based on self-information values in the two-state model (50% |
| 226 | AA10 | PSSM-E |
| 227 | AA8 | Average gain in surrounding hydrophobicity (Ponnuswamy et al. 1980) |
| 228 | AA9 | PSSM-Q |
| 229 | AA11 | Relative frequency in reverse-turn (Prabhakaran 1990) |
| 230 | AA11 | Normalized frequency of reverse turn with weights (Levitt 1978) |
| 231 | AA11 | Normalized frequency of reverse turn unweighted (Levitt 1978) |
| 232 | AA11 | Turn propensity scale for transmembrane helices (Monne et al. 1999) |
| 233 | AA2 | Average relative fractional occurrence in E0(i-1) (Rackovsky-Scheraga 1982) |
| 234 | AA5 | Average relative fractional occurrence in AL(i) (Rackovsky-Scheraga 1982) |
| 235 | AA8 | Consensus normalized hydrophobicity scale (Eisenberg 1984) |
| 236 | AA2 | Weights for coil at the window position of -1 (Qian-Sejnowski 1988) |
| 237 | AA8 | Composition of amino acids in extracellular proteins (percent) (Cedano et |
| 238 | AA5 | Conformational parameter of beta-structure (Beghin-Dirkx 1975) |
| 239 | AA8 | 14 A contact number (Nishikawa-Ooi 1986) |
| 240 | AA8 | Average relative fractional occurrence in AL(i-1) (Rackovsky-Scheraga 1982) |
| 241 | AA8 | Hydrophobicity coefficient in RP-HPLC C8 with 0.1%TFA/MeCN/H2O (Wilce et al. |
| 242 | AA5 | Alpha-helix indices for beta-proteins (Geisow-Roberts 1980) |
| 243 | AA8 | Weights for alpha-helix at the window position of 6 (Qian-Sejnowski 1988) |
| 244 | AA11 | Average gain ratio in surrounding hydrophobicity (Ponnuswamy et al. 1980) |
| 245 | AA8 | Average relative fractional occurrence in AR(i-1) (Rackovsky-Scheraga 1982) |
| 246 | AA5 | Normalized positional residue frequency at helix termini Nc (Aurora-Rose |
| 247 | AA5 | Average membrane preference: AMP07 (Degli Esposti et al. 1990) |
| 248 | AA5 | Normalized positional residue frequency at helix termini N'(Aurora-Rose |
| 249 | AA11 | Optimized average non-bonded energy per atom (Oobatake et al. 1985) |
| 250 | AA11 | Weights for coil at the window position of 0 (Qian-Sejnowski 1988) |
| 251 | AA8 | Weights for coil at the window position of -2 (Qian-Sejnowski 1988) |
| 252 | AA8 | Normalized positional residue frequency at helix termini Cc (Aurora-Rose |
| 253 | AA11 | Normalized flexibility parameters (B-values) average (Vihinen et al. 1994) |
| 254 | AA11 | Retention coefficient in HPLC pH2.1 (Meek 1980) |
| 255 | AA8 | Side chain orientational preference (Rackovsky-Scheraga 1977) |
| 256 | AA11 | Normalized relative frequency of bend S (Isogai et al. 1980) |
| 257 | AA2 | Normalized frequency of chain reversal S (Tanaka-Scheraga 1977) |
| 258 | AA5 | Principal property value z3 (Wold et al. 1987) |
| 259 | AA8 | Composition (Grantham 1974) |
| 260 | AA11 | Conformational preference for parallel beta-strands (Lifson-Sander 1979) |
| 261 | AA8 | Weights for beta-sheet at the window position of 6 (Qian-Sejnowski 1988) |
| 262 | AA8 | Normalized frequency of N-terminal beta-sheet (Chou-Fasman 1978b) |
| 263 | AA5 | Value of theta(i-1) (Rackovsky-Scheraga 1982) |
| 264 | AA11 | AA composition of MEM of multi-spanning proteins (Nakashima-Nishikawa 1992) |
| 265 | AA9 | PSSM-H |
| 266 | AA9 | PSSM-P |
| 267 | AA11 | Hydrophilicity value (Hopp-Woods 1981) |
| 268 | AA11 | Hydrophobic parameter (Levitt 1976) |
| 269 | AA8 | Optimized beta-structure-coil equilibrium constant (Oobatake et al. 1985) |
| 270 | AA11 | Relative preference value at C4 (Richardson-Richardson 1988) |
| 271 | AA2 | Normalized positional residue frequency at helix termini N3 (Aurora-Rose |
| 272 | AA8 | Interior composition of amino acids in intracellular proteins of thermophiles |
| 273 | AA11 | Normalized frequency of the 2nd and 3rd residues in turn (Chou-Fasman 1978b) |
| 274 | AA8 | Weights for alpha-helix at the window position of 0 (Qian-Sejnowski 1988) |
| 275 | AA11 | Weights for coil at the window position of 2 (Qian-Sejnowski 1988) |
| 276 | AA8 | Transfer free energy to lipophilic phase (von Heijne-Blomberg 1979) |
| 277 | AA5 | Weights for coil at the window position of -2 (Qian-Sejnowski 1988) |
| 278 | AA5 | Hydropathy scale based on self-information values in the two-state model (50% |
| 279 | AA5 | Relative mutability (Dayhoff et al. 1978a) |
| 280 | AA8 | Normalized flexibility parameters (B-values) average (Vihinen et al. 1994) |
| 281 | AA8 | Average relative fractional occurrence in A0(i) (Rackovsky-Scheraga 1982) |
| 282 | AA5 | Ratio of average and computed composition (Nakashima et al. 1990) |
| 283 | AA5 | Normalized relative frequency of alpha-helix (Isogai et al. 1980) |
| 284 | AA11 | Normalized frequency of beta-sheet in all-beta class (Palau et al. 1981) |
| 285 | AA2 | Relative frequency in beta-sheet (Prabhakaran 1990) |
| 286 | AA2 | Normalized frequency of beta-sheet with weights (Levitt 1978) |
| 287 | AA8 | Principal property value z3 (Wold et al. 1987) |
| 288 | AA8 | Principal property value z1 (Wold et al. 1987) |
| 289 | AA5 | Weights for alpha-helix at the window position of 0 (Qian-Sejnowski 1988) |
| 290 | AA8 | Surrounding hydrophobicity in turn (Ponnuswamy et al. 1980) |
| 291 | AA8 | Side chain interaction parameter (Krigbaum-Komoriya 1979) |
| 292 | AA11 | Proportion of residues 95% buried (Chothia 1976) |
| 293 | AA5 | Hydration free energy (Robson-Osguthorpe 1979) |
| 294 | AA8 | Normalized frequency of left-handed alpha-helix (Maxfield-Scheraga 1976) |
| 295 | AA5 | Composition of amino acids in extracellular proteins (percent) (Cedano et |
| 296 | AA2 | Weights for beta-sheet at the window position of -2 (Qian-Sejnowski 1988) |
| 297 | AA8 | Frequency of occurrence in beta-bends (Lewis et al. 1971) |
| 298 | AA8 | Average number of surrounding residues (Ponnuswamy et al. 1980) |
| 299 | AA2 | RF rank (Zimmerman et al. 1968) |
| 300 | AA8 | Weights for alpha-helix at the window position of 3 (Qian-Sejnowski 1988) |
| 301 | AA2 | Average relative probability of beta-sheet (Kanehisa-Tsong 1980) |
| 302 | AA2 | Average relative fractional occurrence in EL(i-1) (Rackovsky-Scheraga 1982) |
| 303 | AA5 | Free energy in alpha-helical region (Munoz-Serrano 1994) |
| 304 | AA8 | Alpha-helix indices for beta-proteins (Geisow-Roberts 1980) |
| 305 | AA8 | Normalized frequency of beta-sheet in all-beta class (Palau et al. 1981) |
| 306 | AA8 | Relative population of conformational state C (Vasquez et al. 1983) |
| 307 | AA11 | Transfer free energy from oct to wat (Radzicka-Wolfenden 1988) |
| 308 | AA5 | Average relative fractional occurrence in EL(i) (Rackovsky-Scheraga 1982) |
| 309 | AA9 | PSSM-V |
| 310 | AA3 | Relative preference value at N-cap (Richardson-Richardson 1988) |
| 311 | AA5 | Side chain hydropathy corrected for solvation (Roseman 1988) |
| 312 | AA2 | Free energy in beta-strand conformation (Munoz-Serrano 1994) |
| 313 | AA11 | Normalized frequency of extended structure (Burgess et al. 1974) |
| 314 | AA5 | Average relative probability of inner helix (Kanehisa-Tsong 1980) |
| 315 | AA3 | Weights for coil at the window position of -3 (Qian-Sejnowski 1988) |
| 316 | AA11 | Normalized frequency of extended structure (Tanaka-Scheraga 1977) |
| 317 | AA8 | Surrounding hydrophobicity in folded form (Ponnuswamy et al. 1980) |
| 318 | AA11 | Normalized hydrophobicity scales for alpha-proteins (Cid et al. 1992) |
| 319 | AA5 | Normalized relative frequency of coil (Isogai et al. 1980) |
| 320 | AA11 | Normalized relative frequency of extended structure (Isogai et al. 1980) |
| 321 | AA11 | Hydropathy scale based on self-information values in the two-state model (50% |
| 322 | AA11 | Average reduced distance for side chain (Rackovsky-Scheraga 1977) |
| 323 | AA8 | Weights for beta-sheet at the window position of -2 (Qian-Sejnowski 1988) |
| 324 | AA3 | PSSM-Q |
| 325 | AA6 | PSSM-E |
| 326 | AA8 | Aperiodic indices for alpha/beta-proteins (Geisow-Roberts 1980) |
| 327 | AA5 | Relative population of conformational state C (Vasquez et al. 1983) |
| 328 | AA11 | Information measure for middle helix (Robson-Suzuki 1976) |
| 329 | AA11 | Average relative fractional occurrence in EL(i-1) (Rackovsky-Scheraga 1982) |
| 330 | AA5 | Beta-strand indices for beta-proteins (Geisow-Roberts 1980) |
| 331 | AA8 | Aperiodic indices for alpha-proteins (Geisow-Roberts 1980) |
| 332 | AA2 | Normalized frequency of beta-sheet (Crawford et al. 1973) |
| 333 | AA11 | Mean polarity (Radzicka-Wolfenden 1988) |
| 334 | AA11 | Relative preference value at Mid (Richardson-Richardson 1988) |
| 335 | AA5 | Average relative fractional occurrence in AR(i-1) (Rackovsky-Scheraga 1982) |
| 336 | AA11 | Retention coefficient in NaClO4 (Meek-Rossetti 1981) |
| 337 | AA5 | Average relative probability of helix (Kanehisa-Tsong 1980) |
| 338 | AA8 | Aperiodic indices (Geisow-Roberts 1980) |
| 339 | AA2 | Turn propensity scale for transmembrane helices (Monne et al. 1999) |
| 340 | AA2 | Average relative fractional occurrence in AL(i) (Rackovsky-Scheraga 1982) |
| 341 | AA2 | Beta-strand indices for alpha/beta-proteins (Geisow-Roberts 1980) |
| 342 | AA5 | Surrounding hydrophobicity in alpha-helix (Ponnuswamy et al. 1980) |
| 343 | AA11 | Propensity to be buried inside (Wertz-Scheraga 1978) |
| 344 | AA8 | Average surrounding hydrophobicity (Manavalan-Ponnuswamy 1978) |
| 345 | AA8 | AA composition of mt-proteins from animal (Nakashima et al. 1990) |
| 346 | AA9 | PSSM-L |
| 347 | AA8 | Weights for alpha-helix at the window position of 4 (Qian-Sejnowski 1988) |
| 348 | AA11 | Normalized hydrophobicity scales for alpha+beta-proteins (Cid et al. 1992) |
| 349 | AA2 | A parameter of charge transfer capability (Charton-Charton 1983) |
| 350 | AA11 | Average surrounding hydrophobicity (Manavalan-Ponnuswamy 1978) |
| 351 | AA8 | Normalized frequency of beta-sheet in alpha+beta class (Palau et al. 1981) |
| 352 | AA8 | Optimized side chain interaction parameter (Oobatake et al. 1985) |
| 353 | AA8 | Principal component IV (Sneath 1966) |
| 354 | AA11 | Surface composition of amino acids in extracellular proteins of mesophiles |
| 355 | AA8 | Relative frequency in beta-sheet (Prabhakaran 1990) |
| 356 | AA8 | Normalized frequency of beta-sheet with weights (Levitt 1978) |
| 357 | AA8 | AA composition of mt-proteins from fungi and plant (Nakashima et al. 1990) |
| 358 | AA11 | Average number of surrounding residues (Ponnuswamy et al. 1980) |
| 359 | AA2 | The Chou-Fasman parameter of the coil conformation (Charton-Charton 1983) |
| 360 | AA5 | AA composition of MEM of multi-spanning proteins (Nakashima-Nishikawa 1992) |
| 361 | AA8 | Weights for coil at the window position of -1 (Qian-Sejnowski 1988) |
| 362 | AA6 | PSSM-P |
| 363 | AA5 | Normalized positional residue frequency at helix termini N5 (Aurora-Rose |
| 364 | AA5 | Normalized positional residue frequency at helix termini C1 (Aurora-Rose |
| 365 | AA5 | Normalized frequency of beta-sheet (Chou-Fasman 1978b) |
| 366 | AA11 | Average relative fractional occurrence in AR(i-1) (Rackovsky-Scheraga 1982) |
| 367 | AA2 | Weights for beta-sheet at the window position of 0 (Qian-Sejnowski 1988) |
| 368 | AA8 | Weights for beta-sheet at the window position of -6 (Qian-Sejnowski 1988) |
| 369 | AA11 | Relative preference value at N4 (Richardson-Richardson 1988) |
| 370 | AA8 | Weights for beta-sheet at the window position of 2 (Qian-Sejnowski 1988) |
| 371 | AA11 | Normalized frequency of beta-sheet from LG (Palau et al. 1981) |
| 372 | AA6 | PSSM-Q |
| 373 | AA8 | Information measure for pleated-sheet (Robson-Suzuki 1976) |
| 374 | AA2 | Information measure for pleated-sheet (Robson-Suzuki 1976) |
| 375 | AA8 | Average relative probability of beta-sheet (Kanehisa-Tsong 1980) |
| 376 | AA2 | Helix-coil equilibrium constant (Ptitsyn-Finkelstein 1983) |
| 377 | AA12 | PSSM-G |
| 378 | AA11 | Normalized frequency of beta-structure (Nagano 1973) |
| 379 | AA8 | Normalized relative frequency of bend S (Isogai et al. 1980) |
| 380 | AA8 | Normalized frequency of beta-sheet unweighted (Levitt 1978) |
| 381 | AA2 | Normalized positional residue frequency at helix termini N'(Aurora-Rose |
| 382 | AA5 | Composition of amino acids in nuclear proteins (percent) (Cedano et al. |
| 383 | AA8 | Information measure for coil (Robson-Suzuki 1976) |
| 384 | AA8 | Frequency of the 3rd residue in turn (Chou-Fasman 1978b) |
| 385 | AA11 | Fraction of site occupied by water (Krigbaum-Komoriya 1979) |
| 386 | AA8 | Average relative fractional occurrence in E0(i-1) (Rackovsky-Scheraga 1982) |
| 387 | AA2 | Weights for alpha-helix at the window position of 0 (Qian-Sejnowski 1988) |
| 388 | AA3 | PSSM-D |
| 389 | AA8 | Optimized average non-bonded energy per atom (Oobatake et al. 1985) |
| 390 | AA2 | Percentage of buried residues (Janin et al. 1978) |
| 391 | AA2 | Ratio of buried and accessible molar fractions (Janin 1979) |
| 392 | AA11 | Free energy in beta-strand region (Munoz-Serrano 1994) |
| 393 | AA8 | Normalized positional residue frequency at helix termini N'(Aurora-Rose |
| 394 | AA8 | Hydropathy scale based on self-information values in the two-state model (9% |
| 395 | AA9 | PSSM-D |
| 396 | AA11 | Hydropathy scale based on self-information values in the two-state model (5% |
| 397 | AA8 | The number of atoms in the side chain labelled 2+1 (Charton-Charton 1983) |
| 398 | AA11 | Effective partition energy (Miyazawa-Jernigan 1985) |
| 399 | AA11 | Interactivity scale obtained from the contact matrix (Bastolla et al. 2005) |
| 400 | AA5 | Frequency of occurrence in beta-bends (Lewis et al. 1971) |
| 401 | AA8 | Hydrophobicity index (Argos et al. 1982) |
| 402 | AA8 | Hydrophobicity (Jones 1975) |
| 403 | AA8 | Relative preference value at C" (Richardson-Richardson 1988) |
| 404 | AA2 | Relative preference value at Mid (Richardson-Richardson 1988) |
| 405 | AA2 | Weights for coil at the window position of -2 (Qian-Sejnowski 1988) |
| 406 | AA5 | Normalized frequency of alpha region (Maxfield-Scheraga 1976) |
| 407 | AA11 | Average reduced distance for C-alpha (Meirovitch et al. 1980) |
| 408 | AA8 | Normalized frequency of beta-sheet in alpha/beta class (Palau et al. 1981) |
| 409 | AA8 | Conformational preference for all beta-strands (Lifson-Sander 1979) |
| 410 | AA11 | Relative population of conformational state C (Vasquez et al. 1983) |
| 411 | AA5 | 8 A contact number (Nishikawa-Ooi 1980) |
| 412 | AA11 | The stability scale from the knowledge-based atom-atom potential (Zhou-Zhou |
| 413 | AA11 | Buriability (Zhou-Zhou 2004) |
| 414 | AA5 | Ratio of buried and accessible molar fractions (Janin 1979) |
| 415 | AA11 | Side chain interaction parameter (Krigbaum-Rubin 1971) |
| 416 | AA2 | Hydropathy scale based on self-information values in the two-state model (16% |
| 417 | AA5 | Normalized frequency of C-terminal helix (Chou-Fasman 1978b) |
| 418 | AA6 | PSSM-T |
| 419 | AA8 | The Chou-Fasman parameter of the coil conformation (Charton-Charton 1983) |
| 420 | AA8 | Ratio of average and computed composition (Nakashima et al. 1990) |
| 421 | AA2 | Normalized frequency of chain reversal D (Tanaka-Scheraga 1977) |
| 422 | AA2 | Principal property value z1 (Wold et al. 1987) |
| 423 | AA8 | Relative preference value at N-cap (Richardson-Richardson 1988) |
| 424 | AA11 | Solvation free energy (Eisenberg-McLachlan 1986) |
| 425 | AA2 | Hydrophobicity scales (Ponnuswamy 1993) |
| 426 | AA5 | Side chain hydropathy uncorrected for solvation (Roseman 1988) |
| 427 | AA8 | Hydropathy scale based on self-information values in the two-state model (25% |
| 428 | AA11 | Relative preference value at N-cap (Richardson-Richardson 1988) |
| 429 | AA4 | PSSM-L |
| 430 | AA8 | Normalized relative frequency of helix end (Isogai et al. 1980) |
| 431 | AA11 | Average gain in surrounding hydrophobicity (Ponnuswamy et al. 1980) |
| 432 | AA11 | HPLC parameter (Parker et al. 1986) |
| 433 | AA11 | Principal property value z1 (Wold et al. 1987) |
| 434 | AA8 | Average side chain orientation angle (Meirovitch et al. 1980) |
| 435 | AA5 | Relative preference value at C" (Richardson-Richardson 1988) |
| 436 | AA8 | Weights for coil at the window position of 3 (Qian-Sejnowski 1988) |
| 437 | AA8 | The Kerr-constant increments (Khanarian-Moore 1980) |
| 438 | AA5 | Normalized frequency of extended structure (Maxfield-Scheraga 1976) |
| 439 | AA8 | Weights for coil at the window position of -3 (Qian-Sejnowski 1988) |
| 440 | AA5 | Percentage of buried residues (Janin et al. 1978) |
| 441 | AA8 | Side chain torsion angle phi(AAAR) (Levitt 1976) |
| 442 | AA11 | Hydrophobicity factor (Goldsack-Chalifoux 1973) |
| 443 | AA11 | Transfer free energy (Simon 1976) Cited by Charton-Charton (1982) |
| 444 | AA11 | Normalized average hydrophobicity scales (Cid et al. 1992) |
| 445 | AA5 | Normalized frequency of the 2nd and 3rd residues in turn (Chou-Fasman 1978b) |
| 446 | AA5 | Information measure for coil (Robson-Suzuki 1976) |
| 447 | AA11 | AA composition of MEM of single-spanning proteins (Nakashima-Nishikawa 1992) |
| 448 | AA11 | Retention coefficient at pH 2 (Guo et al. 1986) |
| 449 | AA2 | Free energy in beta-strand region (Munoz-Serrano 1994) |
| 450 | AA2 | Transfer free energy from chx to wat (Radzicka-Wolfenden 1988) |
| 451 | AA11 | Free energy in beta-strand region (Munoz-Serrano 1994) |
| 452 | AA2 | Hydropathy scale based on self-information values in the two-state model (50% |
| 453 | AA2 | Hydropathy scale based on self-information values in the two-state model (36% |
| 454 | AA11 | Hydropathy index (Kyte-Doolittle 1982) |
| 455 | AA8 | Information measure for middle helix (Robson-Suzuki 1976) |
| 456 | AA8 | Normalized frequency of the 2nd and 3rd residues in turn (Chou-Fasman 1978b) |
| 457 | AA8 | Helix-coil equilibrium constant (Ptitsyn-Finkelstein 1983) |
| 458 | AA2 | Normalized frequency of extended structure (Tanaka-Scheraga 1977) |
| 459 | AA8 | Conformational parameter of inner helix (Beghin-Dirkx 1975) |
| 460 | AA8 | Partial specific volume (Cohn-Edsall 1943) |
| 461 | AA11 | Consensus normalized hydrophobicity scale (Eisenberg 1984) |
| 462 | AA6 | PSSM-R |
| 463 | AA8 | Transfer free energy CHP/water (Lawson et al. 1984) |
| 464 | AA5 | Conformational parameter of inner helix (Beghin-Dirkx 1975) |
| 465 | AA13 | PSSM-R |
| 466 | AA8 | Conformational preference for antiparallel beta-strands (Lifson-Sander 1979) |
| 467 | AA2 | Consensus normalized hydrophobicity scale (Eisenberg 1984) |
| 468 | AA11 | Information measure for C-terminal turn (Robson-Suzuki 1976) |
| 469 | AA8 | Normalized frequency of chain reversal D (Tanaka-Scheraga 1977) |
| 470 | AA5 | Normalized positional residue frequency at helix termini C3 (Aurora-Rose |
| 471 | AA2 | Normalized frequency of C-terminal helix (Chou-Fasman 1978b) |
| 472 | AA11 | N.m.r. chemical shift of alpha-carbon (Fauchere et al. 1988) |
| 473 | AA11 | Accessibility reduction ratio (Ponnuswamy et al. 1980) |
| 474 | AA8 | Composition of amino acids in membrane proteins (percent) (Cedano et al. |
| 475 | AA2 | PSSM-P |
| 476 | AA8 | Normalized hydrophobicity scales for alpha/beta-proteins (Cid et al. 1992) |
| 477 | AA11 | Average reduced distance for C-alpha (Rackovsky-Scheraga 1977) |
| 478 | AA11 | Partition coefficient (Pliska et al. 1981) |
| 479 | AA5 | PSSM-P |
| 480 | AA11 | Entire chain composition of amino acids in intracellular proteins of |
| 481 | AA5 | Linker propensity index (Suyama-Ohara 2003) |
| 482 | AA2 | Weights for beta-sheet at the window position of 2 (Qian-Sejnowski 1988) |
| 483 | AA8 | Weights for alpha-helix at the window position of -1 (Qian-Sejnowski 1988) |
| 484 | AA8 | Normalized relative frequency of coil (Isogai et al. 1980) |
| 485 | AA11 | van der Waals parameter R0 (Levitt 1976) |
| 486 | AA11 | The relative stability scale extracted from mutation experiments (Zhou-Zhou |
| 487 | AA8 | Relative preference value at Mid (Richardson-Richardson 1988) |
| 488 | AA2 | Aperiodic indices (Geisow-Roberts 1980) |
| 489 | AA6 | PSSM-K |
| 490 | AA3 | PSSM-P |
| 491 | AA11 | Interactivity scale obtained by maximizing the mean of correlation |
| 492 | AA8 | Graph shape index (Fauchere et al. 1988) |
| 493 | AA8 | Normalized composition of mt-proteins (Nakashima et al. 1990) |
| 494 | AA8 | Normalized composition from animal (Nakashima et al. 1990) |
| 495 | AA5 | Information measure for C-terminal helix (Robson-Suzuki 1976) |
| 496 | AA11 | Weights for coil at the window position of -1 (Qian-Sejnowski 1988) |
| 497 | AA2 | Normalized frequency of beta-sheet in all-beta class (Palau et al. 1981) |
| 498 | AA8 | Free energy change of epsilon(i) to epsilon(ex) (Wertz-Scheraga 1978) |
| 499 | AA5 | Proportion of residues 95% buried (Chothia 1976) |
| 500 | AA8 | Interior composition of amino acids in extracellular proteins of mesophiles |

**mRMR feature list**

| Order | Amino Acid Position | Feature |
| --- | --- | --- |
| 1 | AA8 | Normalized positional residue frequency at helix termini C' (Aurora-Rose |
| 2 | AA10 | Size (Dawson 1972) |
| 3 | AA3 | Relative preference value at N-cap (Richardson-Richardson 1988) |
| 4 | AA11 | Average reduced distance for C-alpha (Meirovitch et al. 1980) |
| 5 | AA2 | Normalized frequency of beta-sheet from LG (Palau et al. 1981) |
| 6 | AA5 | Normalized positional residue frequency at helix termini C4' (Aurora-Rose |
| 7 | AA9 | PSSM-H |
| 8 | AA11 | RF rank (Zimmerman et al. 1968) |
| 9 | AA13 | PSSM-R |
| 10 | AA8 | Hydropathy scale based on self-information values in the two-state model (36% |
| 11 | AA5 | Side chain angle theta(AAR) (Levitt 1976) |
| 12 | AA3 | PSSM-E |
| 13 | AA5 | Linker propensity index (Suyama-Ohara 2003) |
| 14 | AA8 | Principal component IV (Sneath 1966) |
| 15 | AA4 | Electron-ion interaction potential (Veljkovic et al. 1985) |
| 16 | AA12 | PSSM-T |
| 17 | AA11 | Entire chain composition of amino acids in intracellular proteins of |
| 18 | AA2 | Hydrophobic parameter (Levitt 1976) |
| 19 | AA13 | PSSM-L |
| 20 | AA8 | PSSM-G |
| 21 | AA11 | Normalized relative frequency of bend S (Isogai et al. 1980) |
| 22 | AA5 | Surrounding hydrophobicity in alpha-helix (Ponnuswamy et al. 1980) |
| 23 | AA9 | PSSM-E |
| 24 | AA11 | Graph shape index (Fauchere et al. 1988) |
| 25 | AA8 | Average relative fractional occurrence in AL(i) (Rackovsky-Scheraga 1982) |
| 26 | AA5 | Normalized positional residue frequency at helix termini Nc (Aurora-Rose |
| 27 | AA13 | PSSM-N |
| 28 | AA3 | Weights for coil at the window position of -3 (Qian-Sejnowski 1988) |
| 29 | AA8 | Aperiodic indices for alpha-proteins (Geisow-Roberts 1980) |
| 30 | AA11 | Information measure for extended without H-bond (Robson-Suzuki 1976) |
| 31 | AA5 | Normalized frequency of left-handed helix (Tanaka-Scheraga 1977) |
| 32 | AA6 | PSSM-D |
| 33 | AA1 | AA composition of CYT of single-spanning proteins (Nakashima-Nishikawa 1992) |
| 34 | AA11 | Average relative probability of inner beta-sheet (Kanehisa-Tsong 1980) |
| 35 | AA2 | Normalized positional residue frequency at helix termini N4 (Aurora-Rose |
| 36 | AA8 | Relative preference value at C" (Richardson-Richardson 1988) |
| 37 | AA5 | Weights for beta-sheet at the window position of 3 (Qian-Sejnowski 1988) |
| 38 | AA11 | Surface composition of amino acids in extracellular proteins of mesophiles |
| 39 | AA2 | Normalized frequency of beta-sheet unweighted (Levitt 1978) |
| 40 | AA8 | Normalized frequency of left-handed helix (Tanaka-Scheraga 1977) |
| 41 | AA10 | PSSM-E |
| 42 | AA8 | Normalized positional residue frequency at helix termini Cc (Aurora-Rose |
| 43 | AA5 | Normalized frequency of alpha region (Maxfield-Scheraga 1976) |
| 44 | AA11 | Hydrophobicity (Jones 1975) |
| 45 | AA10 | Weights for alpha-helix at the window position of -4 (Qian-Sejnowski 1988) |
| 46 | AA3 | PSSM-D |
| 47 | AA8 | Normalized relative frequency of helix end (Isogai et al. 1980) |
| 48 | AA2 | Normalized frequency of chain reversal D (Tanaka-Scheraga 1977) |
| 49 | AA8 | Hydrophobicity index (Argos et al. 1982) |
| 50 | AA4 | PSSM-L |
| 51 | AA5 | Relative mutability (Dayhoff et al. 1978a) |
| 52 | AA11 | Normalized frequency of N-terminal beta-sheet (Chou-Fasman 1978b) |
| 53 | AA5 | Relative preference value at C" (Richardson-Richardson 1988) |
| 54 | AA8 | Hydropathy scale based on self-information values in the two-state model (50% |
| 55 | AA3 | PSSM-S |
| 56 | AA3 | Localized electrical effect (Fauchere et al. 1988) |
| 57 | AA11 | Hydrophobicity index (Argos et al. 1982) |
| 58 | AA5 | Normalized relative frequency of coil (Isogai et al. 1980) |
| 59 | AA2 | Average relative fractional occurrence in E0(i-1) (Rackovsky-Scheraga 1982) |
| 60 | AA8 | Interior composition of amino acids in nuclear proteins (percent) |
| 61 | AA5 | Normalized composition of membrane proteins (Nakashima et al. 1990) |
| 62 | AA3 | PSSM-Q |
| 63 | AA11 | Frequency of the 4th residue in turn (Chou-Fasman 1978b) |
| 64 | AA9 | PSSM-K |
| 65 | AA4 | Information measure for C-terminal helix (Robson-Suzuki 1976) |
| 66 | AA8 | Weights for beta-sheet at the window position of -5 (Qian-Sejnowski 1988) |
| 67 | AA5 | Principal property value z3 (Wold et al. 1987) |
| 68 | AA2 | Normalized frequency of beta-sheet from CF (Palau et al. 1981) |
| 69 | AA11 | Average relative probability of beta-sheet (Kanehisa-Tsong 1980) |
| 70 | AA4 | PSSM-I |
| 71 | AA7 | PSSM-R |
| 72 | AA3 | Hydrophobicity (Zimmerman et al. 1968) |
| 73 | AA8 | Normalized frequency of left-handed alpha-helix (Maxfield-Scheraga 1976) |
| 74 | AA11 | Zimm-Bragg parameter sigma x 1.0E4 (Sueki et al. 1984) |
| 75 | AA5 | Weights for alpha-helix at the window position of -5 (Qian-Sejnowski 1988) |
| 76 | AA5 | Weights for beta-sheet at the window position of -6 (Qian-Sejnowski 1988) |
| 77 | AA1 | Principal property value z3 (Wold et al. 1987) |
| 78 | AA8 | Side chain hydropathy corrected for solvation (Roseman 1988) |
| 79 | AA3 | PSSM-A |
| 80 | AA2 | Beta-strand indices for beta-proteins (Geisow-Roberts 1980) |
| 81 | AA11 | Information measure for pleated-sheet (Robson-Suzuki 1976) |
| 82 | AA6 | Short and medium range non-bonded energy per atom (Oobatake-Ooi 1977) |
| 83 | AA13 | PSSM-K |
| 84 | AA9 | PSSM-Q |
| 85 | AA8 | Average relative fractional occurrence in AL(i-1) (Rackovsky-Scheraga 1982) |
| 86 | AA2 | Average relative fractional occurrence in ER(i) (Rackovsky-Scheraga 1982) |
| 87 | AA11 | Relative preference value at C3 (Richardson-Richardson 1988) |
| 88 | AA11 | Normalized relative frequency of extended structure (Isogai et al. 1980) |
| 89 | AA5 | Ratio of average and computed composition (Nakashima et al. 1990) |
| 90 | AA10 | Weights for alpha-helix at the window position of -5 (Qian-Sejnowski 1988) |
| 91 | AA8 | Principal property value z3 (Wold et al. 1987) |
| 92 | AA2 | Zimm-Bragg parameter sigma x 1.0E4 (Sueki et al. 1984) |
| 93 | AA5 | Normalized frequency of turn in alpha+beta class (Palau et al. 1981) |
| 94 | AA11 | Side-chain contribution to protein stability (kJ/mol) (Takano-Yutani 2001) |
| 95 | AA9 | PSSM-P |
| 96 | AA2 | PSSM-P |
| 97 | AA8 | Hydrophobicity (Jones 1975) |
| 98 | AA5 | Average relative fractional occurrence in EL(i) (Rackovsky-Scheraga 1982) |
| 99 | AA11 | Free energy change of epsilon(i) to epsilon(ex) (Wertz-Scheraga 1978) |
| 100 | AA9 | PSSM-A |
| 101 | AA8 | Normalized frequency of chain reversal S (Tanaka-Scheraga 1977) |
| 102 | AA10 | Amphiphilicity index (Mitaku et al. 2002) |
| 103 | AA2 | Normalized frequency of extended structure (Maxfield-Scheraga 1976) |
| 104 | AA11 | Side chain orientational preference (Rackovsky-Scheraga 1977) |
| 105 | AA5 | Weights for beta-sheet at the window position of -2 (Qian-Sejnowski 1988) |
| 106 | AA6 | PSSM-E |
| 107 | AA2 | Weights for coil at the window position of -1 (Qian-Sejnowski 1988) |
| 108 | AA8 | Normalized frequency of extended structure (Burgess et al. 1974) |
| 109 | AA3 | Side-chain contribution to protein stability (kJ/mol) (Takano-Yutani 2001) |
| 110 | AA4 | Weights for coil at the window position of -6 (Qian-Sejnowski 1988) |
| 111 | AA11 | Beta-strand indices for alpha/beta-proteins (Geisow-Roberts 1980) |
| 112 | AA5 | Electron-ion interaction potential (Veljkovic et al. 1985) |
| 113 | AA9 | PSSM-D |
| 114 | AA10 | Transmembrane regions of non-mt-proteins (Nakashima et al. 1990) |
| 115 | AA8 | Weights for alpha-helix at the window position of -1 (Qian-Sejnowski 1988) |
| 116 | AA1 | PSSM-F |
| 117 | AA8 | A parameter of charge transfer capability (Charton-Charton 1983) |
| 118 | AA3 | Normalized positional residue frequency at helix termini N" (Aurora-Rose |
| 119 | AA8 | Ratio of buried and accessible molar fractions (Janin 1979) |
| 120 | AA11 | Partition coefficient (Pliska et al. 1981) |
| 121 | AA2 | Solvation free energy (Eisenberg-McLachlan 1986) |
| 122 | AA5 | Relative population of conformational state C (Vasquez et al. 1983) |
| 123 | AA3 | PSSM-N |
| 124 | AA11 | N.m.r. chemical shift of alpha-carbon (Fauchere et al. 1988) |
| 125 | AA12 | PSSM-G |
| 126 | AA8 | Weights for alpha-helix at the window position of 6 (Qian-Sejnowski 1988) |
| 127 | AA3 | Frequency of the 1st residue in turn (Chou-Fasman 1978b) |
| 128 | AA11 | Side chain hydropathy corrected for solvation (Roseman 1988) |
| 129 | AA8 | Normalized frequency of turn in alpha+beta class (Palau et al. 1981) |
| 130 | AA2 | Normalized frequency of chain reversal S (Tanaka-Scheraga 1977) |
| 131 | AA3 | PSSM-P |
| 132 | AA10 | Relative preference value at N2 (Richardson-Richardson 1988) |
| 133 | AA5 | Percentage of exposed residues (Janin et al. 1978) |
| 134 | AA5 | Frequency of the 1st residue in turn (Chou-Fasman 1978b) |
| 135 | AA11 | Flexibility parameter for no rigid neighbors (Karplus-Schulz 1985) |
| 136 | AA8 | Linker propensity from small dataset (linker length is less than six |
| 137 | AA8 | Linker propensity index (Suyama-Ohara 2003) |
| 138 | AA2 | Smoothed upsilon steric parameter (Fauchere et al. 1988) |
| 139 | AA11 | Hydrophobicity factor (Goldsack-Chalifoux 1973) |
| 140 | AA11 | Aperiodic indices for alpha-proteins (Geisow-Roberts 1980) |
| 141 | AA5 | A parameter of charge transfer capability (Charton-Charton 1983) |
| 142 | AA4 | Entire chain composition of amino acids in intracellular proteins of |
| 143 | AA8 | Normalized frequency of chain reversal D (Tanaka-Scheraga 1977) |
| 144 | AA11 | Normalized frequency of beta-sheet in alpha/beta class (Palau et al. 1981) |
| 145 | AA2 | Relative frequency in beta-sheet (Prabhakaran 1990) |
| 146 | AA6 | PSSM-P |
| 147 | AA10 | A parameter of charge transfer donor capability (Charton-Charton 1983) |
| 148 | AA5 | PSSM-P |
| 149 | AA5 | Helix initiation parameter at posision i-1 (Finkelstein et al. 1991) |
| 150 | AA11 | Steric parameter (Charton 1981) |
| 151 | AA12 | PSSM-P |
| 152 | AA8 | Hydrophobicity factor (Goldsack-Chalifoux 1973) |
| 153 | AA2 | Apparent partial specific volume (Bull-Breese 1974) |
| 154 | AA11 | Bulkiness (Zimmerman et al. 1968) |
| 155 | AA9 | PSSM-L |
| 156 | AA8 | Normalized relative frequency of coil (Isogai et al. 1980) |
| 157 | AA8 | Normalized frequency of beta-sheet from LG (Palau et al. 1981) |
| 158 | AA5 | Electron-ion interaction potential values (Cosic 1994) |
| 159 | AA2 | Normalized relative frequency of bend S (Isogai et al. 1980) |
| 160 | AA1 | Information measure for N-terminal helix (Robson-Suzuki 1976) |
| 161 | AA5 | Hydropathy scale based on self-information values in the two-state model (50% |
| 162 | AA11 | Normalized frequency of beta-sheet from CF (Palau et al. 1981) |
| 163 | AA3 | PSSM-K |
| 164 | AA2 | Hydropathy scale based on self-information values in the two-state model (50% |
| 165 | AA8 | Side chain torsion angle phi(AAAR) (Levitt 1976) |
| 166 | AA8 | Normalized relative frequency of bend S (Isogai et al. 1980) |
| 167 | AA11 | Linker propensity from small dataset (linker length is less than six |
| 168 | AA10 | Normalized frequency of C-terminal non helical region (Chou-Fasman 1978b) |
| 169 | AA5 | Alpha-helix indices for beta-proteins (Geisow-Roberts 1980) |
| 170 | AA11 | Weights for coil at the window position of -3 (Qian-Sejnowski 1988) |
| 171 | AA9 | PSSM-N |
| 172 | AA8 | Zimm-Bragg parameter sigma x 1.0E4 (Sueki et al. 1984) |
| 173 | AA4 | Normalized frequency of C-terminal helix (Chou-Fasman 1978b) |
| 174 | AA11 | Average reduced distance for C-alpha (Rackovsky-Scheraga 1977) |
| 175 | AA2 | Normalized frequency of beta-sheet with weights (Levitt 1978) |
| 176 | AA9 | PSSM-V |
| 177 | AA8 | Conformational parameter of beta-structure (Beghin-Dirkx 1975) |
| 178 | AA10 | PSSM-V |
| 179 | AA3 | Normalized frequency of N-terminal non helical region (Chou-Fasman 1978b) |
| 180 | AA11 | Hydrostatic pressure asymmetry index PAI (Di Giulio 2005) |
| 181 | AA6 | PSSM-Q |
| 182 | AA8 | Percentage of buried residues (Janin et al. 1978) |
| 183 | AA13 | PSSM-A |
| 184 | AA2 | Weights for alpha-helix at the window position of -1 (Qian-Sejnowski 1988) |
| 185 | AA9 | PSSM-R |
| 186 | AA11 | Retention coefficient in TFA (Browne et al. 1982) |
| 187 | AA2 | Beta-strand indices for alpha/beta-proteins (Geisow-Roberts 1980) |
| 188 | AA11 | Normalized frequency of beta-sheet unweighted (Levitt 1978) |
| 189 | AA5 | Composition of amino acids in nuclear proteins (percent) (Cedano et al. |
| 190 | AA12 | Hydrophobic parameter pi (Fauchere-Pliska 1983) |
| 191 | AA8 | Normalized frequency of extended structure (Tanaka-Scheraga 1977) |
| 192 | AA11 | Normalized frequency of turn in alpha+beta class (Palau et al. 1981) |
| 193 | AA11 | Transfer free energy (Simon 1976) Cited by Charton-Charton (1982) |
| 194 | AA2 | Aperiodic indices for alpha-proteins (Geisow-Roberts 1980) |
| 195 | AA3 | Frequency of occurrence in beta-bends (Lewis et al. 1971) |
| 196 | AA5 | Helix termination parameter at posision j+1 (Finkelstein et al. 1991) |
| 197 | AA8 | Alpha-helix propensity derived from designed sequences (Koehl-Levitt 1999) |
| 198 | AA12 | PSSM-A |
| 199 | AA8 | Hydrophobicity coefficient in RP-HPLC C8 with 0.1%TFA/MeCN/H2O (Wilce et al. |
| 200 | AA2 | 8 A contact number (Nishikawa-Ooi 1980) |
| 201 | AA11 | Normalized frequency of turn in alpha/beta class (Palau et al. 1981) |
| 202 | AA10 | Intercept in regression analysis (Prabhakaran-Ponnuswamy 1982) |
| 203 | AA6 | Intercept in regression analysis (Prabhakaran-Ponnuswamy 1982) |
| 204 | AA11 | Normalized relative frequency of double bend (Isogai et al. 1980) |
| 205 | AA8 | Transfer free energy (Simon 1976) Cited by Charton-Charton (1982) |
| 206 | AA5 | Hydration free energy (Robson-Osguthorpe 1979) |
| 207 | AA3 | Transfer free energy (Simon 1976) Cited by Charton-Charton (1982) |
| 208 | AA2 | Frequency of the 3rd residue in turn (Chou-Fasman 1978b) |
| 209 | AA11 | Weights for coil at the window position of -2 (Qian-Sejnowski 1988) |
| 210 | AA8 | Normalized frequency of beta-sheet in alpha+beta class (Palau et al. 1981) |
| 211 | AA5 | Melting point (Fasman 1976) |
| 212 | AA8 | Weights for beta-sheet at the window position of 4 (Qian-Sejnowski 1988) |
| 213 | AA1 | Retention coefficient in NaClO4 (Meek-Rossetti 1981) |
| 214 | AA8 | Average relative probability of inner beta-sheet (Kanehisa-Tsong 1980) |
| 215 | AA11 | Normalized frequency of turn from LG (Palau et al. 1981) |
| 216 | AA2 | Normalized frequency of extended structure (Tanaka-Scheraga 1977) |
| 217 | AA5 | Weights for coil at the window position of 5 (Qian-Sejnowski 1988) |
| 218 | AA8 | Correlation coefficient in regression analysis (Prabhakaran-Ponnuswamy 1982) |
| 219 | AA11 | Normalized hydrophobicity scales for alpha/beta-proteins (Cid et al. 1992) |
| 220 | AA4 | Electron-ion interaction potential values (Cosic 1994) |
| 221 | AA5 | Information measure for N-terminal helix (Robson-Suzuki 1976) |
| 222 | AA10 | Entire chain composition of amino acids in intracellular proteins of |
| 223 | AA3 | PSSM-H |
| 224 | AA8 | Normalized relative frequency of extended structure (Isogai et al. 1980) |
| 225 | AA8 | Weights for coil at the window position of -3 (Qian-Sejnowski 1988) |
| 226 | AA11 | Normalized frequency of extended structure (Tanaka-Scheraga 1977) |
| 227 | AA2 | Weights for beta-sheet at the window position of -1 (Qian-Sejnowski 1988) |
| 228 | AA7 | PSSM-D |
| 229 | AA2 | Relative preference value at N1 (Richardson-Richardson 1988) |
| 230 | AA8 | Ratio of average and computed composition (Nakashima et al. 1990) |
| 231 | AA5 | Relative preference value at N-cap (Richardson-Richardson 1988) |
| 232 | AA11 | Smoothed upsilon steric parameter (Fauchere et al. 1988) |
| 233 | AA11 | PSSM-P |
| 234 | AA10 | Net charge (Klein et al. 1984) |
| 235 | AA8 | Frequency of the 3rd residue in turn (Chou-Fasman 1978b) |
| 236 | AA11 | Beta-strand indices for beta-proteins (Geisow-Roberts 1980) |
| 237 | AA8 | The number of atoms in the side chain labelled 2+1 (Charton-Charton 1983) |
| 238 | AA3 | Beta-sheet propensity derived from designed sequences (Koehl-Levitt 1999) |
| 239 | AA11 | Average relative fractional occurrence in AL(i) (Rackovsky-Scheraga 1982) |
| 240 | AA2 | The number of atoms in the side chain labelled 1+1 (Charton-Charton 1983) |
| 241 | AA6 | PSSM-T |
| 242 | AA9 | Weights for alpha-helix at the window position of -4 (Qian-Sejnowski 1988) |
| 243 | AA5 | N.m.r. chemical shift of alpha-carbon (Fauchere et al. 1988) |
| 244 | AA8 | Average membrane preference: AMP07 (Degli Esposti et al. 1990) |
| 245 | AA8 | Relative mutability (Jones et al. 1992) |
| 246 | AA11 | Information measure for C-terminal turn (Robson-Suzuki 1976) |
| 247 | AA2 | Normalized positional residue frequency at helix termini N3 (Aurora-Rose |
| 248 | AA5 | Average relative fractional occurrence in A0(i) (Rackovsky-Scheraga 1982) |
| 249 | AA11 | Propensity of amino acids within pi-helices (Fodje-Al-Karadaghi 2002) |
| 250 | AA10 | Frequency of occurrence in beta-bends (Lewis et al. 1971) |
| 251 | AA5 | pK (-COOH) (Jones 1975) |
| 252 | AA8 | Average relative fractional occurrence in ER(i) (Rackovsky-Scheraga 1982) |
| 253 | AA12 | PSSM-Q |
| 254 | AA2 | Hydrophobicity index (Argos et al. 1982) |
| 255 | AA8 | Normalized frequency of extended structure (Maxfield-Scheraga 1976) |
| 256 | AA5 | Number of full nonbonding orbitals (Fauchere et al. 1988) |
| 257 | AA5 | Relative preference value at N" (Richardson-Richardson 1988) |
| 258 | AA8 | Average relative fractional occurrence in A0(i) (Rackovsky-Scheraga 1982) |
| 259 | AA11 | Normalized hydrophobicity scales for beta-proteins (Cid et al. 1992) |
| 260 | AA6 | Hydrophobicity coefficient in RP-HPLC C4 with 0.1%TFA/MeCN/H2O (Wilce et al. |
| 261 | AA5 | Average relative fractional occurrence in AL(i) (Rackovsky-Scheraga 1982) |
| 262 | AA2 | Normalized frequency of beta-sheet (Chou-Fasman 1978b) |
| 263 | AA3 | Frequency of the 3rd residue in turn (Chou-Fasman 1978b) |
| 264 | AA11 | Hydrophobicity (Zimmerman et al. 1968) |
| 265 | AA4 | Information measure for middle turn (Robson-Suzuki 1976) |
| 266 | AA8 | Entire chain composition of amino acids in intracellular proteins of |
| 267 | AA11 | Normalized positional residue frequency at helix termini N"' (Aurora-Rose |
| 268 | AA8 | Steric parameter (Charton 1981) |
| 269 | AA5 | Normalized frequency of N-terminal helix (Chou-Fasman 1978b) |
| 270 | AA5 | Average relative fractional occurrence in AL(i-1) (Rackovsky-Scheraga 1982) |
| 271 | AA11 | Information measure for middle turn (Robson-Suzuki 1976) |
| 272 | AA3 | PSSM-T |
| 273 | AA8 | AA composition of mt-proteins from animal (Nakashima et al. 1990) |
| 274 | AA2 | Free energy in beta-strand region (Munoz-Serrano 1994) |
| 275 | AA10 | Composition (Grantham 1974) |
| 276 | AA11 | Normalized frequency of beta-sheet with weights (Levitt 1978) |
| 277 | AA8 | Helix initiation parameter at posision i-1 (Finkelstein et al. 1991) |
| 278 | AA8 | Weights for coil at the window position of 5 (Qian-Sejnowski 1988) |
| 279 | AA7 | PSSM-E |
| 280 | AA11 | Average surrounding hydrophobicity (Manavalan-Ponnuswamy 1978) |
| 281 | AA8 | Hydrophobicity (Zimmerman et al. 1968) |
| 282 | AA11 | Normalized positional residue frequency at helix termini Cc (Aurora-Rose |
| 283 | AA10 | Weights for coil at the window position of -4 (Qian-Sejnowski 1988) |
| 284 | AA5 | Relative preference value at C-cap (Richardson-Richardson 1988) |
| 285 | AA2 | Hydrophobicity factor (Goldsack-Chalifoux 1973) |
| 286 | AA6 | PSSM-A |
| 287 | AA8 | The Kerr-constant increments (Khanarian-Moore 1980) |
| 288 | AA2 | Information measure for extended without H-bond (Robson-Suzuki 1976) |
| 289 | AA8 | Graph shape index (Fauchere et al. 1988) |
| 290 | AA4 | Accessible surface area (Radzicka-Wolfenden 1988) |
| 291 | AA8 | PSSM-P |
| 292 | AA11 | Normalized frequency of beta-sheet (Chou-Fasman 1978b) |
| 293 | AA5 | Localized electrical effect (Fauchere et al. 1988) |
| 294 | AA2 | Weights for coil at the window position of -3 (Qian-Sejnowski 1988) |
| 295 | AA8 | Normalized frequency of alpha-helix (Burgess et al. 1974) |
| 296 | AA3 | Normalized frequency of chain reversal S (Tanaka-Scheraga 1977) |
| 297 | AA11 | Normalized relative frequency of bend R (Isogai et al. 1980) |
| 298 | AA2 | Average relative fractional occurrence in EL(i-1) (Rackovsky-Scheraga 1982) |
| 299 | AA10 | Radius of gyration of side chain (Levitt 1976) |
| 300 | AA6 | PSSM-V |
| 301 | AA8 | Hydrophobic parameter (Levitt 1976) |
| 302 | AA1 | Side-chain contribution to protein stability (kJ/mol) (Takano-Yutani 2001) |
| 303 | AA5 | Weights for alpha-helix at the window position of 3 (Qian-Sejnowski 1988) |
| 304 | AA11 | Retention coefficient in HFBA (Browne et al. 1982) |
| 305 | AA2 | Relative preference value at C3 (Richardson-Richardson 1988) |
| 306 | AA5 | Relative preference value at N' (Richardson-Richardson 1988) |
| 307 | AA8 | Electron-ion interaction potential values (Cosic 1994) |
| 308 | AA8 | Weights for beta-sheet at the window position of -2 (Qian-Sejnowski 1988) |
| 309 | AA5 | Side chain hydropathy uncorrected for solvation (Roseman 1988) |
| 310 | AA11 | Normalized frequency of beta-sheet (Crawford et al. 1973) |
| 311 | AA2 | Hydrophobicity (Jones 1975) |
| 312 | AA8 | Slope in regression analysis x 1.0E1 (Prabhakaran-Ponnuswamy 1982) |
| 313 | AA8 | Relative population of conformational state C (Vasquez et al. 1983) |
| 314 | AA11 | Frequency of the 3rd residue in turn (Chou-Fasman 1978b) |
| 315 | AA8 | Average relative fractional occurrence in ER(i-1) (Rackovsky-Scheraga 1982) |
| 316 | AA10 | Normalized frequency of alpha-helix (Tanaka-Scheraga 1977) |
| 317 | AA8 | Side chain angle theta(AAR) (Levitt 1976) |
| 318 | AA4 | Interior composition of amino acids in extracellular proteins of mesophiles |
| 319 | AA1 | PSSM-T |
| 320 | AA2 | Normalized frequency of left-handed helix (Tanaka-Scheraga 1977) |
| 321 | AA11 | Relative population of conformational state E (Vasquez et al. 1983) |
| 322 | AA3 | Normalized relative frequency of bend S (Isogai et al. 1980) |
| 323 | AA11 | Average reduced distance for side chain (Meirovitch et al. 1980) |
| 324 | AA8 | Weights for alpha-helix at the window position of 2 (Qian-Sejnowski 1988) |
| 325 | AA5 | Interior composition of amino acids in extracellular proteins of mesophiles |
| 326 | AA2 | Normalized frequency of zeta R (Tanaka-Scheraga 1977) |
| 327 | AA2 | Weights for beta-sheet at the window position of -2 (Qian-Sejnowski 1988) |
| 328 | AA12 | Transfer free energy to lipophilic phase (von Heijne-Blomberg 1979) |
| 329 | AA9 | Relative preference value at N3 (Richardson-Richardson 1988) |
| 330 | AA11 | Relative frequency in beta-sheet (Prabhakaran 1990) |
| 331 | AA8 | Alpha-helix indices for beta-proteins (Geisow-Roberts 1980) |
| 332 | AA10 | Isoelectric point (Zimmerman et al. 1968) |
| 333 | AA11 | Normalized flexibility parameters (B-values) for each residue surrounded by |
| 334 | AA8 | AA composition of MEM of single-spanning proteins (Nakashima-Nishikawa 1992) |
| 335 | AA6 | PSSM-G |
| 336 | AA2 | Transfer free energy (Simon 1976) Cited by Charton-Charton (1982) |
| 337 | AA8 | N.m.r. chemical shift of alpha-carbon (Fauchere et al. 1988) |
| 338 | AA4 | Weights for alpha-helix at the window position of 3 (Qian-Sejnowski 1988) |
| 339 | AA3 | PSSM-R |
| 340 | AA8 | Normalized frequency of C-terminal non beta region (Chou-Fasman 1978b) |
| 341 | AA11 | Interactivity scale obtained by maximizing the mean of correlation |
| 342 | AA5 | pK-C (Fasman 1976) |
| 343 | AA2 | Normalized positional residue frequency at helix termini N'(Aurora-Rose |
| 344 | AA5 | Relative preference value at C2 (Richardson-Richardson 1988) |
| 345 | AA8 | Normalized frequency of isolated helix (Tanaka-Scheraga 1977) |
| 346 | AA11 | Conformational preference for parallel beta-strands (Lifson-Sander 1979) |
| 347 | AA8 | Beta-strand indices for beta-proteins (Geisow-Roberts 1980) |
| 348 | AA2 | Normalized frequency of N-terminal beta-sheet (Chou-Fasman 1978b) |
| 349 | AA5 | Weights for alpha-helix at the window position of -1 (Qian-Sejnowski 1988) |
| 350 | AA6 | Side chain interaction parameter (Krigbaum-Rubin 1971) |
| 351 | AA2 | Helix-coil equilibrium constant (Ptitsyn-Finkelstein 1983) |
| 352 | AA8 | Alpha-helix indices for alpha-proteins (Geisow-Roberts 1980) |
| 353 | AA5 | Normalized relative frequency of extended structure (Isogai et al. 1980) |
| 354 | AA8 | Normalized positional residue frequency at helix termini N"' (Aurora-Rose |
| 355 | AA2 | N.m.r. chemical shift of alpha-carbon (Fauchere et al. 1988) |
| 356 | AA5 | Normalized positional residue frequency at helix termini N'(Aurora-Rose |
| 357 | AA11 | Aperiodic indices for alpha/beta-proteins (Geisow-Roberts 1980) |
| 358 | AA2 | Normalized frequency of turn (Crawford et al. 1973) |
| 359 | AA8 | Surrounding hydrophobicity in alpha-helix (Ponnuswamy et al. 1980) |
| 360 | AA9 | Normalized frequency of beta-sheet (Crawford et al. 1973) |
| 361 | AA8 | Relative mutability (Dayhoff et al. 1978a) |
| 362 | AA8 | Normalized frequency of beta-sheet (Crawford et al. 1973) |
| 363 | AA11 | Retention coefficient in HPLC pH7.4 (Meek 1980) |
| 364 | AA6 | PSSM-K |
| 365 | AA5 | Normalized frequency of turn in all-alpha class (Palau et al. 1981) |
| 366 | AA10 | Optimized transfer energy parameter (Oobatake et al. 1985) |
| 367 | AA2 | Linker propensity from small dataset (linker length is less than six |
| 368 | AA11 | Flexibility parameter for one rigid neighbor (Karplus-Schulz 1985) |
| 369 | AA8 | Surrounding hydrophobicity in turn (Ponnuswamy et al. 1980) |
| 370 | AA11 | Conformational parameter of inner helix (Beghin-Dirkx 1975) |
| 371 | AA4 | Information measure for turn (Robson-Suzuki 1976) |
| 372 | AA5 | Normalized positional residue frequency at helix termini C' (Aurora-Rose |
| 373 | AA3 | Hydrostatic pressure asymmetry index PAI (Di Giulio 2005) |
| 374 | AA2 | Free energy change of epsilon(i) to epsilon(ex) (Wertz-Scheraga 1978) |
| 375 | AA12 | PSSM-H |
| 376 | AA11 | Side chain interaction parameter (Krigbaum-Rubin 1971) |
| 377 | AA7 | PSSM-T |
| 378 | AA8 | Relative preference value at C-cap (Richardson-Richardson 1988) |
| 379 | AA2 | STERIMOL minimum width of the side chain (Fauchere et al. 1988) |
| 380 | AA6 | Radius of gyration of side chain (Levitt 1976) |
| 381 | AA8 | Transmembrane regions of non-mt-proteins (Nakashima et al. 1990) |
| 382 | AA4 | Weights for coil at the window position of -4 (Qian-Sejnowski 1988) |
| 383 | AA11 | Transfer free energy CHP/water (Lawson et al. 1984) |
| 384 | AA2 | Hydrophobicity (Zimmerman et al. 1968) |
| 385 | AA10 | PSSM-K |
| 386 | AA5 | Weights for beta-sheet at the window position of -5 (Qian-Sejnowski 1988) |
| 387 | AA3 | Normalized frequency of turn in all-alpha class (Palau et al. 1981) |
| 388 | AA1 | AA composition of EXT of single-spanning proteins (Nakashima-Nishikawa 1992) |
| 389 | AA11 | Optical rotation (Fasman 1976) |
| 390 | AA8 | Relative preference value at C3 (Richardson-Richardson 1988) |
| 391 | AA11 | Alpha-helix indices for alpha-proteins (Geisow-Roberts 1980) |
| 392 | AA11 | Normalized frequency of beta-sheet in all-beta class (Palau et al. 1981) |
| 393 | AA8 | Linker propensity from long dataset (linker length is greater than 14 |
| 394 | AA2 | Average membrane preference: AMP07 (Degli Esposti et al. 1990) |
| 395 | AA10 | Information measure for middle turn (Robson-Suzuki 1976) |
| 396 | AA8 | Hydrophobicity scales (Ponnuswamy 1993) |
| 397 | AA8 | Electron-ion interaction potential (Veljkovic et al. 1985) |
| 398 | AA5 | Value of theta(i-1) (Rackovsky-Scheraga 1982) |
| 399 | AA11 | Mean fractional area loss (Rose et al. 1985) |
| 400 | AA2 | Relative preference value at N" (Richardson-Richardson 1988) |
| 401 | AA11 | Linker propensity from 3-linker dataset (George-Heringa 2003) |
| 402 | AA8 | STERIMOL minimum width of the side chain (Fauchere et al. 1988) |
| 403 | AA7 | PSSM-L |
| 404 | AA5 | Hydrophilicity scale (Kuhn et al. 1995) |
| 405 | AA5 | Helix termination parameter at posision j-2j-1j (Finkelstein et al. 1991) |
| 406 | AA12 | PSSM-D |
| 407 | AA8 | Apparent partial specific volume (Bull-Breese 1974) |
| 408 | AA5 | Relative mutability (Jones et al. 1992) |
| 409 | AA11 | The Chou-Fasman parameter of the coil conformation (Charton-Charton 1983) |
| 410 | AA2 | Normalized frequency of beta-sheet in all-beta class (Palau et al. 1981) |
| 411 | AA13 | PSSM-E |
| 412 | AA8 | Information measure for alpha-helix (Robson-Suzuki 1976) |
| 413 | AA11 | Retention coefficient in HPLC pH2.1 (Meek 1980) |
| 414 | AA10 | Atom-based hydrophobic moment (Eisenberg-McLachlan 1986) |
| 415 | AA11 | Surrounding hydrophobicity in beta-sheet (Ponnuswamy et al. 1980) |
| 416 | AA2 | RF rank (Zimmerman et al. 1968) |
| 417 | AA8 | Average gain ratio in surrounding hydrophobicity (Ponnuswamy et al. 1980) |
| 418 | AA2 | Weights for alpha-helix at the window position of 0 (Qian-Sejnowski 1988) |
| 419 | AA4 | Normalized positional residue frequency at helix termini Cc (Aurora-Rose |
| 420 | AA8 | The number of atoms in the side chain labelled 1+1 (Charton-Charton 1983) |
| 421 | AA8 | Average relative fractional occurrence in AR(i) (Rackovsky-Scheraga 1982) |
| 422 | AA5 | Membrane preference for cytochrome b: MPH89 (Degli Esposti et al. 1990) |
| 423 | AA11 | Relative preference value at N1 (Richardson-Richardson 1988) |
| 424 | AA10 | PSSM-A |
| 425 | AA11 | Normalized average hydrophobicity scales (Cid et al. 1992) |
| 426 | AA8 | Weights for beta-sheet at the window position of 6 (Qian-Sejnowski 1988) |
| 427 | AA6 | PSSM-M |
| 428 | AA8 | Free energy change of epsilon(i) to epsilon(ex) (Wertz-Scheraga 1978) |
| 429 | AA2 | Hydrophilicity value (Hopp-Woods 1981) |
| 430 | AA4 | Normalized frequency of alpha-helix (Tanaka-Scheraga 1977) |
| 431 | AA10 | Normalized frequency of alpha-helix in alpha+beta class (Palau et al. 1981) |
| 432 | AA12 | Side chain hydropathy uncorrected for solvation (Roseman 1988) |
| 433 | AA2 | Normalized positional residue frequency at helix termini C3 (Aurora-Rose |
| 434 | AA8 | Transfer free energy from chx to wat (Radzicka-Wolfenden 1988) |
| 435 | AA11 | Normalized frequency of beta-turn (Chou-Fasman 1978a) |
| 436 | AA11 | Normalized frequency of extended structure (Maxfield-Scheraga 1976) |
| 437 | AA8 | Weights for alpha-helix at the window position of 4 (Qian-Sejnowski 1988) |
| 438 | AA5 | Frequency of occurrence in beta-bends (Lewis et al. 1971) |
| 439 | AA9 | PSSM-S |
| 440 | AA4 | PSSM-V |
| 441 | AA4 | PSSM-K |
| 442 | AA3 | Hydrophobicity factor (Goldsack-Chalifoux 1973) |
| 443 | AA2 | Hydrostatic pressure asymmetry index PAI (Di Giulio 2005) |
| 444 | AA8 | Normalized positional residue frequency at helix termini N4'(Aurora-Rose |
| 445 | AA5 | Relative preference value at C' (Richardson-Richardson 1988) |
| 446 | AA11 | Information value for accessibility; average fraction 23% (Biou et al. 1988) |
| 447 | AA8 | STERIMOL maximum width of the side chain (Fauchere et al. 1988) |
| 448 | AA2 | Information measure for C-terminal turn (Robson-Suzuki 1976) |
| 449 | AA9 | Average non-bonded energy per atom (Oobatake-Ooi 1977) |
| 450 | AA11 | Conformational preference for antiparallel beta-strands (Lifson-Sander 1979) |
| 451 | AA2 | Principal property value z3 (Wold et al. 1987) |
| 452 | AA8 | Weights for beta-sheet at the window position of -1 (Qian-Sejnowski 1988) |
| 453 | AA4 | Average relative fractional occurrence in E0(i-1) (Rackovsky-Scheraga 1982) |
| 454 | AA5 | Normalized frequency of left-handed alpha-helix (Maxfield-Scheraga 1976) |
| 455 | AA11 | PSSM-C |
| 456 | AA1 | PSSM-A |
| 457 | AA11 | Entire chain composition of amino acids in intracellular proteins of |
| 458 | AA10 | Normalized frequency of turn from CF (Palau et al. 1981) |
| 459 | AA8 | Information value for accessibility; average fraction 35% (Biou et al. 1988) |
| 460 | AA2 | Optimized beta-structure-coil equilibrium constant (Oobatake et al. 1985) |
| 461 | AA5 | Normalized frequency of beta-sheet with weights (Levitt 1978) |
| 462 | AA11 | Interactivity scale obtained by maximizing the mean of correlation |
| 463 | AA8 | Normalized frequency of alpha-helix in all-alpha class (Palau et al. 1981) |
| 464 | AA2 | Relative preference value at N' (Richardson-Richardson 1988) |
| 465 | AA2 | Information measure for N-terminal helix (Robson-Suzuki 1976) |
| 466 | AA5 | Weights for alpha-helix at the window position of -6 (Qian-Sejnowski 1988) |
| 467 | AA8 | Free energy change of alpha(Ri) to alpha(Rh) (Wertz-Scheraga 1978) |
| 468 | AA11 | Hydropathy scale based on self-information values in the two-state model (36% |
| 469 | AA2 | Weights for beta-sheet at the window position of 2 (Qian-Sejnowski 1988) |
| 470 | AA12 | PSSM-K |
| 471 | AA1 | Relative population of conformational state A (Vasquez et al. 1983) |
| 472 | AA8 | Optimized beta-structure-coil equilibrium constant (Oobatake et al. 1985) |
| 473 | AA11 | Normalized frequency of turn from CF (Palau et al. 1981) |
| 474 | AA6 | Optimized side chain interaction parameter (Oobatake et al. 1985) |
| 475 | AA8 | Weights for alpha-helix at the window position of 0 (Qian-Sejnowski 1988) |
| 476 | AA6 | PSSM-R |
| 477 | AA8 | Principal component I (Sneath 1966) |
| 478 | AA2 | Average relative fractional occurrence in AL(i) (Rackovsky-Scheraga 1982) |
| 479 | AA3 | Relative mutability (Dayhoff et al. 1978a) |
| 480 | AA11 | Normalized hydrophobicity scales for alpha-proteins (Cid et al. 1992) |
| 481 | AA2 | Bulkiness (Zimmerman et al. 1968) |
| 482 | AA8 | Direction of hydrophobic moment (Eisenberg-McLachlan 1986) |
| 483 | AA5 | Information measure for C-terminal turn (Robson-Suzuki 1976) |
| 484 | AA4 | Information measure for loop (Robson-Suzuki 1976) |
| 485 | AA11 | Loss of Side chain hydropathy by helix formation (Roseman 1988) |
| 486 | AA5 | Normalized positional residue frequency at helix termini N2 (Aurora-Rose |
| 487 | AA8 | Weights for coil at the window position of -6 (Qian-Sejnowski 1988) |
| 488 | AA11 | Composition of amino acids in anchored proteins (percent) (Cedano et al. |
| 489 | AA12 | PSSM-R |
| 490 | AA10 | Interior composition of amino acids in extracellular proteins of mesophiles |
| 491 | AA5 | Turn propensity scale for transmembrane helices (Monne et al. 1999) |
| 492 | AA2 | Information measure for pleated-sheet (Robson-Suzuki 1976) |
| 493 | AA11 | Spin-spin coupling constants 3JHalpha-NH (Bundi-Wuthrich 1979) |
| 494 | AA8 | Normalized positional residue frequency at helix termini Nc (Aurora-Rose |
| 495 | AA10 | Normalized positional residue frequency at helix termini N2 (Aurora-Rose |
| 496 | AA11 | Optimal matching hydrophobicity (Sweet-Eisenberg 1983) |
| 497 | AA6 | STERIMOL length of the side chain (Fauchere et al. 1988) |
| 498 | AA8 | Hydropathy scale based on self-information values in the two-state model (20% |
| 499 | AA5 | Relative frequency in beta-sheet (Prabhakaran 1990) |
| 500 | AA2 | Weights for beta-sheet at the window position of 4 (Qian-Sejnowski 1988) |
