# Supplementary material for: Prediction and Analysis of Protein Hydroxyproline and Hydroxylysine
Source: PLoS One. 2010 Dec 31;5(12):e15917. doi: 10.1371/journal.pone.0015917 (PMC3013141; doi:10.1371/journal.pone.0015917)
Supplement: Table S5 — Performance of 500 NNA predictors for hydroxyproline dataset. (DOC) [file pone.0015917.s005.doc]

**Table S5. Performance of 500 NNA predictors for hydroxyproline dataset**

Listed below are the Sensitivity (Sn), Specificity (Sp), Accuracy (AC) and Mathew correlation coefficient (MCC) of 500 predictors for hydroxyproline dataset constructed by nearest neighbor algorithm and evaluated by Jackknife test.

| Index | Sn | Sp | AC | MCC |
| --- | --- | --- | --- | --- |
| 1 | 1 | 0 | 0.3333 | - |
| 2 | 0.9499 | 0.2699 | 0.4966 | 0.2606 |
| 3 | 0.91 | 0.4137 | 0.5792 | 0.3312 |
| 4 | 0.8333 | 0.5686 | 0.6568 | 0.3822 |
| 5 | 0.7611 | 0.6209 | 0.6677 | 0.3602 |
| 6 | 0.7655 | 0.6239 | 0.6711 | 0.3671 |
| 7 | 0.6534 | 0.7205 | 0.6981 | 0.3592 |
| 8 | 0.6475 | 0.722 | 0.6971 | 0.3553 |
| 9 | 0.59 | 0.7559 | 0.7006 | 0.3398 |
| 10 | 0.5973 | 0.7581 | 0.7045 | 0.349 |
| 11 | 0.5737 | 0.7795 | 0.7109 | 0.352 |
| 12 | 0.5796 | 0.7802 | 0.7134 | 0.3582 |
| 13 | 0.5914 | 0.7758 | 0.7144 | 0.3638 |
| 14 | 0.5914 | 0.778 | 0.7158 | 0.3664 |
| 15 | 0.5841 | 0.7729 | 0.7099 | 0.3537 |
| 16 | 0.5811 | 0.7847 | 0.7168 | 0.3647 |
| 17 | 0.5796 | 0.7898 | 0.7198 | 0.3695 |
| 18 | 0.5664 | 0.792 | 0.7168 | 0.36 |
| 19 | 0.5693 | 0.7802 | 0.7099 | 0.3488 |
| 20 | 0.5944 | 0.7832 | 0.7203 | 0.375 |
| 21 | 0.6077 | 0.7847 | 0.7257 | 0.3888 |
| 22 | 0.6136 | 0.7898 | 0.7311 | 0.4001 |
| 23 | 0.615 | 0.7913 | 0.7325 | 0.4032 |
| 24 | 0.6106 | 0.7972 | 0.735 | 0.4062 |
| 25 | 0.6047 | 0.8009 | 0.7355 | 0.4053 |
| 26 | 0.5826 | 0.7965 | 0.7252 | 0.38 |
| 27 | 0.5855 | 0.7965 | 0.7262 | 0.3827 |
| 28 | 0.5944 | 0.7987 | 0.7306 | 0.3934 |
| 29 | 0.6032 | 0.8031 | 0.7365 | 0.4066 |
| 30 | 0.5885 | 0.8009 | 0.7301 | 0.3907 |
| 31 | 0.5944 | 0.8009 | 0.7321 | 0.396 |
| 32 | 0.6077 | 0.8001 | 0.736 | 0.4071 |
| 33 | 0.6062 | 0.7979 | 0.734 | 0.4031 |
| 34 | 0.6106 | 0.8105 | 0.7439 | 0.4222 |
| 35 | 0.6254 | 0.8097 | 0.7483 | 0.4345 |
| 36 | 0.6372 | 0.8119 | 0.7537 | 0.4477 |
| 37 | 0.6342 | 0.8134 | 0.7537 | 0.4468 |
| 38 | 0.6372 | 0.8031 | 0.7478 | 0.437 |
| 39 | 0.6283 | 0.7987 | 0.7419 | 0.4238 |
| 40 | 0.6254 | 0.7913 | 0.736 | 0.4125 |
| 41 | 0.6239 | 0.7942 | 0.7375 | 0.4146 |
| 42 | 0.6106 | 0.7935 | 0.7325 | 0.4018 |
| 43 | 0.6077 | 0.7898 | 0.7291 | 0.3948 |
| 44 | 0.615 | 0.7906 | 0.7321 | 0.4023 |
| 45 | 0.6209 | 0.7913 | 0.7345 | 0.4085 |
| 46 | 0.6209 | 0.7898 | 0.7335 | 0.4068 |
| 47 | 0.6165 | 0.7935 | 0.7345 | 0.4071 |
| 48 | 0.6165 | 0.7957 | 0.736 | 0.4097 |
| 49 | 0.6106 | 0.8031 | 0.7389 | 0.4133 |
| 50 | 0.6091 | 0.8038 | 0.7389 | 0.4128 |
| 51 | 0.6062 | 0.8068 | 0.7399 | 0.4137 |
| 52 | 0.6106 | 0.8016 | 0.738 | 0.4115 |
| 53 | 0.6106 | 0.7994 | 0.7365 | 0.4088 |
| 54 | 0.6062 | 0.8009 | 0.736 | 0.4066 |
| 55 | 0.6121 | 0.7994 | 0.737 | 0.4102 |
| 56 | 0.6239 | 0.8075 | 0.7463 | 0.4305 |
| 57 | 0.6268 | 0.8075 | 0.7473 | 0.4331 |
| 58 | 0.6313 | 0.8024 | 0.7453 | 0.4309 |
| 59 | 0.6327 | 0.8001 | 0.7443 | 0.4295 |
| 60 | 0.6298 | 0.7965 | 0.7409 | 0.4225 |
| 61 | 0.6342 | 0.7994 | 0.7443 | 0.43 |
| 62 | 0.6195 | 0.8024 | 0.7414 | 0.4203 |
| 63 | 0.6254 | 0.7994 | 0.7414 | 0.4221 |
| 64 | 0.6313 | 0.8024 | 0.7453 | 0.4309 |
| 65 | 0.6268 | 0.8038 | 0.7448 | 0.4287 |
| 66 | 0.6268 | 0.8097 | 0.7488 | 0.4358 |
| 67 | 0.6268 | 0.8068 | 0.7468 | 0.4322 |
| 68 | 0.6298 | 0.8031 | 0.7453 | 0.4304 |
| 69 | 0.6313 | 0.8024 | 0.7453 | 0.4309 |
| 70 | 0.6298 | 0.8068 | 0.7478 | 0.4348 |
| 71 | 0.649 | 0.8046 | 0.7527 | 0.4493 |
| 72 | 0.6504 | 0.8112 | 0.7576 | 0.4585 |
| 73 | 0.6475 | 0.8156 | 0.7596 | 0.4613 |
| 74 | 0.6445 | 0.8164 | 0.7591 | 0.4596 |
| 75 | 0.6401 | 0.8083 | 0.7522 | 0.4458 |
| 76 | 0.6416 | 0.809 | 0.7532 | 0.448 |
| 77 | 0.6386 | 0.8083 | 0.7517 | 0.4445 |
| 78 | 0.6401 | 0.806 | 0.7507 | 0.4432 |
| 79 | 0.6313 | 0.8068 | 0.7483 | 0.4362 |
| 80 | 0.6283 | 0.8083 | 0.7483 | 0.4353 |
| 81 | 0.6268 | 0.8068 | 0.7468 | 0.4322 |
| 82 | 0.6283 | 0.809 | 0.7488 | 0.4362 |
| 83 | 0.6342 | 0.8038 | 0.7473 | 0.4352 |
| 84 | 0.6386 | 0.8016 | 0.7473 | 0.4366 |
| 85 | 0.6386 | 0.7994 | 0.7458 | 0.4339 |
| 86 | 0.6386 | 0.8009 | 0.7468 | 0.4357 |
| 87 | 0.6327 | 0.7979 | 0.7429 | 0.4269 |
| 88 | 0.6313 | 0.7972 | 0.7419 | 0.4247 |
| 89 | 0.6283 | 0.8016 | 0.7439 | 0.4273 |
| 90 | 0.6254 | 0.8024 | 0.7434 | 0.4256 |
| 91 | 0.6268 | 0.8046 | 0.7453 | 0.4296 |
| 92 | 0.6254 | 0.8031 | 0.7439 | 0.4265 |
| 93 | 0.6254 | 0.8046 | 0.7448 | 0.4282 |
| 94 | 0.6283 | 0.8024 | 0.7443 | 0.4282 |
| 95 | 0.6254 | 0.8009 | 0.7424 | 0.4238 |
| 96 | 0.6209 | 0.7972 | 0.7384 | 0.4155 |
| 97 | 0.6239 | 0.7942 | 0.7375 | 0.4146 |
| 98 | 0.618 | 0.7972 | 0.7375 | 0.4128 |
| 99 | 0.6165 | 0.7994 | 0.7384 | 0.4141 |
| 100 | 0.6136 | 0.8009 | 0.7384 | 0.4133 |
| 101 | 0.6165 | 0.7987 | 0.738 | 0.4133 |
| 102 | 0.618 | 0.795 | 0.736 | 0.4102 |
| 103 | 0.6209 | 0.7987 | 0.7394 | 0.4172 |
| 104 | 0.6224 | 0.792 | 0.7355 | 0.4107 |
| 105 | 0.6165 | 0.7906 | 0.7325 | 0.4036 |
| 106 | 0.6209 | 0.7898 | 0.7335 | 0.4068 |
| 107 | 0.6106 | 0.792 | 0.7316 | 0.4001 |
| 108 | 0.6165 | 0.7942 | 0.735 | 0.408 |
| 109 | 0.615 | 0.7935 | 0.734 | 0.4058 |
| 110 | 0.618 | 0.7972 | 0.7375 | 0.4128 |
| 111 | 0.615 | 0.7942 | 0.7345 | 0.4067 |
| 112 | 0.6091 | 0.792 | 0.7311 | 0.3988 |
| 113 | 0.6136 | 0.792 | 0.7325 | 0.4027 |
| 114 | 0.615 | 0.7928 | 0.7335 | 0.4049 |
| 115 | 0.6165 | 0.795 | 0.7355 | 0.4089 |
| 116 | 0.6106 | 0.7972 | 0.735 | 0.4062 |
| 117 | 0.6165 | 0.7987 | 0.738 | 0.4133 |
| 118 | 0.6121 | 0.7928 | 0.7325 | 0.4023 |
| 119 | 0.6136 | 0.7928 | 0.733 | 0.4036 |
| 120 | 0.6136 | 0.7906 | 0.7316 | 0.401 |
| 121 | 0.6106 | 0.7957 | 0.734 | 0.4044 |
| 122 | 0.6106 | 0.7972 | 0.735 | 0.4062 |
| 123 | 0.6032 | 0.7913 | 0.7286 | 0.3926 |
| 124 | 0.6047 | 0.7906 | 0.7286 | 0.393 |
| 125 | 0.5944 | 0.7965 | 0.7291 | 0.3907 |
| 126 | 0.6003 | 0.7898 | 0.7266 | 0.3882 |
| 127 | 0.5988 | 0.7913 | 0.7271 | 0.3886 |
| 128 | 0.5973 | 0.7906 | 0.7262 | 0.3864 |
| 129 | 0.5929 | 0.7898 | 0.7242 | 0.3815 |
| 130 | 0.5885 | 0.7972 | 0.7276 | 0.3863 |
| 131 | 0.5914 | 0.7979 | 0.7291 | 0.3898 |
| 132 | 0.5885 | 0.7957 | 0.7266 | 0.3845 |
| 133 | 0.5914 | 0.7957 | 0.7276 | 0.3872 |
| 134 | 0.5929 | 0.7979 | 0.7296 | 0.3911 |
| 135 | 0.5929 | 0.8016 | 0.7321 | 0.3956 |
| 136 | 0.5959 | 0.8038 | 0.7345 | 0.4009 |
| 137 | 0.5914 | 0.8009 | 0.7311 | 0.3934 |
| 138 | 0.59 | 0.8024 | 0.7316 | 0.3938 |
| 139 | 0.59 | 0.8046 | 0.733 | 0.3965 |
| 140 | 0.5914 | 0.8068 | 0.735 | 0.4005 |
| 141 | 0.5914 | 0.8075 | 0.7355 | 0.4014 |
| 142 | 0.5929 | 0.8075 | 0.736 | 0.4027 |
| 143 | 0.6003 | 0.8016 | 0.7345 | 0.4022 |
| 144 | 0.6003 | 0.8024 | 0.735 | 0.4031 |
| 145 | 0.5885 | 0.8053 | 0.733 | 0.3961 |
| 146 | 0.5841 | 0.7994 | 0.7276 | 0.3849 |
| 147 | 0.5826 | 0.7972 | 0.7257 | 0.3809 |
| 148 | 0.5796 | 0.795 | 0.7232 | 0.3756 |
| 149 | 0.5914 | 0.7979 | 0.7291 | 0.3898 |
| 150 | 0.5988 | 0.7979 | 0.7316 | 0.3965 |
| 151 | 0.6062 | 0.7979 | 0.734 | 0.4031 |
| 152 | 0.6032 | 0.7928 | 0.7296 | 0.3943 |
| 153 | 0.6047 | 0.7935 | 0.7306 | 0.3965 |
| 154 | 0.6032 | 0.7942 | 0.7306 | 0.3961 |
| 155 | 0.6047 | 0.7935 | 0.7306 | 0.3965 |
| 156 | 0.6077 | 0.7972 | 0.734 | 0.4035 |
| 157 | 0.6062 | 0.8001 | 0.7355 | 0.4057 |
| 158 | 0.6032 | 0.8016 | 0.7355 | 0.4049 |
| 159 | 0.6047 | 0.8001 | 0.735 | 0.4044 |
| 160 | 0.6047 | 0.8001 | 0.735 | 0.4044 |
| 161 | 0.6077 | 0.7994 | 0.7355 | 0.4062 |
| 162 | 0.6121 | 0.8009 | 0.738 | 0.4119 |
| 163 | 0.6136 | 0.8009 | 0.7384 | 0.4133 |
| 164 | 0.6136 | 0.8016 | 0.7389 | 0.4141 |
| 165 | 0.6165 | 0.8016 | 0.7399 | 0.4168 |
| 166 | 0.6136 | 0.8001 | 0.738 | 0.4124 |
| 167 | 0.615 | 0.8009 | 0.7389 | 0.4146 |
| 168 | 0.6121 | 0.7987 | 0.7365 | 0.4093 |
| 169 | 0.615 | 0.7965 | 0.736 | 0.4093 |
| 170 | 0.6106 | 0.792 | 0.7316 | 0.4001 |
| 171 | 0.6077 | 0.7942 | 0.7321 | 0.4 |
| 172 | 0.6121 | 0.795 | 0.734 | 0.4049 |
| 173 | 0.6209 | 0.8001 | 0.7404 | 0.419 |
| 174 | 0.615 | 0.795 | 0.735 | 0.4075 |
| 175 | 0.6136 | 0.7979 | 0.7365 | 0.4097 |
| 176 | 0.6106 | 0.7979 | 0.7355 | 0.4071 |
| 177 | 0.6091 | 0.7987 | 0.7355 | 0.4066 |
| 178 | 0.6121 | 0.8031 | 0.7394 | 0.4146 |
| 179 | 0.6106 | 0.8024 | 0.7384 | 0.4124 |
| 180 | 0.6106 | 0.8016 | 0.738 | 0.4115 |
| 181 | 0.6106 | 0.8038 | 0.7394 | 0.4141 |
| 182 | 0.615 | 0.8046 | 0.7414 | 0.419 |
| 183 | 0.6136 | 0.8038 | 0.7404 | 0.4168 |
| 184 | 0.6121 | 0.8016 | 0.7384 | 0.4128 |
| 185 | 0.6121 | 0.8038 | 0.7399 | 0.4155 |
| 186 | 0.6121 | 0.8038 | 0.7399 | 0.4155 |
| 187 | 0.6106 | 0.8053 | 0.7404 | 0.4159 |
| 188 | 0.6106 | 0.8024 | 0.7384 | 0.4124 |
| 189 | 0.6091 | 0.8031 | 0.7384 | 0.4119 |
| 190 | 0.6077 | 0.806 | 0.7399 | 0.4142 |
| 191 | 0.6062 | 0.8068 | 0.7399 | 0.4137 |
| 192 | 0.6018 | 0.8068 | 0.7384 | 0.4098 |
| 193 | 0.6018 | 0.8068 | 0.7384 | 0.4098 |
| 194 | 0.6062 | 0.8053 | 0.7389 | 0.412 |
| 195 | 0.6032 | 0.8038 | 0.737 | 0.4075 |
| 196 | 0.6047 | 0.806 | 0.7389 | 0.4115 |
| 197 | 0.6018 | 0.8083 | 0.7394 | 0.4116 |
| 198 | 0.6047 | 0.8046 | 0.738 | 0.4097 |
| 199 | 0.6047 | 0.8046 | 0.738 | 0.4097 |
| 200 | 0.6032 | 0.809 | 0.7404 | 0.4138 |
| 201 | 0.6077 | 0.8068 | 0.7404 | 0.4151 |
| 202 | 0.6106 | 0.8001 | 0.737 | 0.4097 |
| 203 | 0.6165 | 0.8083 | 0.7443 | 0.4248 |
| 204 | 0.615 | 0.8068 | 0.7429 | 0.4217 |
| 205 | 0.6091 | 0.806 | 0.7404 | 0.4155 |
| 206 | 0.6121 | 0.8038 | 0.7399 | 0.4155 |
| 207 | 0.615 | 0.8024 | 0.7399 | 0.4163 |
| 208 | 0.6195 | 0.806 | 0.7439 | 0.4247 |
| 209 | 0.618 | 0.8112 | 0.7468 | 0.4297 |
| 210 | 0.6195 | 0.8105 | 0.7468 | 0.4301 |
| 211 | 0.6283 | 0.809 | 0.7488 | 0.4362 |
| 212 | 0.6224 | 0.8075 | 0.7458 | 0.4292 |
| 213 | 0.618 | 0.8142 | 0.7488 | 0.4333 |
| 214 | 0.6195 | 0.8156 | 0.7502 | 0.4364 |
| 215 | 0.6195 | 0.8178 | 0.7517 | 0.4391 |
| 216 | 0.618 | 0.8127 | 0.7478 | 0.4315 |
| 217 | 0.6136 | 0.8134 | 0.7468 | 0.4284 |
| 218 | 0.6136 | 0.8142 | 0.7473 | 0.4293 |
| 219 | 0.618 | 0.8083 | 0.7448 | 0.4261 |
| 220 | 0.615 | 0.8075 | 0.7434 | 0.4226 |
| 221 | 0.6136 | 0.8038 | 0.7404 | 0.4168 |
| 222 | 0.615 | 0.8009 | 0.7389 | 0.4146 |
| 223 | 0.6165 | 0.8038 | 0.7414 | 0.4194 |
| 224 | 0.6165 | 0.8031 | 0.7409 | 0.4185 |
| 225 | 0.615 | 0.7987 | 0.7375 | 0.4119 |
| 226 | 0.6106 | 0.8001 | 0.737 | 0.4097 |
| 227 | 0.6106 | 0.8001 | 0.737 | 0.4097 |
| 228 | 0.618 | 0.7965 | 0.737 | 0.4119 |
| 229 | 0.6165 | 0.7965 | 0.7365 | 0.4106 |
| 230 | 0.6195 | 0.7972 | 0.738 | 0.4141 |
| 231 | 0.6239 | 0.7942 | 0.7375 | 0.4146 |
| 232 | 0.6239 | 0.795 | 0.738 | 0.4155 |
| 233 | 0.6239 | 0.7994 | 0.7409 | 0.4207 |
| 234 | 0.6268 | 0.8016 | 0.7434 | 0.426 |
| 235 | 0.6268 | 0.8024 | 0.7439 | 0.4269 |
| 236 | 0.6209 | 0.8009 | 0.7409 | 0.4199 |
| 237 | 0.6283 | 0.8046 | 0.7458 | 0.4309 |
| 238 | 0.6254 | 0.8031 | 0.7439 | 0.4265 |
| 239 | 0.6254 | 0.8016 | 0.7429 | 0.4247 |
| 240 | 0.618 | 0.8083 | 0.7448 | 0.4261 |
| 241 | 0.6165 | 0.8068 | 0.7434 | 0.423 |
| 242 | 0.6165 | 0.8068 | 0.7434 | 0.423 |
| 243 | 0.6165 | 0.806 | 0.7429 | 0.4221 |
| 244 | 0.6224 | 0.8068 | 0.7453 | 0.4283 |
| 245 | 0.6254 | 0.809 | 0.7478 | 0.4336 |
| 246 | 0.6254 | 0.8075 | 0.7468 | 0.4318 |
| 247 | 0.6239 | 0.8068 | 0.7458 | 0.4296 |
| 248 | 0.6254 | 0.8053 | 0.7453 | 0.4291 |
| 249 | 0.6209 | 0.8075 | 0.7453 | 0.4278 |
| 250 | 0.618 | 0.8053 | 0.7429 | 0.4225 |
| 251 | 0.6121 | 0.8046 | 0.7404 | 0.4164 |
| 252 | 0.6136 | 0.8068 | 0.7424 | 0.4204 |
| 253 | 0.6165 | 0.8075 | 0.7439 | 0.4239 |
| 254 | 0.615 | 0.8083 | 0.7439 | 0.4235 |
| 255 | 0.6136 | 0.8038 | 0.7404 | 0.4168 |
| 256 | 0.6136 | 0.8046 | 0.7409 | 0.4177 |
| 257 | 0.6136 | 0.8053 | 0.7414 | 0.4186 |
| 258 | 0.6136 | 0.8068 | 0.7424 | 0.4204 |
| 259 | 0.6136 | 0.8046 | 0.7409 | 0.4177 |
| 260 | 0.615 | 0.8046 | 0.7414 | 0.419 |
| 261 | 0.6165 | 0.8068 | 0.7434 | 0.423 |
| 262 | 0.6136 | 0.8083 | 0.7434 | 0.4221 |
| 263 | 0.6121 | 0.8075 | 0.7424 | 0.4199 |
| 264 | 0.6106 | 0.8068 | 0.7414 | 0.4177 |
| 265 | 0.6121 | 0.8134 | 0.7463 | 0.4271 |
| 266 | 0.6106 | 0.8112 | 0.7443 | 0.4231 |
| 267 | 0.6077 | 0.8068 | 0.7404 | 0.4151 |
| 268 | 0.6106 | 0.8053 | 0.7404 | 0.4159 |
| 269 | 0.6091 | 0.8001 | 0.7365 | 0.4084 |
| 270 | 0.6091 | 0.8016 | 0.7375 | 0.4102 |
| 271 | 0.6062 | 0.8038 | 0.738 | 0.4102 |
| 272 | 0.6077 | 0.8075 | 0.7409 | 0.416 |
| 273 | 0.618 | 0.806 | 0.7434 | 0.4234 |
| 274 | 0.618 | 0.8046 | 0.7424 | 0.4216 |
| 275 | 0.6195 | 0.8038 | 0.7424 | 0.4221 |
| 276 | 0.6165 | 0.8024 | 0.7404 | 0.4177 |
| 277 | 0.615 | 0.8024 | 0.7399 | 0.4163 |
| 278 | 0.6136 | 0.8031 | 0.7399 | 0.4159 |
| 279 | 0.6136 | 0.8053 | 0.7414 | 0.4186 |
| 280 | 0.6136 | 0.806 | 0.7419 | 0.4195 |
| 281 | 0.6224 | 0.8083 | 0.7463 | 0.43 |
| 282 | 0.6209 | 0.8075 | 0.7453 | 0.4278 |
| 283 | 0.6224 | 0.8068 | 0.7453 | 0.4283 |
| 284 | 0.6209 | 0.8038 | 0.7429 | 0.4234 |
| 285 | 0.6195 | 0.8038 | 0.7424 | 0.4221 |
| 286 | 0.6195 | 0.806 | 0.7439 | 0.4247 |
| 287 | 0.6121 | 0.806 | 0.7414 | 0.4181 |
| 288 | 0.618 | 0.8031 | 0.7414 | 0.4199 |
| 289 | 0.6195 | 0.8046 | 0.7429 | 0.423 |
| 290 | 0.618 | 0.8031 | 0.7414 | 0.4199 |
| 291 | 0.6165 | 0.8031 | 0.7409 | 0.4185 |
| 292 | 0.615 | 0.8046 | 0.7414 | 0.419 |
| 293 | 0.6003 | 0.8068 | 0.738 | 0.4085 |
| 294 | 0.6003 | 0.8097 | 0.7399 | 0.412 |
| 295 | 0.6018 | 0.8075 | 0.7389 | 0.4107 |
| 296 | 0.5988 | 0.8068 | 0.7375 | 0.4071 |
| 297 | 0.5988 | 0.8068 | 0.7375 | 0.4071 |
| 298 | 0.6003 | 0.806 | 0.7375 | 0.4076 |
| 299 | 0.5944 | 0.8075 | 0.7365 | 0.4041 |
| 300 | 0.5944 | 0.8119 | 0.7394 | 0.4095 |
| 301 | 0.5944 | 0.8112 | 0.7389 | 0.4086 |
| 302 | 0.59 | 0.8105 | 0.737 | 0.4037 |
| 303 | 0.59 | 0.8105 | 0.737 | 0.4037 |
| 304 | 0.5885 | 0.809 | 0.7355 | 0.4005 |
| 305 | 0.587 | 0.8171 | 0.7404 | 0.4092 |
| 306 | 0.5929 | 0.8149 | 0.7409 | 0.4118 |
| 307 | 0.5944 | 0.8142 | 0.7409 | 0.4122 |
| 308 | 0.5944 | 0.8156 | 0.7419 | 0.414 |
| 309 | 0.5929 | 0.8149 | 0.7409 | 0.4118 |
| 310 | 0.5944 | 0.8142 | 0.7409 | 0.4122 |
| 311 | 0.5959 | 0.8149 | 0.7419 | 0.4144 |
| 312 | 0.5959 | 0.8164 | 0.7429 | 0.4162 |
| 313 | 0.5959 | 0.8142 | 0.7414 | 0.4135 |
| 314 | 0.5914 | 0.8134 | 0.7394 | 0.4086 |
| 315 | 0.5929 | 0.8149 | 0.7409 | 0.4118 |
| 316 | 0.5959 | 0.8156 | 0.7424 | 0.4153 |
| 317 | 0.5959 | 0.8164 | 0.7429 | 0.4162 |
| 318 | 0.5959 | 0.8142 | 0.7414 | 0.4135 |
| 319 | 0.5959 | 0.8149 | 0.7419 | 0.4144 |
| 320 | 0.5929 | 0.8164 | 0.7419 | 0.4136 |
| 321 | 0.5929 | 0.8186 | 0.7434 | 0.4163 |
| 322 | 0.5944 | 0.8208 | 0.7453 | 0.4204 |
| 323 | 0.5929 | 0.8201 | 0.7443 | 0.4182 |
| 324 | 0.5929 | 0.8215 | 0.7453 | 0.42 |
| 325 | 0.5929 | 0.8171 | 0.7424 | 0.4145 |
| 326 | 0.5929 | 0.8156 | 0.7414 | 0.4127 |
| 327 | 0.5944 | 0.8164 | 0.7424 | 0.4149 |
| 328 | 0.6003 | 0.8171 | 0.7448 | 0.4211 |
| 329 | 0.6018 | 0.8164 | 0.7448 | 0.4215 |
| 330 | 0.6062 | 0.8178 | 0.7473 | 0.4273 |
| 331 | 0.6018 | 0.8186 | 0.7463 | 0.4243 |
| 332 | 0.5988 | 0.8149 | 0.7429 | 0.4171 |
| 333 | 0.6018 | 0.8178 | 0.7458 | 0.4233 |
| 334 | 0.5959 | 0.8171 | 0.7434 | 0.4172 |
| 335 | 0.5959 | 0.8171 | 0.7434 | 0.4172 |
| 336 | 0.5959 | 0.8201 | 0.7453 | 0.4208 |
| 337 | 0.5959 | 0.8171 | 0.7434 | 0.4172 |
| 338 | 0.5959 | 0.8156 | 0.7424 | 0.4153 |
| 339 | 0.5944 | 0.8149 | 0.7414 | 0.4131 |
| 340 | 0.5944 | 0.8134 | 0.7404 | 0.4113 |
| 341 | 0.5944 | 0.8134 | 0.7404 | 0.4113 |
| 342 | 0.5944 | 0.8134 | 0.7404 | 0.4113 |
| 343 | 0.5929 | 0.8156 | 0.7414 | 0.4127 |
| 344 | 0.5944 | 0.8112 | 0.7389 | 0.4086 |
| 345 | 0.5929 | 0.8112 | 0.7384 | 0.4072 |
| 346 | 0.5944 | 0.8105 | 0.7384 | 0.4077 |
| 347 | 0.5929 | 0.8127 | 0.7394 | 0.409 |
| 348 | 0.5929 | 0.8127 | 0.7394 | 0.409 |
| 349 | 0.6032 | 0.8083 | 0.7399 | 0.4129 |
| 350 | 0.6091 | 0.806 | 0.7404 | 0.4155 |
| 351 | 0.6091 | 0.8075 | 0.7414 | 0.4173 |
| 352 | 0.6091 | 0.8083 | 0.7419 | 0.4182 |
| 353 | 0.6091 | 0.8053 | 0.7399 | 0.4146 |
| 354 | 0.6091 | 0.8083 | 0.7419 | 0.4182 |
| 355 | 0.6077 | 0.8075 | 0.7409 | 0.416 |
| 356 | 0.6077 | 0.8105 | 0.7429 | 0.4196 |
| 357 | 0.6047 | 0.8097 | 0.7414 | 0.416 |
| 358 | 0.6018 | 0.8068 | 0.7384 | 0.4098 |
| 359 | 0.6018 | 0.8075 | 0.7389 | 0.4107 |
| 360 | 0.6018 | 0.8083 | 0.7394 | 0.4116 |
| 361 | 0.6003 | 0.8068 | 0.738 | 0.4085 |
| 362 | 0.5973 | 0.8038 | 0.735 | 0.4022 |
| 363 | 0.6018 | 0.8053 | 0.7375 | 0.408 |
| 364 | 0.6077 | 0.8046 | 0.7389 | 0.4124 |
| 365 | 0.6091 | 0.8053 | 0.7399 | 0.4146 |
| 366 | 0.6091 | 0.8075 | 0.7414 | 0.4173 |
| 367 | 0.6047 | 0.8112 | 0.7424 | 0.4178 |
| 368 | 0.6047 | 0.8097 | 0.7414 | 0.416 |
| 369 | 0.6047 | 0.8083 | 0.7404 | 0.4142 |
| 370 | 0.6121 | 0.8119 | 0.7453 | 0.4253 |
| 371 | 0.615 | 0.8149 | 0.7483 | 0.4316 |
| 372 | 0.6121 | 0.8127 | 0.7458 | 0.4262 |
| 373 | 0.6106 | 0.8119 | 0.7448 | 0.424 |
| 374 | 0.6121 | 0.8127 | 0.7458 | 0.4262 |
| 375 | 0.6106 | 0.8105 | 0.7439 | 0.4222 |
| 376 | 0.6106 | 0.809 | 0.7429 | 0.4204 |
| 377 | 0.6091 | 0.809 | 0.7424 | 0.4191 |
| 378 | 0.6062 | 0.806 | 0.7394 | 0.4129 |
| 379 | 0.6018 | 0.806 | 0.738 | 0.4089 |
| 380 | 0.6032 | 0.8046 | 0.7375 | 0.4084 |
| 381 | 0.6032 | 0.8053 | 0.738 | 0.4093 |
| 382 | 0.6032 | 0.8031 | 0.7365 | 0.4066 |
| 383 | 0.6091 | 0.8068 | 0.7409 | 0.4164 |
| 384 | 0.6077 | 0.806 | 0.7399 | 0.4142 |
| 385 | 0.6032 | 0.8068 | 0.7389 | 0.4111 |
| 386 | 0.6106 | 0.8083 | 0.7424 | 0.4195 |
| 387 | 0.6091 | 0.8083 | 0.7419 | 0.4182 |
| 388 | 0.6106 | 0.8075 | 0.7419 | 0.4186 |
| 389 | 0.6121 | 0.8068 | 0.7419 | 0.419 |
| 390 | 0.6091 | 0.8053 | 0.7399 | 0.4146 |
| 391 | 0.6062 | 0.8053 | 0.7389 | 0.412 |
| 392 | 0.6062 | 0.8046 | 0.7384 | 0.4111 |
| 393 | 0.6062 | 0.8053 | 0.7389 | 0.412 |
| 394 | 0.6062 | 0.806 | 0.7394 | 0.4129 |
| 395 | 0.6047 | 0.8046 | 0.738 | 0.4097 |
| 396 | 0.6032 | 0.8016 | 0.7355 | 0.4049 |
| 397 | 0.6032 | 0.806 | 0.7384 | 0.4102 |
| 398 | 0.6047 | 0.8075 | 0.7399 | 0.4133 |
| 399 | 0.6018 | 0.8105 | 0.7409 | 0.4143 |
| 400 | 0.6018 | 0.8053 | 0.7375 | 0.408 |
| 401 | 0.6003 | 0.8075 | 0.7384 | 0.4094 |
| 402 | 0.5988 | 0.8053 | 0.7365 | 0.4053 |
| 403 | 0.5973 | 0.806 | 0.7365 | 0.4049 |
| 404 | 0.5988 | 0.806 | 0.737 | 0.4062 |
| 405 | 0.5988 | 0.8105 | 0.7399 | 0.4116 |
| 406 | 0.6003 | 0.809 | 0.7394 | 0.4111 |
| 407 | 0.6003 | 0.8097 | 0.7399 | 0.412 |
| 408 | 0.6018 | 0.8083 | 0.7394 | 0.4116 |
| 409 | 0.5988 | 0.8097 | 0.7394 | 0.4107 |
| 410 | 0.5988 | 0.8068 | 0.7375 | 0.4071 |
| 411 | 0.5973 | 0.8075 | 0.7375 | 0.4067 |
| 412 | 0.6018 | 0.806 | 0.738 | 0.4089 |
| 413 | 0.5988 | 0.8031 | 0.735 | 0.4027 |
| 414 | 0.5973 | 0.8031 | 0.7345 | 0.4013 |
| 415 | 0.6003 | 0.8031 | 0.7355 | 0.404 |
| 416 | 0.6003 | 0.8016 | 0.7345 | 0.4022 |
| 417 | 0.6047 | 0.8097 | 0.7414 | 0.416 |
| 418 | 0.6018 | 0.8083 | 0.7394 | 0.4116 |
| 419 | 0.6003 | 0.8068 | 0.738 | 0.4085 |
| 420 | 0.6018 | 0.8068 | 0.7384 | 0.4098 |
| 421 | 0.6032 | 0.8075 | 0.7394 | 0.412 |
| 422 | 0.6032 | 0.8112 | 0.7419 | 0.4165 |
| 423 | 0.6032 | 0.8112 | 0.7419 | 0.4165 |
| 424 | 0.6003 | 0.8134 | 0.7424 | 0.4166 |
| 425 | 0.6032 | 0.8142 | 0.7439 | 0.4201 |
| 426 | 0.6032 | 0.8149 | 0.7443 | 0.421 |
| 427 | 0.6003 | 0.8142 | 0.7429 | 0.4175 |
| 428 | 0.6018 | 0.8127 | 0.7424 | 0.417 |
| 429 | 0.6018 | 0.8119 | 0.7419 | 0.4161 |
| 430 | 0.5973 | 0.8127 | 0.7409 | 0.413 |
| 431 | 0.6003 | 0.8119 | 0.7414 | 0.4148 |
| 432 | 0.6003 | 0.8127 | 0.7419 | 0.4157 |
| 433 | 0.5959 | 0.8119 | 0.7399 | 0.4108 |
| 434 | 0.5944 | 0.8127 | 0.7399 | 0.4104 |
| 435 | 0.5929 | 0.8149 | 0.7409 | 0.4118 |
| 436 | 0.5929 | 0.8142 | 0.7404 | 0.4109 |
| 437 | 0.5929 | 0.8149 | 0.7409 | 0.4118 |
| 438 | 0.59 | 0.8127 | 0.7384 | 0.4064 |
| 439 | 0.59 | 0.8149 | 0.7399 | 0.4091 |
| 440 | 0.5914 | 0.8134 | 0.7394 | 0.4086 |
| 441 | 0.59 | 0.8127 | 0.7384 | 0.4064 |
| 442 | 0.59 | 0.8134 | 0.7389 | 0.4073 |
| 443 | 0.587 | 0.8134 | 0.738 | 0.4047 |
| 444 | 0.59 | 0.8142 | 0.7394 | 0.4082 |
| 445 | 0.5885 | 0.8134 | 0.7384 | 0.406 |
| 446 | 0.5885 | 0.8119 | 0.7375 | 0.4042 |
| 447 | 0.5885 | 0.8119 | 0.7375 | 0.4042 |
| 448 | 0.5885 | 0.8127 | 0.738 | 0.4051 |
| 449 | 0.59 | 0.8142 | 0.7394 | 0.4082 |
| 450 | 0.5885 | 0.8149 | 0.7394 | 0.4078 |
| 451 | 0.587 | 0.8149 | 0.7389 | 0.4065 |
| 452 | 0.5885 | 0.8142 | 0.7389 | 0.4069 |
| 453 | 0.5885 | 0.8149 | 0.7394 | 0.4078 |
| 454 | 0.59 | 0.8149 | 0.7399 | 0.4091 |
| 455 | 0.5914 | 0.8134 | 0.7394 | 0.4086 |
| 456 | 0.5914 | 0.8134 | 0.7394 | 0.4086 |
| 457 | 0.5929 | 0.8119 | 0.7389 | 0.4081 |
| 458 | 0.5929 | 0.8156 | 0.7414 | 0.4127 |
| 459 | 0.5914 | 0.8193 | 0.7434 | 0.4159 |
| 460 | 0.5914 | 0.8178 | 0.7424 | 0.4141 |
| 461 | 0.59 | 0.8193 | 0.7429 | 0.4146 |
| 462 | 0.59 | 0.8201 | 0.7434 | 0.4155 |
| 463 | 0.5929 | 0.8215 | 0.7453 | 0.42 |
| 464 | 0.5929 | 0.8193 | 0.7439 | 0.4173 |
| 465 | 0.5944 | 0.8164 | 0.7424 | 0.4149 |
| 466 | 0.5959 | 0.8193 | 0.7448 | 0.4199 |
| 467 | 0.5973 | 0.8208 | 0.7463 | 0.4231 |
| 468 | 0.5988 | 0.8215 | 0.7473 | 0.4253 |
| 469 | 0.5944 | 0.823 | 0.7468 | 0.4232 |
| 470 | 0.5929 | 0.8237 | 0.7468 | 0.4228 |
| 471 | 0.5914 | 0.8223 | 0.7453 | 0.4196 |
| 472 | 0.59 | 0.8208 | 0.7439 | 0.4165 |
| 473 | 0.5885 | 0.8178 | 0.7414 | 0.4115 |
| 474 | 0.587 | 0.8164 | 0.7399 | 0.4083 |
| 475 | 0.59 | 0.8164 | 0.7409 | 0.411 |
| 476 | 0.5914 | 0.8178 | 0.7424 | 0.4141 |
| 477 | 0.5914 | 0.8178 | 0.7424 | 0.4141 |
| 478 | 0.5944 | 0.8164 | 0.7424 | 0.4149 |
| 479 | 0.5944 | 0.8142 | 0.7409 | 0.4122 |
| 480 | 0.5914 | 0.8134 | 0.7394 | 0.4086 |
| 481 | 0.5929 | 0.8156 | 0.7414 | 0.4127 |
| 482 | 0.6003 | 0.8142 | 0.7429 | 0.4175 |
| 483 | 0.6003 | 0.8142 | 0.7429 | 0.4175 |
| 484 | 0.5988 | 0.8142 | 0.7424 | 0.4162 |
| 485 | 0.5973 | 0.8142 | 0.7419 | 0.4148 |
| 486 | 0.5973 | 0.8142 | 0.7419 | 0.4148 |
| 487 | 0.5959 | 0.8149 | 0.7419 | 0.4144 |
| 488 | 0.5959 | 0.8119 | 0.7399 | 0.4108 |
| 489 | 0.5929 | 0.8097 | 0.7375 | 0.4054 |
| 490 | 0.5929 | 0.8112 | 0.7384 | 0.4072 |
| 491 | 0.5914 | 0.8112 | 0.738 | 0.4059 |
| 492 | 0.5914 | 0.8112 | 0.738 | 0.4059 |
| 493 | 0.5944 | 0.8097 | 0.738 | 0.4068 |
| 494 | 0.5929 | 0.8083 | 0.7365 | 0.4036 |
| 495 | 0.5973 | 0.8127 | 0.7409 | 0.413 |
| 496 | 0.5973 | 0.8127 | 0.7409 | 0.413 |
| 497 | 0.5988 | 0.8127 | 0.7414 | 0.4143 |
| 498 | 0.6003 | 0.8119 | 0.7414 | 0.4148 |
| 499 | 0.6003 | 0.8112 | 0.7409 | 0.4139 |
| 500 | 0.6003 | 0.8127 | 0.7419 | 0.4157 |
