# Supplementary material for: Prediction and Analysis of Protein Hydroxyproline and Hydroxylysine
Source: PLoS One. 2010 Dec 31;5(12):e15917. doi: 10.1371/journal.pone.0015917 (PMC3013141; doi:10.1371/journal.pone.0015917)
Supplement: Table S6 — Performance of 500 NNA predictors for hydroxylysine dataset. (DOC) [file pone.0015917.s006.doc]

**Table S6. Performance of 500 NNA predictors for hydroxylysine dataset**

Listed below are the Sensitivity (Sn), Specificity (Sp), Accuracy (AC) and Mathew correlation coefficient (MCC) of 500 predictors for hydroxylysine dataset constructed by nearest neighbor algorithm and evaluated by Jackknife test.

| Index | Sn | Sp | AC | MCC |
| --- | --- | --- | --- | --- |
| 1 | 1 | 0 | 0.3333 | - |
| 2 | 0.9352 | 0.625 | 0.7284 | 0.5322 |
| 3 | 0.8519 | 0.7731 | 0.7994 | 0.5943 |
| 4 | 0.8333 | 0.7685 | 0.7901 | 0.5727 |
| 5 | 0.8519 | 0.7731 | 0.7994 | 0.5943 |
| 6 | 0.8519 | 0.7778 | 0.8025 | 0.5992 |
| 7 | 0.7222 | 0.787 | 0.7654 | 0.4939 |
| 8 | 0.7222 | 0.7917 | 0.7685 | 0.4992 |
| 9 | 0.6574 | 0.7824 | 0.7407 | 0.4309 |
| 10 | 0.6574 | 0.787 | 0.7438 | 0.4362 |
| 11 | 0.6759 | 0.8148 | 0.7685 | 0.4854 |
| 12 | 0.6296 | 0.8333 | 0.7654 | 0.4675 |
| 13 | 0.6389 | 0.8611 | 0.787 | 0.5117 |
| 14 | 0.6296 | 0.8611 | 0.784 | 0.5036 |
| 15 | 0.6111 | 0.8843 | 0.7932 | 0.5196 |
| 16 | 0.6667 | 0.8565 | 0.7932 | 0.5296 |
| 17 | 0.6759 | 0.8611 | 0.7994 | 0.5437 |
| 18 | 0.6759 | 0.8657 | 0.8025 | 0.5498 |
| 19 | 0.6667 | 0.8565 | 0.7932 | 0.5296 |
| 20 | 0.6574 | 0.8519 | 0.787 | 0.5155 |
| 21 | 0.6667 | 0.8611 | 0.7963 | 0.5357 |
| 22 | 0.6759 | 0.8565 | 0.7963 | 0.5376 |
| 23 | 0.6574 | 0.8657 | 0.7963 | 0.5339 |
| 24 | 0.6481 | 0.8657 | 0.7932 | 0.5259 |
| 25 | 0.6389 | 0.8519 | 0.7809 | 0.4994 |
| 26 | 0.6481 | 0.8611 | 0.7901 | 0.5197 |
| 27 | 0.6667 | 0.8704 | 0.8025 | 0.548 |
| 28 | 0.6574 | 0.875 | 0.8025 | 0.5464 |
| 29 | 0.6574 | 0.8796 | 0.8056 | 0.5528 |
| 30 | 0.6667 | 0.8843 | 0.8117 | 0.5671 |
| 31 | 0.6667 | 0.8843 | 0.8117 | 0.5671 |
| 32 | 0.6759 | 0.8889 | 0.8179 | 0.5814 |
| 33 | 0.6667 | 0.875 | 0.8056 | 0.5543 |
| 34 | 0.6667 | 0.875 | 0.8056 | 0.5543 |
| 35 | 0.6759 | 0.8843 | 0.8148 | 0.5749 |
| 36 | 0.6574 | 0.8796 | 0.8056 | 0.5528 |
| 37 | 0.6667 | 0.8796 | 0.8086 | 0.5607 |
| 38 | 0.6667 | 0.8796 | 0.8086 | 0.5607 |
| 39 | 0.6574 | 0.8843 | 0.8086 | 0.5592 |
| 40 | 0.6574 | 0.8796 | 0.8056 | 0.5528 |
| 41 | 0.6944 | 0.8796 | 0.8179 | 0.5842 |
| 42 | 0.7037 | 0.8796 | 0.821 | 0.5921 |
| 43 | 0.7037 | 0.8796 | 0.821 | 0.5921 |
| 44 | 0.6852 | 0.8843 | 0.8179 | 0.5827 |
| 45 | 0.6481 | 0.8796 | 0.8025 | 0.5449 |
| 46 | 0.6574 | 0.8843 | 0.8086 | 0.5592 |
| 47 | 0.6481 | 0.8796 | 0.8025 | 0.5449 |
| 48 | 0.6481 | 0.8796 | 0.8025 | 0.5449 |
| 49 | 0.6389 | 0.8935 | 0.8086 | 0.5566 |
| 50 | 0.6481 | 0.8704 | 0.7963 | 0.5322 |
| 51 | 0.6481 | 0.8611 | 0.7901 | 0.5197 |
| 52 | 0.6481 | 0.8611 | 0.7901 | 0.5197 |
| 53 | 0.6389 | 0.8704 | 0.7932 | 0.5242 |
| 54 | 0.6389 | 0.875 | 0.7963 | 0.5305 |
| 55 | 0.6204 | 0.8565 | 0.7778 | 0.4894 |
| 56 | 0.6296 | 0.8935 | 0.8056 | 0.5487 |
| 57 | 0.6204 | 0.8889 | 0.7994 | 0.5342 |
| 58 | 0.6204 | 0.8889 | 0.7994 | 0.5342 |
| 59 | 0.6204 | 0.8935 | 0.8025 | 0.5409 |
| 60 | 0.6111 | 0.8935 | 0.7994 | 0.5329 |
| 61 | 0.6111 | 0.8981 | 0.8025 | 0.5397 |
| 62 | 0.5926 | 0.8981 | 0.7963 | 0.5239 |
| 63 | 0.5926 | 0.8981 | 0.7963 | 0.5239 |
| 64 | 0.6111 | 0.9028 | 0.8056 | 0.5466 |
| 65 | 0.6204 | 0.8981 | 0.8056 | 0.5476 |
| 66 | 0.6204 | 0.8981 | 0.8056 | 0.5476 |
| 67 | 0.6204 | 0.8935 | 0.8025 | 0.5409 |
| 68 | 0.6204 | 0.8935 | 0.8025 | 0.5409 |
| 69 | 0.6204 | 0.8935 | 0.8025 | 0.5409 |
| 70 | 0.6389 | 0.8843 | 0.8025 | 0.5434 |
| 71 | 0.6296 | 0.8843 | 0.7994 | 0.5355 |
| 72 | 0.6481 | 0.8889 | 0.8086 | 0.5578 |
| 73 | 0.6481 | 0.8889 | 0.8086 | 0.5578 |
| 74 | 0.6481 | 0.8889 | 0.8086 | 0.5578 |
| 75 | 0.6481 | 0.8889 | 0.8086 | 0.5578 |
| 76 | 0.6481 | 0.8889 | 0.8086 | 0.5578 |
| 77 | 0.6111 | 0.8935 | 0.7994 | 0.5329 |
| 78 | 0.6111 | 0.8935 | 0.7994 | 0.5329 |
| 79 | 0.6111 | 0.8935 | 0.7994 | 0.5329 |
| 80 | 0.6111 | 0.8935 | 0.7994 | 0.5329 |
| 81 | 0.6111 | 0.8935 | 0.7994 | 0.5329 |
| 82 | 0.6574 | 0.9028 | 0.821 | 0.5856 |
| 83 | 0.6296 | 0.8981 | 0.8086 | 0.5555 |
| 84 | 0.6389 | 0.8981 | 0.8117 | 0.5633 |
| 85 | 0.6296 | 0.8981 | 0.8086 | 0.5555 |
| 86 | 0.6389 | 0.8981 | 0.8117 | 0.5633 |
| 87 | 0.6389 | 0.9028 | 0.8148 | 0.5701 |
| 88 | 0.6389 | 0.9028 | 0.8148 | 0.5701 |
| 89 | 0.6389 | 0.9028 | 0.8148 | 0.5701 |
| 90 | 0.6204 | 0.8981 | 0.8056 | 0.5476 |
| 91 | 0.6204 | 0.8935 | 0.8025 | 0.5409 |
| 92 | 0.6204 | 0.8889 | 0.7994 | 0.5342 |
| 93 | 0.6204 | 0.8935 | 0.8025 | 0.5409 |
| 94 | 0.6204 | 0.8935 | 0.8025 | 0.5409 |
| 95 | 0.6111 | 0.8981 | 0.8025 | 0.5397 |
| 96 | 0.6111 | 0.8935 | 0.7994 | 0.5329 |
| 97 | 0.6111 | 0.8981 | 0.8025 | 0.5397 |
| 98 | 0.6111 | 0.8981 | 0.8025 | 0.5397 |
| 99 | 0.6019 | 0.8981 | 0.7994 | 0.5318 |
| 100 | 0.6204 | 0.8889 | 0.7994 | 0.5342 |
| 101 | 0.6204 | 0.8796 | 0.7932 | 0.521 |
| 102 | 0.6389 | 0.8704 | 0.7932 | 0.5242 |
| 103 | 0.6389 | 0.8704 | 0.7932 | 0.5242 |
| 104 | 0.6389 | 0.8704 | 0.7932 | 0.5242 |
| 105 | 0.6389 | 0.8704 | 0.7932 | 0.5242 |
| 106 | 0.6389 | 0.8796 | 0.7994 | 0.5369 |
| 107 | 0.6481 | 0.8796 | 0.8025 | 0.5449 |
| 108 | 0.6481 | 0.8796 | 0.8025 | 0.5449 |
| 109 | 0.6296 | 0.875 | 0.7932 | 0.5226 |
| 110 | 0.6019 | 0.8704 | 0.7809 | 0.4921 |
| 111 | 0.6019 | 0.875 | 0.784 | 0.4985 |
| 112 | 0.6019 | 0.8704 | 0.7809 | 0.4921 |
| 113 | 0.6019 | 0.8704 | 0.7809 | 0.4921 |
| 114 | 0.5926 | 0.8657 | 0.7747 | 0.4776 |
| 115 | 0.5926 | 0.8657 | 0.7747 | 0.4776 |
| 116 | 0.6019 | 0.875 | 0.784 | 0.4985 |
| 117 | 0.6019 | 0.8704 | 0.7809 | 0.4921 |
| 118 | 0.5926 | 0.875 | 0.7809 | 0.4905 |
| 119 | 0.5926 | 0.875 | 0.7809 | 0.4905 |
| 120 | 0.5926 | 0.875 | 0.7809 | 0.4905 |
| 121 | 0.5926 | 0.875 | 0.7809 | 0.4905 |
| 122 | 0.5926 | 0.875 | 0.7809 | 0.4905 |
| 123 | 0.6111 | 0.8611 | 0.7778 | 0.4875 |
| 124 | 0.6111 | 0.8611 | 0.7778 | 0.4875 |
| 125 | 0.6111 | 0.8611 | 0.7778 | 0.4875 |
| 126 | 0.6019 | 0.8611 | 0.7747 | 0.4794 |
| 127 | 0.6019 | 0.8704 | 0.7809 | 0.4921 |
| 128 | 0.5926 | 0.8657 | 0.7747 | 0.4776 |
| 129 | 0.5926 | 0.8657 | 0.7747 | 0.4776 |
| 130 | 0.5926 | 0.8704 | 0.7778 | 0.484 |
| 131 | 0.6019 | 0.8657 | 0.7778 | 0.4857 |
| 132 | 0.6019 | 0.8657 | 0.7778 | 0.4857 |
| 133 | 0.6111 | 0.8704 | 0.784 | 0.5001 |
| 134 | 0.6111 | 0.8704 | 0.784 | 0.5001 |
| 135 | 0.6019 | 0.8704 | 0.7809 | 0.4921 |
| 136 | 0.6019 | 0.8704 | 0.7809 | 0.4921 |
| 137 | 0.6019 | 0.8704 | 0.7809 | 0.4921 |
| 138 | 0.6111 | 0.8704 | 0.784 | 0.5001 |
| 139 | 0.6111 | 0.8704 | 0.784 | 0.5001 |
| 140 | 0.6111 | 0.8704 | 0.784 | 0.5001 |
| 141 | 0.6111 | 0.8704 | 0.784 | 0.5001 |
| 142 | 0.6019 | 0.8657 | 0.7778 | 0.4857 |
| 143 | 0.6019 | 0.8657 | 0.7778 | 0.4857 |
| 144 | 0.6019 | 0.8657 | 0.7778 | 0.4857 |
| 145 | 0.6019 | 0.8657 | 0.7778 | 0.4857 |
| 146 | 0.6019 | 0.875 | 0.784 | 0.4985 |
| 147 | 0.6204 | 0.8704 | 0.787 | 0.5082 |
| 148 | 0.6111 | 0.8657 | 0.7809 | 0.4938 |
| 149 | 0.6111 | 0.8657 | 0.7809 | 0.4938 |
| 150 | 0.6111 | 0.8657 | 0.7809 | 0.4938 |
| 151 | 0.6111 | 0.8704 | 0.784 | 0.5001 |
| 152 | 0.6111 | 0.8704 | 0.784 | 0.5001 |
| 153 | 0.6111 | 0.8704 | 0.784 | 0.5001 |
| 154 | 0.6111 | 0.8704 | 0.784 | 0.5001 |
| 155 | 0.6296 | 0.8657 | 0.787 | 0.5099 |
| 156 | 0.6296 | 0.8657 | 0.787 | 0.5099 |
| 157 | 0.6296 | 0.8657 | 0.787 | 0.5099 |
| 158 | 0.6296 | 0.8704 | 0.7901 | 0.5162 |
| 159 | 0.6296 | 0.8704 | 0.7901 | 0.5162 |
| 160 | 0.6204 | 0.875 | 0.7901 | 0.5146 |
| 161 | 0.6204 | 0.875 | 0.7901 | 0.5146 |
| 162 | 0.6296 | 0.875 | 0.7932 | 0.5226 |
| 163 | 0.6389 | 0.875 | 0.7963 | 0.5305 |
| 164 | 0.6389 | 0.875 | 0.7963 | 0.5305 |
| 165 | 0.6389 | 0.875 | 0.7963 | 0.5305 |
| 166 | 0.6389 | 0.875 | 0.7963 | 0.5305 |
| 167 | 0.6389 | 0.8704 | 0.7932 | 0.5242 |
| 168 | 0.6296 | 0.8611 | 0.784 | 0.5036 |
| 169 | 0.6296 | 0.8611 | 0.784 | 0.5036 |
| 170 | 0.6296 | 0.8611 | 0.784 | 0.5036 |
| 171 | 0.6389 | 0.8657 | 0.7901 | 0.5179 |
| 172 | 0.6389 | 0.8657 | 0.7901 | 0.5179 |
| 173 | 0.6296 | 0.8611 | 0.784 | 0.5036 |
| 174 | 0.6296 | 0.8611 | 0.784 | 0.5036 |
| 175 | 0.6296 | 0.8611 | 0.784 | 0.5036 |
| 176 | 0.6389 | 0.8704 | 0.7932 | 0.5242 |
| 177 | 0.6296 | 0.8704 | 0.7901 | 0.5162 |
| 178 | 0.6296 | 0.8611 | 0.784 | 0.5036 |
| 179 | 0.6389 | 0.8611 | 0.787 | 0.5117 |
| 180 | 0.6389 | 0.8611 | 0.787 | 0.5117 |
| 181 | 0.6389 | 0.8611 | 0.787 | 0.5117 |
| 182 | 0.6389 | 0.8611 | 0.787 | 0.5117 |
| 183 | 0.6204 | 0.8519 | 0.7747 | 0.4833 |
| 184 | 0.6204 | 0.8519 | 0.7747 | 0.4833 |
| 185 | 0.6204 | 0.8519 | 0.7747 | 0.4833 |
| 186 | 0.6204 | 0.8519 | 0.7747 | 0.4833 |
| 187 | 0.6204 | 0.8519 | 0.7747 | 0.4833 |
| 188 | 0.6204 | 0.8519 | 0.7747 | 0.4833 |
| 189 | 0.6204 | 0.8519 | 0.7747 | 0.4833 |
| 190 | 0.5741 | 0.8565 | 0.7623 | 0.4487 |
| 191 | 0.5741 | 0.8565 | 0.7623 | 0.4487 |
| 192 | 0.5741 | 0.8565 | 0.7623 | 0.4487 |
| 193 | 0.5648 | 0.8565 | 0.7593 | 0.4404 |
| 194 | 0.5648 | 0.8611 | 0.7623 | 0.4468 |
| 195 | 0.5556 | 0.8565 | 0.7562 | 0.4322 |
| 196 | 0.5556 | 0.8565 | 0.7562 | 0.4322 |
| 197 | 0.5556 | 0.8565 | 0.7562 | 0.4322 |
| 198 | 0.5648 | 0.8565 | 0.7593 | 0.4404 |
| 199 | 0.5648 | 0.8565 | 0.7593 | 0.4404 |
| 200 | 0.5648 | 0.8565 | 0.7593 | 0.4404 |
| 201 | 0.5648 | 0.8565 | 0.7593 | 0.4404 |
| 202 | 0.5556 | 0.8657 | 0.7623 | 0.4449 |
| 203 | 0.5648 | 0.8704 | 0.7685 | 0.4596 |
| 204 | 0.5648 | 0.8704 | 0.7685 | 0.4596 |
| 205 | 0.5648 | 0.8704 | 0.7685 | 0.4596 |
| 206 | 0.5648 | 0.8704 | 0.7685 | 0.4596 |
| 207 | 0.5556 | 0.8611 | 0.7593 | 0.4385 |
| 208 | 0.5556 | 0.8611 | 0.7593 | 0.4385 |
| 209 | 0.5556 | 0.8565 | 0.7562 | 0.4322 |
| 210 | 0.5556 | 0.8657 | 0.7623 | 0.4449 |
| 211 | 0.5648 | 0.8611 | 0.7623 | 0.4468 |
| 212 | 0.5648 | 0.8611 | 0.7623 | 0.4468 |
| 213 | 0.5741 | 0.8611 | 0.7654 | 0.455 |
| 214 | 0.5741 | 0.8611 | 0.7654 | 0.455 |
| 215 | 0.5741 | 0.8657 | 0.7685 | 0.4613 |
| 216 | 0.5741 | 0.8657 | 0.7685 | 0.4613 |
| 217 | 0.5833 | 0.8657 | 0.7716 | 0.4695 |
| 218 | 0.5833 | 0.8657 | 0.7716 | 0.4695 |
| 219 | 0.5833 | 0.8657 | 0.7716 | 0.4695 |
| 220 | 0.5463 | 0.8704 | 0.7623 | 0.4432 |
| 221 | 0.5463 | 0.8704 | 0.7623 | 0.4432 |
| 222 | 0.5648 | 0.8704 | 0.7685 | 0.4596 |
| 223 | 0.5556 | 0.8704 | 0.7654 | 0.4514 |
| 224 | 0.5556 | 0.8704 | 0.7654 | 0.4514 |
| 225 | 0.5556 | 0.8704 | 0.7654 | 0.4514 |
| 226 | 0.5556 | 0.8704 | 0.7654 | 0.4514 |
| 227 | 0.5556 | 0.8704 | 0.7654 | 0.4514 |
| 228 | 0.5278 | 0.8657 | 0.7531 | 0.4201 |
| 229 | 0.5278 | 0.8657 | 0.7531 | 0.4201 |
| 230 | 0.5278 | 0.8657 | 0.7531 | 0.4201 |
| 231 | 0.5278 | 0.8611 | 0.75 | 0.4136 |
| 232 | 0.5278 | 0.8611 | 0.75 | 0.4136 |
| 233 | 0.5278 | 0.8611 | 0.75 | 0.4136 |
| 234 | 0.5185 | 0.8611 | 0.7469 | 0.4053 |
| 235 | 0.5185 | 0.8611 | 0.7469 | 0.4053 |
| 236 | 0.5185 | 0.8611 | 0.7469 | 0.4053 |
| 237 | 0.5278 | 0.8611 | 0.75 | 0.4136 |
| 238 | 0.5185 | 0.8704 | 0.7531 | 0.4183 |
| 239 | 0.5185 | 0.8704 | 0.7531 | 0.4183 |
| 240 | 0.5185 | 0.8704 | 0.7531 | 0.4183 |
| 241 | 0.5185 | 0.8611 | 0.7469 | 0.4053 |
| 242 | 0.5 | 0.8657 | 0.7438 | 0.395 |
| 243 | 0.5 | 0.8657 | 0.7438 | 0.395 |
| 244 | 0.5 | 0.8657 | 0.7438 | 0.395 |
| 245 | 0.5 | 0.8657 | 0.7438 | 0.395 |
| 246 | 0.5 | 0.8657 | 0.7438 | 0.395 |
| 247 | 0.5 | 0.8565 | 0.7377 | 0.382 |
| 248 | 0.5 | 0.8565 | 0.7377 | 0.382 |
| 249 | 0.5 | 0.8565 | 0.7377 | 0.382 |
| 250 | 0.5185 | 0.8519 | 0.7407 | 0.3925 |
| 251 | 0.5185 | 0.8519 | 0.7407 | 0.3925 |
| 252 | 0.5185 | 0.8472 | 0.7377 | 0.3863 |
| 253 | 0.5 | 0.8472 | 0.7315 | 0.3693 |
| 254 | 0.5 | 0.8472 | 0.7315 | 0.3693 |
| 255 | 0.5 | 0.8472 | 0.7315 | 0.3693 |
| 256 | 0.5 | 0.8472 | 0.7315 | 0.3693 |
| 257 | 0.5 | 0.8472 | 0.7315 | 0.3693 |
| 258 | 0.5 | 0.8519 | 0.7346 | 0.3756 |
| 259 | 0.5 | 0.8519 | 0.7346 | 0.3756 |
| 260 | 0.5093 | 0.8472 | 0.7346 | 0.3778 |
| 261 | 0.5093 | 0.8519 | 0.7377 | 0.3841 |
| 262 | 0.5093 | 0.8519 | 0.7377 | 0.3841 |
| 263 | 0.5093 | 0.8611 | 0.7438 | 0.3969 |
| 264 | 0.5093 | 0.8611 | 0.7438 | 0.3969 |
| 265 | 0.5093 | 0.8704 | 0.75 | 0.41 |
| 266 | 0.5093 | 0.8796 | 0.7562 | 0.4234 |
| 267 | 0.5093 | 0.8796 | 0.7562 | 0.4234 |
| 268 | 0.5093 | 0.8796 | 0.7562 | 0.4234 |
| 269 | 0.5093 | 0.8796 | 0.7562 | 0.4234 |
| 270 | 0.5093 | 0.8796 | 0.7562 | 0.4234 |
| 271 | 0.5093 | 0.8796 | 0.7562 | 0.4234 |
| 272 | 0.5093 | 0.8796 | 0.7562 | 0.4234 |
| 273 | 0.5093 | 0.8796 | 0.7562 | 0.4234 |
| 274 | 0.5093 | 0.8796 | 0.7562 | 0.4234 |
| 275 | 0.5185 | 0.8704 | 0.7531 | 0.4183 |
| 276 | 0.5185 | 0.8704 | 0.7531 | 0.4183 |
| 277 | 0.5185 | 0.875 | 0.7562 | 0.425 |
| 278 | 0.5185 | 0.875 | 0.7562 | 0.425 |
| 279 | 0.5185 | 0.875 | 0.7562 | 0.425 |
| 280 | 0.5185 | 0.875 | 0.7562 | 0.425 |
| 281 | 0.5185 | 0.875 | 0.7562 | 0.425 |
| 282 | 0.5185 | 0.8704 | 0.7531 | 0.4183 |
| 283 | 0.537 | 0.8657 | 0.7562 | 0.4284 |
| 284 | 0.537 | 0.8657 | 0.7562 | 0.4284 |
| 285 | 0.537 | 0.8704 | 0.7593 | 0.4349 |
| 286 | 0.5278 | 0.8704 | 0.7562 | 0.4267 |
| 287 | 0.5278 | 0.8704 | 0.7562 | 0.4267 |
| 288 | 0.5278 | 0.8704 | 0.7562 | 0.4267 |
| 289 | 0.5278 | 0.8704 | 0.7562 | 0.4267 |
| 290 | 0.5463 | 0.8704 | 0.7623 | 0.4432 |
| 291 | 0.537 | 0.8704 | 0.7593 | 0.4349 |
| 292 | 0.537 | 0.8704 | 0.7593 | 0.4349 |
| 293 | 0.537 | 0.8704 | 0.7593 | 0.4349 |
| 294 | 0.537 | 0.8704 | 0.7593 | 0.4349 |
| 295 | 0.537 | 0.8704 | 0.7593 | 0.4349 |
| 296 | 0.537 | 0.8704 | 0.7593 | 0.4349 |
| 297 | 0.537 | 0.8704 | 0.7593 | 0.4349 |
| 298 | 0.537 | 0.8704 | 0.7593 | 0.4349 |
| 299 | 0.537 | 0.8704 | 0.7593 | 0.4349 |
| 300 | 0.537 | 0.8704 | 0.7593 | 0.4349 |
| 301 | 0.537 | 0.8704 | 0.7593 | 0.4349 |
| 302 | 0.5093 | 0.8704 | 0.75 | 0.41 |
| 303 | 0.5093 | 0.8704 | 0.75 | 0.41 |
| 304 | 0.5093 | 0.8704 | 0.75 | 0.41 |
| 305 | 0.5093 | 0.8704 | 0.75 | 0.41 |
| 306 | 0.5093 | 0.8704 | 0.75 | 0.41 |
| 307 | 0.5093 | 0.8704 | 0.75 | 0.41 |
| 308 | 0.5093 | 0.8704 | 0.75 | 0.41 |
| 309 | 0.5093 | 0.8704 | 0.75 | 0.41 |
| 310 | 0.5093 | 0.8704 | 0.75 | 0.41 |
| 311 | 0.5093 | 0.8704 | 0.75 | 0.41 |
| 312 | 0.5093 | 0.8704 | 0.75 | 0.41 |
| 313 | 0.5093 | 0.8704 | 0.75 | 0.41 |
| 314 | 0.5093 | 0.8704 | 0.75 | 0.41 |
| 315 | 0.5093 | 0.8704 | 0.75 | 0.41 |
| 316 | 0.4907 | 0.8657 | 0.7407 | 0.3865 |
| 317 | 0.4907 | 0.8657 | 0.7407 | 0.3865 |
| 318 | 0.5 | 0.8704 | 0.7469 | 0.4016 |
| 319 | 0.5 | 0.8704 | 0.7469 | 0.4016 |
| 320 | 0.5 | 0.8704 | 0.7469 | 0.4016 |
| 321 | 0.5 | 0.8704 | 0.7469 | 0.4016 |
| 322 | 0.5 | 0.8704 | 0.7469 | 0.4016 |
| 323 | 0.5 | 0.8704 | 0.7469 | 0.4016 |
| 324 | 0.5 | 0.8704 | 0.7469 | 0.4016 |
| 325 | 0.5 | 0.8704 | 0.7469 | 0.4016 |
| 326 | 0.5 | 0.8704 | 0.7469 | 0.4016 |
| 327 | 0.5 | 0.8704 | 0.7469 | 0.4016 |
| 328 | 0.5093 | 0.8704 | 0.75 | 0.41 |
| 329 | 0.5185 | 0.8704 | 0.7531 | 0.4183 |
| 330 | 0.5185 | 0.8704 | 0.7531 | 0.4183 |
| 331 | 0.5185 | 0.8704 | 0.7531 | 0.4183 |
| 332 | 0.5463 | 0.8704 | 0.7623 | 0.4432 |
| 333 | 0.5463 | 0.8704 | 0.7623 | 0.4432 |
| 334 | 0.5463 | 0.8704 | 0.7623 | 0.4432 |
| 335 | 0.5463 | 0.8704 | 0.7623 | 0.4432 |
| 336 | 0.5463 | 0.8704 | 0.7623 | 0.4432 |
| 337 | 0.5463 | 0.8704 | 0.7623 | 0.4432 |
| 338 | 0.5556 | 0.875 | 0.7685 | 0.458 |
| 339 | 0.5556 | 0.875 | 0.7685 | 0.458 |
| 340 | 0.5556 | 0.875 | 0.7685 | 0.458 |
| 341 | 0.5556 | 0.875 | 0.7685 | 0.458 |
| 342 | 0.5556 | 0.875 | 0.7685 | 0.458 |
| 343 | 0.5556 | 0.875 | 0.7685 | 0.458 |
| 344 | 0.5556 | 0.875 | 0.7685 | 0.458 |
| 345 | 0.5556 | 0.875 | 0.7685 | 0.458 |
| 346 | 0.5556 | 0.875 | 0.7685 | 0.458 |
| 347 | 0.5556 | 0.875 | 0.7685 | 0.458 |
| 348 | 0.5556 | 0.875 | 0.7685 | 0.458 |
| 349 | 0.5556 | 0.875 | 0.7685 | 0.458 |
| 350 | 0.5463 | 0.875 | 0.7654 | 0.4498 |
| 351 | 0.5463 | 0.875 | 0.7654 | 0.4498 |
| 352 | 0.5463 | 0.875 | 0.7654 | 0.4498 |
| 353 | 0.5463 | 0.875 | 0.7654 | 0.4498 |
| 354 | 0.5463 | 0.875 | 0.7654 | 0.4498 |
| 355 | 0.5463 | 0.875 | 0.7654 | 0.4498 |
| 356 | 0.5463 | 0.875 | 0.7654 | 0.4498 |
| 357 | 0.5463 | 0.875 | 0.7654 | 0.4498 |
| 358 | 0.5463 | 0.8704 | 0.7623 | 0.4432 |
| 359 | 0.5463 | 0.8704 | 0.7623 | 0.4432 |
| 360 | 0.5463 | 0.8704 | 0.7623 | 0.4432 |
| 361 | 0.5463 | 0.8704 | 0.7623 | 0.4432 |
| 362 | 0.5463 | 0.8704 | 0.7623 | 0.4432 |
| 363 | 0.5463 | 0.875 | 0.7654 | 0.4498 |
| 364 | 0.5556 | 0.875 | 0.7685 | 0.458 |
| 365 | 0.5556 | 0.875 | 0.7685 | 0.458 |
| 366 | 0.5556 | 0.875 | 0.7685 | 0.458 |
| 367 | 0.5556 | 0.875 | 0.7685 | 0.458 |
| 368 | 0.5556 | 0.875 | 0.7685 | 0.458 |
| 369 | 0.5556 | 0.875 | 0.7685 | 0.458 |
| 370 | 0.5556 | 0.875 | 0.7685 | 0.458 |
| 371 | 0.5648 | 0.8704 | 0.7685 | 0.4596 |
| 372 | 0.5648 | 0.8704 | 0.7685 | 0.4596 |
| 373 | 0.5556 | 0.875 | 0.7685 | 0.458 |
| 374 | 0.5556 | 0.875 | 0.7685 | 0.458 |
| 375 | 0.5741 | 0.8796 | 0.7778 | 0.4809 |
| 376 | 0.5741 | 0.8796 | 0.7778 | 0.4809 |
| 377 | 0.5648 | 0.8843 | 0.7778 | 0.4794 |
| 378 | 0.5648 | 0.8843 | 0.7778 | 0.4794 |
| 379 | 0.5648 | 0.8843 | 0.7778 | 0.4794 |
| 380 | 0.5648 | 0.8796 | 0.7747 | 0.4727 |
| 381 | 0.5648 | 0.8796 | 0.7747 | 0.4727 |
| 382 | 0.5741 | 0.8796 | 0.7778 | 0.4809 |
| 383 | 0.5741 | 0.8796 | 0.7778 | 0.4809 |
| 384 | 0.5741 | 0.8796 | 0.7778 | 0.4809 |
| 385 | 0.5556 | 0.8796 | 0.7716 | 0.4646 |
| 386 | 0.5556 | 0.8796 | 0.7716 | 0.4646 |
| 387 | 0.5556 | 0.8796 | 0.7716 | 0.4646 |
| 388 | 0.5741 | 0.8796 | 0.7778 | 0.4809 |
| 389 | 0.5741 | 0.875 | 0.7747 | 0.4743 |
| 390 | 0.5741 | 0.875 | 0.7747 | 0.4743 |
| 391 | 0.5741 | 0.875 | 0.7747 | 0.4743 |
| 392 | 0.5741 | 0.875 | 0.7747 | 0.4743 |
| 393 | 0.5741 | 0.875 | 0.7747 | 0.4743 |
| 394 | 0.5741 | 0.875 | 0.7747 | 0.4743 |
| 395 | 0.5833 | 0.8704 | 0.7747 | 0.4759 |
| 396 | 0.5833 | 0.8704 | 0.7747 | 0.4759 |
| 397 | 0.5833 | 0.875 | 0.7778 | 0.4824 |
| 398 | 0.5833 | 0.875 | 0.7778 | 0.4824 |
| 399 | 0.5833 | 0.875 | 0.7778 | 0.4824 |
| 400 | 0.5833 | 0.875 | 0.7778 | 0.4824 |
| 401 | 0.5833 | 0.875 | 0.7778 | 0.4824 |
| 402 | 0.5833 | 0.875 | 0.7778 | 0.4824 |
| 403 | 0.5833 | 0.875 | 0.7778 | 0.4824 |
| 404 | 0.5833 | 0.875 | 0.7778 | 0.4824 |
| 405 | 0.5833 | 0.875 | 0.7778 | 0.4824 |
| 406 | 0.5741 | 0.875 | 0.7747 | 0.4743 |
| 407 | 0.5741 | 0.875 | 0.7747 | 0.4743 |
| 408 | 0.5741 | 0.875 | 0.7747 | 0.4743 |
| 409 | 0.5741 | 0.875 | 0.7747 | 0.4743 |
| 410 | 0.5741 | 0.875 | 0.7747 | 0.4743 |
| 411 | 0.5741 | 0.875 | 0.7747 | 0.4743 |
| 412 | 0.5741 | 0.875 | 0.7747 | 0.4743 |
| 413 | 0.5741 | 0.875 | 0.7747 | 0.4743 |
| 414 | 0.5741 | 0.875 | 0.7747 | 0.4743 |
| 415 | 0.5741 | 0.875 | 0.7747 | 0.4743 |
| 416 | 0.5741 | 0.875 | 0.7747 | 0.4743 |
| 417 | 0.5741 | 0.875 | 0.7747 | 0.4743 |
| 418 | 0.5741 | 0.875 | 0.7747 | 0.4743 |
| 419 | 0.5741 | 0.875 | 0.7747 | 0.4743 |
| 420 | 0.5741 | 0.875 | 0.7747 | 0.4743 |
| 421 | 0.5741 | 0.875 | 0.7747 | 0.4743 |
| 422 | 0.5741 | 0.8704 | 0.7716 | 0.4678 |
| 423 | 0.5741 | 0.875 | 0.7747 | 0.4743 |
| 424 | 0.5741 | 0.8704 | 0.7716 | 0.4678 |
| 425 | 0.5741 | 0.8704 | 0.7716 | 0.4678 |
| 426 | 0.5741 | 0.8704 | 0.7716 | 0.4678 |
| 427 | 0.5833 | 0.8704 | 0.7747 | 0.4759 |
| 428 | 0.5833 | 0.8704 | 0.7747 | 0.4759 |
| 429 | 0.5833 | 0.8704 | 0.7747 | 0.4759 |
| 430 | 0.5833 | 0.875 | 0.7778 | 0.4824 |
| 431 | 0.5926 | 0.8704 | 0.7778 | 0.484 |
| 432 | 0.5833 | 0.875 | 0.7778 | 0.4824 |
| 433 | 0.5833 | 0.875 | 0.7778 | 0.4824 |
| 434 | 0.5833 | 0.875 | 0.7778 | 0.4824 |
| 435 | 0.5833 | 0.875 | 0.7778 | 0.4824 |
| 436 | 0.5833 | 0.8796 | 0.7809 | 0.4889 |
| 437 | 0.5833 | 0.8796 | 0.7809 | 0.4889 |
| 438 | 0.5833 | 0.8796 | 0.7809 | 0.4889 |
| 439 | 0.5741 | 0.8796 | 0.7778 | 0.4809 |
| 440 | 0.5833 | 0.8796 | 0.7809 | 0.4889 |
| 441 | 0.6019 | 0.8796 | 0.787 | 0.505 |
| 442 | 0.5926 | 0.8796 | 0.784 | 0.497 |
| 443 | 0.5926 | 0.8796 | 0.784 | 0.497 |
| 444 | 0.5926 | 0.8796 | 0.784 | 0.497 |
| 445 | 0.5926 | 0.8796 | 0.784 | 0.497 |
| 446 | 0.5926 | 0.8796 | 0.784 | 0.497 |
| 447 | 0.5926 | 0.8796 | 0.784 | 0.497 |
| 448 | 0.5926 | 0.8796 | 0.784 | 0.497 |
| 449 | 0.5741 | 0.875 | 0.7747 | 0.4743 |
| 450 | 0.5741 | 0.875 | 0.7747 | 0.4743 |
| 451 | 0.5741 | 0.875 | 0.7747 | 0.4743 |
| 452 | 0.5741 | 0.875 | 0.7747 | 0.4743 |
| 453 | 0.5833 | 0.8704 | 0.7747 | 0.4759 |
| 454 | 0.5833 | 0.8704 | 0.7747 | 0.4759 |
| 455 | 0.5833 | 0.8704 | 0.7747 | 0.4759 |
| 456 | 0.5926 | 0.875 | 0.7809 | 0.4905 |
| 457 | 0.5926 | 0.875 | 0.7809 | 0.4905 |
| 458 | 0.5926 | 0.875 | 0.7809 | 0.4905 |
| 459 | 0.5926 | 0.875 | 0.7809 | 0.4905 |
| 460 | 0.5926 | 0.875 | 0.7809 | 0.4905 |
| 461 | 0.5926 | 0.875 | 0.7809 | 0.4905 |
| 462 | 0.5926 | 0.875 | 0.7809 | 0.4905 |
| 463 | 0.5926 | 0.875 | 0.7809 | 0.4905 |
| 464 | 0.5926 | 0.875 | 0.7809 | 0.4905 |
| 465 | 0.5926 | 0.875 | 0.7809 | 0.4905 |
| 466 | 0.5926 | 0.8704 | 0.7778 | 0.484 |
| 467 | 0.5926 | 0.8704 | 0.7778 | 0.484 |
| 468 | 0.5926 | 0.8704 | 0.7778 | 0.484 |
| 469 | 0.5926 | 0.8704 | 0.7778 | 0.484 |
| 470 | 0.5926 | 0.8704 | 0.7778 | 0.484 |
| 471 | 0.5833 | 0.8704 | 0.7747 | 0.4759 |
| 472 | 0.5833 | 0.8704 | 0.7747 | 0.4759 |
| 473 | 0.5833 | 0.8704 | 0.7747 | 0.4759 |
| 474 | 0.5926 | 0.8704 | 0.7778 | 0.484 |
| 475 | 0.5926 | 0.8704 | 0.7778 | 0.484 |
| 476 | 0.5926 | 0.8796 | 0.784 | 0.497 |
| 477 | 0.5926 | 0.8796 | 0.784 | 0.497 |
| 478 | 0.5926 | 0.8796 | 0.784 | 0.497 |
| 479 | 0.5741 | 0.8796 | 0.7778 | 0.4809 |
| 480 | 0.5741 | 0.8796 | 0.7778 | 0.4809 |
| 481 | 0.5741 | 0.8796 | 0.7778 | 0.4809 |
| 482 | 0.5741 | 0.8796 | 0.7778 | 0.4809 |
| 483 | 0.5741 | 0.8796 | 0.7778 | 0.4809 |
| 484 | 0.5741 | 0.8843 | 0.7809 | 0.4875 |
| 485 | 0.5741 | 0.8843 | 0.7809 | 0.4875 |
| 486 | 0.5741 | 0.8843 | 0.7809 | 0.4875 |
| 487 | 0.5741 | 0.8843 | 0.7809 | 0.4875 |
| 488 | 0.5741 | 0.8843 | 0.7809 | 0.4875 |
| 489 | 0.5741 | 0.8843 | 0.7809 | 0.4875 |
| 490 | 0.5741 | 0.8843 | 0.7809 | 0.4875 |
| 491 | 0.5741 | 0.8843 | 0.7809 | 0.4875 |
| 492 | 0.5741 | 0.8843 | 0.7809 | 0.4875 |
| 493 | 0.5741 | 0.8843 | 0.7809 | 0.4875 |
| 494 | 0.5741 | 0.8843 | 0.7809 | 0.4875 |
| 495 | 0.5833 | 0.8889 | 0.787 | 0.5023 |
| 496 | 0.5833 | 0.8889 | 0.787 | 0.5023 |
| 497 | 0.5926 | 0.8796 | 0.784 | 0.497 |
| 498 | 0.5926 | 0.8796 | 0.784 | 0.497 |
| 499 | 0.5926 | 0.8796 | 0.784 | 0.497 |
| 500 | 0.5926 | 0.8796 | 0.784 | 0.497 |
